# mRAVE governs lysosomal catabolism through basal and mTORC1-regulated V-ATPase assembly

**DOI:** 10.1038/s44318-026-00838-5

**Published:** 2026-06-22

**Authors:** Nora S Siefert, Andrea Zanotti, Anastasija Paneva, Martin Schneider, Dominic Helm, Wilhelm Palm

**Affiliations:** 1 Division of Cell Signaling and Metabolism, German Cancer Research Center (DKFZ) and DKFZ-ZMBH Alliance, Heidelberg, Germany; 2 Faculty of Biosciences, Heidelberg University, Heidelberg, Germany; 3 Proteomics Core Facility, German Cancer Research Center (DKFZ), Heidelberg, Germany

**Keywords:** Membranes & Trafficking, Organelles

## Abstract

Acidification of lysosomes, endosomes and the Golgi underpins organelle-specific functions within the endomembrane system. This process is driven by vacuolar-type H^ +^ -ATPases (V-ATPases), proton pumps that reversibly assemble from peripheral V_1_ and membrane-integral V_o_ domains to regulate organelle pH. In yeast, V_1_–V_o_ assembly at the vacuole is mediated by the RAVE complex, but V-ATPase assembly in mammalian cells remains less well understood. Here, we systematically characterize physiological roles of the mammalian RAVE complex, composed of the subunits Dmxl1 or Dmxl2, Wdr7 and Rogdi. Under basal conditions, mRAVE broadly promotes V-ATPase assembly and luminal acidification of endomembrane organelles. Upon mTORC1 inactivation, mRAVE is recruited to lysosomes and required for the resulting increase in V-ATPase assembly and catabolic activity. Loss of mRAVE disrupts organelle acidification, leading to suppression of lysosomal catabolism, accumulation of dysfunctional lysosomes and lysosomal exocytosis. Restoring lysosomal pH rescues basal function in mRAVE-deficient cells but not the mTORC1-regulated increase in catabolic activity. Thus, mRAVE is an essential V-ATPase assembly factor that couples acidification to organelle function and nutrient signaling.

## Synopsis

The V-ATPase acidifies lysosomes, endosomes and the Golgi and is regulated by reversible assembly of its V_1_ and V_o_ domains. This study establishes mRAVE as an essential assembly factor, coupling organelle acidification to lysosomal function and nutrient signaling.

- The mRAVE complex, composed of Dmxl1/2, Wdr7 and Rogdi, is broadly required for V_1_–V_o_ assembly throughout the endomembrane system, supporting basal acidification of lysosomes and the Golgi.
- Upon mTORC1 inhibition, mRAVE is recruited to lysosomes, where it promotes V-ATPase assembly, luminal acidification and catabolic activity.
- Loss of mRAVE disrupts organelle acidification, leading to the accumulation of dysfunctional lysosomes and induction of lysosomal exocytosis.
- Restoring lysosomal pH in the absence of mRAVE rescues basal lysosomal function but not mTORC1-dependent enhancement of catabolic activity.

## Abstract (web summary)

Cells tune lysosomal and endomembrane function through mRAVE dynamically controlling proton pump assembly in response to nutrient state.

## Graphical abstract

**Figure:**
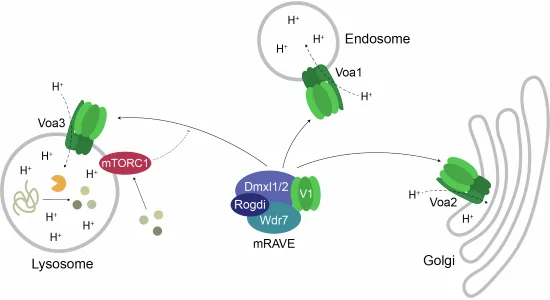

**Figure:**
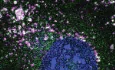

## Introduction

Luminal acidification of organelles is a central organizing principle through which eukaryotic cells establish functionally distinct compartments (Freeman et al, 2023). The most acidic compartment in mammalian cells is the lysosome, a catabolic organelle that contains numerous hydrolytic enzymes specialized for macromolecular breakdown (Ballabio and Bonifacino, 2020; Settembre and Perera, 2024). Several other organelles of the endomembrane system, including the Golgi apparatus and various endosomal compartments, also maintain a distinct acidic pH. These differences in luminal pH enable organelle-specific biochemical reactions and support diverse subcellular processes, such as membrane trafficking, receptor-ligand dissociation and signal transduction (Freeman et al, 2023). Consequently, perturbations in organelle acidification are linked to a range of human diseases, including cancer, neurodegeneration and genetic disorders, as well as aging (Eaton et al, 2021; Nixon and Rubinsztein, 2024; Tan and Finkel, 2023; Webb et al, 2021).

The acidification of organelles is mediated by vacuolar-type H^+^-ATPases (V-ATPases), multi-subunit proton pumps that use ATP hydrolysis to drive proton translocation across membranes (Collins and Forgac, 2020; Eaton et al, 2021; Vasanthakumar and Rubinstein, 2020). V-ATPases consist of two subcomplexes: a peripheral V_1_ domain, which hydrolyzes ATP, and a membrane-integral V_o_ domain, which translocates protons into the organelle lumen. The V_1_ and V_o_ domains exist in an equilibrium between separate complexes and the assembled V-ATPase. Regulation of V_1_–V_o_ assembly constitutes a conserved mechanism that enables cells to dynamically adjust the number of active proton pumps (Kane, 1995; McGuire and Forgac, 2018; Ratto et al, 2022; Sautin et al, 2005; Sumner et al, 1995; Trombetta et al, 2003). The V_o_ domain is targeted to different organelles by several ubiquitously expressed paralogs of V_o_ subunit a (Voa): Voa1 to endosomes and lysosomes, Voa2 to the Golgi and Voa3 (Tcirg1) to lysosomes. In specialized cell types, Voa subunits also direct the V_o_ domain to distinct compartments, such as synaptic vesicles in neurons or the plasma membrane in osteoclasts and macrophages. An additional paralog, Voa4, mediates plasma membrane targeting in proton-secreting cells of the kidney (Collins and Forgac, 2020; Vasanthakumar and Rubinstein, 2020). The regulation of V-ATPase subunit composition, subcellular localization and V_1_–V_o_ domain assembly appears to be important to ensure that organelles maintain the luminal pH required for their specific function.

V-ATPase activity is tightly linked to the function of lysosomes, establishing the acidic environment where the organelle’s catabolic enzymes are active (Ballabio and Bonifacino, 2020; Settembre and Perera, 2024). Lysosomes also integrate inputs from nutrient levels and growth factor signals to activate the kinase mechanistic target of rapamycin complex 1 (mTORC1), a central regulator of cellular metabolism and growth (Goul et al, 2023; Kim and Guan, 2019). When mTORC1 is active, a substantial fraction of lysosomal V-ATPases is disassembled; conversely, nutrient starvation and resulting mTORC1 inactivation trigger V_1_–V_o_ assembly, thereby rapidly increasing lysosomal acidification and enzyme activity (Palm et al, 2015; Ratto et al, 2022). Prolonged mTORC1 inactivation further promotes the expression of V-ATPase subunits via the transcription factor EB (TFEB) (Peña-Llopis et al, 2011). Together, these mechanisms couple nutrient signaling to acute and long-term adaptation of lysosomal catabolism.

Association of the V_1_ and V_o_ domains into a functional proton pump depends on dedicated assembly factors. In yeast, V-ATPase assembly is mediated by the RAVE (Regulator of H^+^-ATPase of Vacuolar and Endosomal membranes) complex, which consists of the central component Rav1 together with Rav2 and Skp1 (Jaskolka et al, 2021b; Seol et al, 2001). RAVE recruits cytosolic V_1_ domains to V_o_ domains at the vacuolar membrane and catalyzes their reassembly in response to glucose stimulation (Jaskolka et al, 2021a; Smardon et al, 2002). Mammals express two Rav1 homologs, Dmxl1 and Dmxl2, which associate with Wdr7—an unrelated protein not found in yeast RAVE (Jaskolka et al, 2021b; Kawabe et al, 2003). Early evidence implicating these proteins in V-ATPase assembly showed that Dmxl1, Dmxl2, and Wdr7 co-purify with the V-ATPase from mouse kidney and contribute to reacidification of neutralized lysosomes (Merkulova et al, 2015). Consistently, Dmxl1 and Dmxl2 co-depletion suppresses lysosomal V-ATPase assembly in cultured fibroblasts, while Dmxl1 depletion in mouse kidney disrupts V_1_ interaction with Voa4-containing V_o_ domains (Eaton et al, 2024; Ratto et al, 2022). Recent biochemical and structural studies identified Rogdi as the mammalian homolog of yeast Rav2 and defined a RAVE-related complex composed of Dmxl1 or Dmxl2, Wdr7 and Rogdi, referred to as metazoan RAVE (mRAVE) (Cho et al, 2022; Lee et al, 2025; Nardone et al, 2025; Winkley and Kane, 2025). mRAVE is recruited to damaged lysosomes with elevated pH and, in turn, promotes V-ATPase assembly to restore luminal acidification (Lee et al, 2025; Nardone et al, 2025). Whether mRAVE sustains V-ATPase assembly under basal conditions, promotes assembly in response to physiological cues and supports organelle function beyond the lysosomal stress response has been unclear.

Here, we systematically define the role of mRAVE in organelle acidification and function. We show that mRAVE is broadly required for V-ATPase assembly across the endomembrane system under basal conditions and for enhanced assembly at lysosomes in response to mTORC1 inactivation. Loss of mRAVE disrupts organelle acidification and suppresses lysosomal catabolism, leading to accumulation of dysfunctional lysosomes and lysosomal exocytosis. These findings establish mRAVE as a general and essential V-ATPase assembly factor that supports basal and adaptive organelle function.

## Results

### mRAVE is required for basal assembly of the lysosomal V-ATPase

**Figure EV1:**
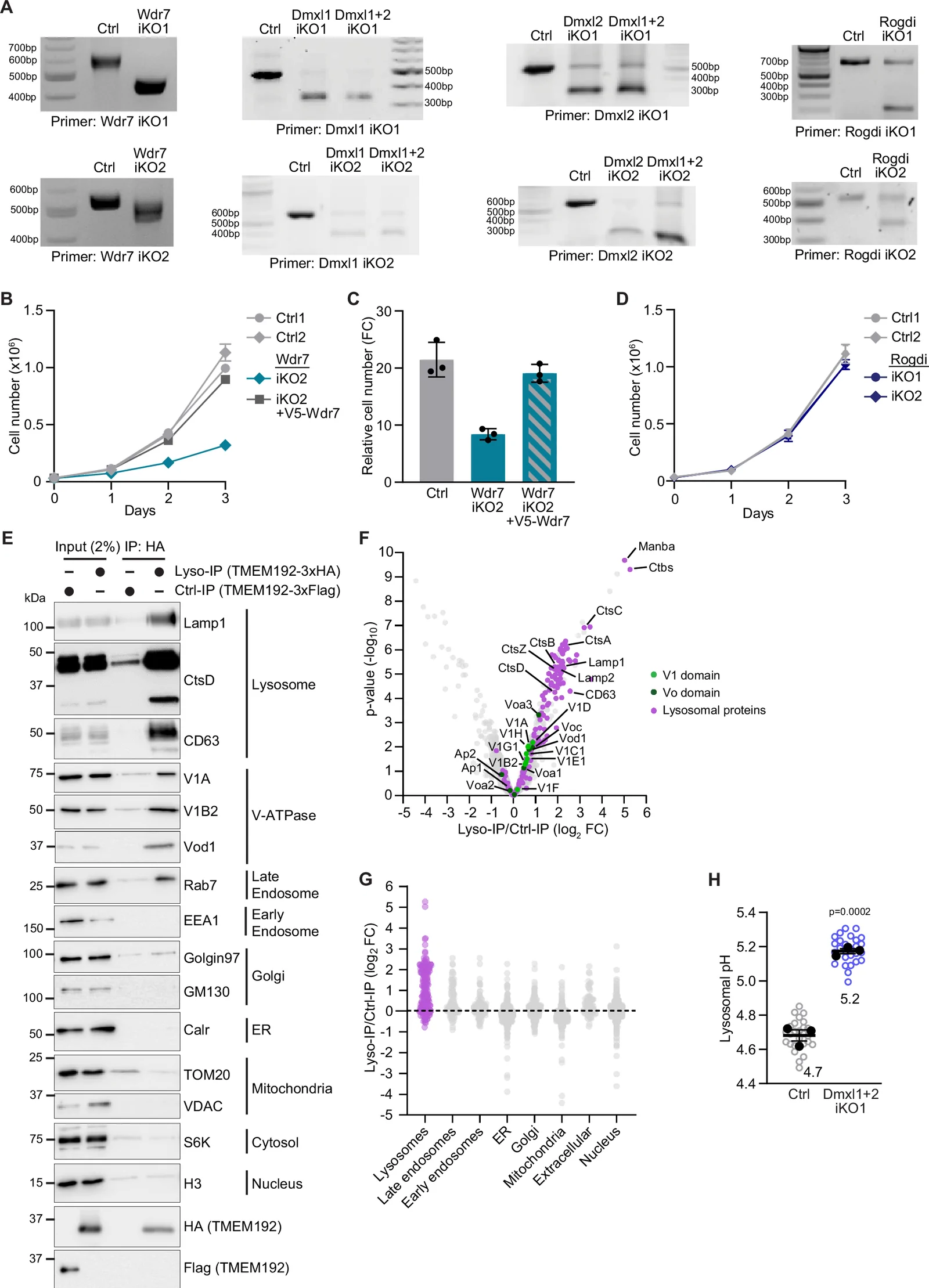
mRAVE CRISPR editing and Lyso-IP quality controls (related to Fig. 1). (**A**) CRISPR editing of mRAVE subunits in iKO MEFs cells after Cas9 induction for 4 days with doxycycline [0.5 µg/ml]. Successful editing was confirmed by PCR using primers that amplify the genomic locus spanning the cutting sites of the respective sgRNAs. Each gene was disrupted using two distinct sgRNA pairs. (**B**) Proliferation of Wdr7 iKO2 MEFs expressing sgRNA-resistant V5-Wdr7 cDNA. (**C**) Fold change (FC) in cell number of Wdr7 iKO2 RKO cells expressing sgRNA-resistant V5-Wdr7 cDNA after 3 days of proliferation. (**D**) Proliferation of Rogdi iKO MEFs. (**E**) Enrichment/depletion of organelle markers in Lyso-IP from MEFs, analyzed by immunoblotting. (**F**) Lysosome enrichment, analyzed by Lyso-IP and label-free LC–MS in MEFs. (**G**) Organelle enrichment/depletion, analyzed by Lyso-IP and label-free LC–MS in MEFs. (**H**) Lysosomal pH in Dmxl1 + 2 iKO MEFs, quantified by ratiometric imaging of 10 kDa Dextran Oregon Green and Alexa Fluor 647. (**B**–**D**) Data are mean ± SD (*n* = 3 technical replicates). (**F**,** G**) *n* = 4 independent experiments. (**H**) Data are replicate mean ± SEM (closed circles; *n* = 3 independent experiments) and fields of view (open circles; ≥9 per replicate). *p* values were calculated using a two-tailed unpaired *t*-test.

**Figure 1:**
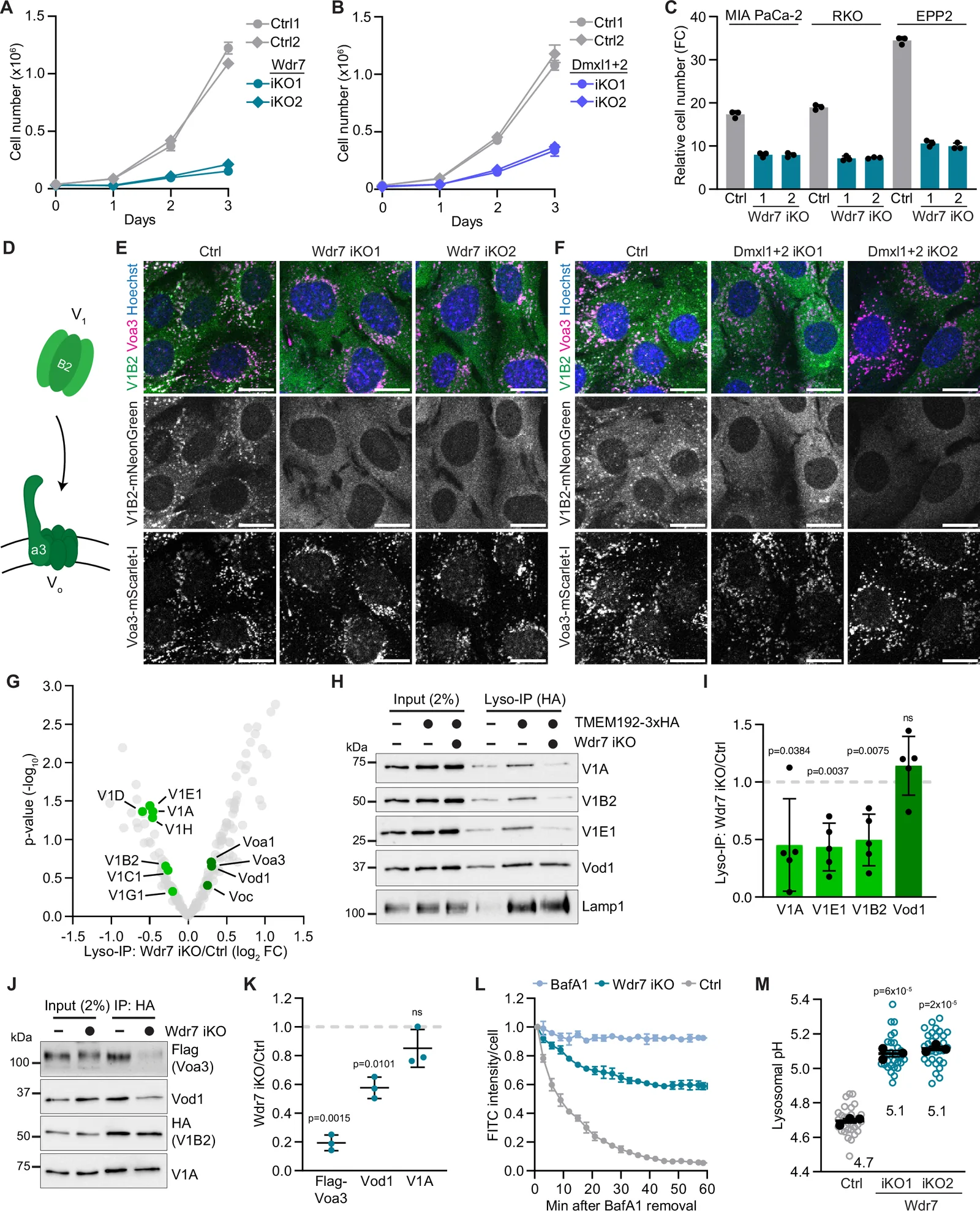
The mRAVE subunits Dmxl1/2 and Wdr7 are required for lysosomal V-ATPase assembly and acidification. (**A**,** B**) Proliferation of Wdr7 (**A**) and Dmxl1 + Dmxl2 (**B**) iKO MEFs. (**C**) Fold change (FC) in cell number of indicated Wdr7 iKO cell lines after 3 days of proliferation. (**D**) Schematic of V-ATPase V_1_–V_o_ domain assembly. (**E**,** F**) Subcellular localization of V1B2-mNeonGreen and Voa3-mScarlet-I in Wdr7 (**E**) and Dmxl1 + Dmxl2 (**F**) iKO MEFs, analyzed by live-cell imaging. (**G**) Changes in lysosomal proteins in Wdr7 iKO MEFs, analyzed by Lyso-IP and label-free LC–MS. (**H**) Changes in V-ATPase subunit abundance in Lyso-IPs from Wdr7 iKO MEFs, analyzed by immunoblotting. (**I**) Quantification of V-ATPase subunit abundance in Lyso-IP immunoblots as in (**H**). (**J**) HA co-IP in Wdr7 iKO MEFs expressing HA-V1B2 and Flag-Voa3, analyzed by immunoblotting for V-ATPase subunits. (**K**) Quantification of V-ATPase subunit abundance in co-IP immunoblots as in (**J**). (**L**) Fluorescence quenching of lysosomal FITC-dextran in Wdr7 iKO MEFs after 1 h bafilomycin A1 [20 nM] and subsequent bafilomycin A1 removal (Ctrl and Wdr7 iKO) or continuous bafilomycin A1 treatment. (**M**) Lysosomal pH in Wdr7 iKO MEFs, quantified by ratiometric imaging of 10 kDa Dextran Oregon Green and Alexa Fluor 647. (**A**–**C**) Data are mean ± SD (*n* = 3 technical replicates). (**G**) *n* = 4 independent experiments. (**I**,** K**) Data are mean ± SD (*n* = 5 (**I**), *n* = 3 (**K**) independent experiments). (**L**) Data are mean ± SEM (*n* = 3 independent experiments). (**M**) Data are replicate mean ± SEM (closed circles; *n* = 3 independent experiments) and fields of view (open circles; ≥10 per replicate). *p* values were calculated using a two-tailed unpaired *t*-test with Welch correction (**I**,** K**) or a two-tailed unpaired *t*-test (**M**). Scale bars, 20 µm. Source data are available online for this figure.

To systematically define mRAVE functions in mammalian cells, we generated inducible CRISPR/Cas9 knockouts (iKO) for each subunit—Wdr7, Dmxl1 and Dmxl2 or Rogdi—in SV40 large T-immortalized mouse embryonic fibroblasts (MEFs) (Fig. EV1A). Cells deficient for Wdr7 or for both Dmxl1 and Dmxl2 proliferated at strongly reduced rates (Fig. 1A,B). Similar proliferation defects were observed across several mouse and human cell lines lacking Wdr7 (Fig. 1C), and these defects were rescued by expression of an sgRNA-resistant Wdr7 cDNA (Fig. EV1B,C). Loss of Rogdi had no overt effect (Fig. EV1D). Thus, Wdr7 and Dmxl1/2 are essential mammalian genes.

To determine whether mRAVE is required for V-ATPase assembly at lysosomes, we labeled the peripheral V_1_ domain and membrane-integral V_o_ domain by stable expression of V1B2-mNeonGreen and Voa3-mScarlet-I, respectively. In control cells, V1B2 partially co-localized with Voa3 but was also diffusely present throughout the cytosol, reflecting the co-occurrence of assembled and disassembled V-ATPase pools (Fig. 1D–F). In cells deficient for Wdr7 or for both Dmxl1 and Dmxl2, however, most V_1_ domains were present in the cytosol, with only a minor fraction localizing to Voa3-containing organelles. To examine endogenous V-ATPase subunits, we enriched lysosomes by immunoprecipitating the lysosomal membrane protein TMEM192-HA (Lyso-IP (Abu-Remaileh et al, 2017)) and analyzed the resulting fractions by immunoblotting as well as liquid chromatography–mass spectrometry (LC–MS) with label-free quantification (LFQ). Lyso-IP efficiently enriched lysosomal proteins, including V-ATPase subunits, while depleting proteins from non-lysosomal compartments, confirming high purity of lysosomal fractions (Fig. EV1E–G; Dataset EV1). The abundance of V_1_ domain subunits was significantly decreased in lysosomal fractions from Wdr7-deficient cells (Fig. 1G–I; Dataset EV2). In contrast, the V_o_ domain subunits were not decreased. To directly assess the physical association between V_1_ and V_o_ domains, we conducted co-immunoprecipitation (co-IP) experiments using HA-V1B2 as bait. V_o_ domain subunits were significantly reduced in HA-V1B2 immunoprecipitates from Wdr7-deficient cells, whereas interactions among V_1_ domain subunits remained intact (Fig. 1J,K).

We next examined the functional consequences of defective V_1_–V_o_ domain assembly. To measure V-ATPase-dependent acidification of the lysosomal lumen, we preloaded lysosomes with dextran-conjugated FITC, whose fluorescence is quenched at acidic pH, and then neutralized luminal pH with the V-ATPase inhibitor bafilomycin A1 (Ratto et al, 2022). In control cells, bafilomycin A1 washout promptly restored V-ATPase activity, resulting in rapid FITC quenching (Fig. 1L). In contrast, Wdr7-deficient cells exhibited a pronounced decrease in the rate and extent of FITC quenching, indicating diminished lysosomal V-ATPase activity. We next quantified lysosomal pH by ratiometric imaging of lysosomally loaded dextran-conjugated fluorophores: Oregon Green, whose fluorescence is quenched at acidic pH, and pH-insensitive Alexa Fluor 647. Lysosomal pH increased from ~4.7 to 5.1–5.2 in cells deficient for Wdr7 or for both Dmxl1 and Dmxl2 (Figs. 1M and EV1H). Thus, mRAVE is required to sustain basal assembly of lysosomal V-ATPases and, consequently, normal lysosomal acidification.

**Figure EV2:**
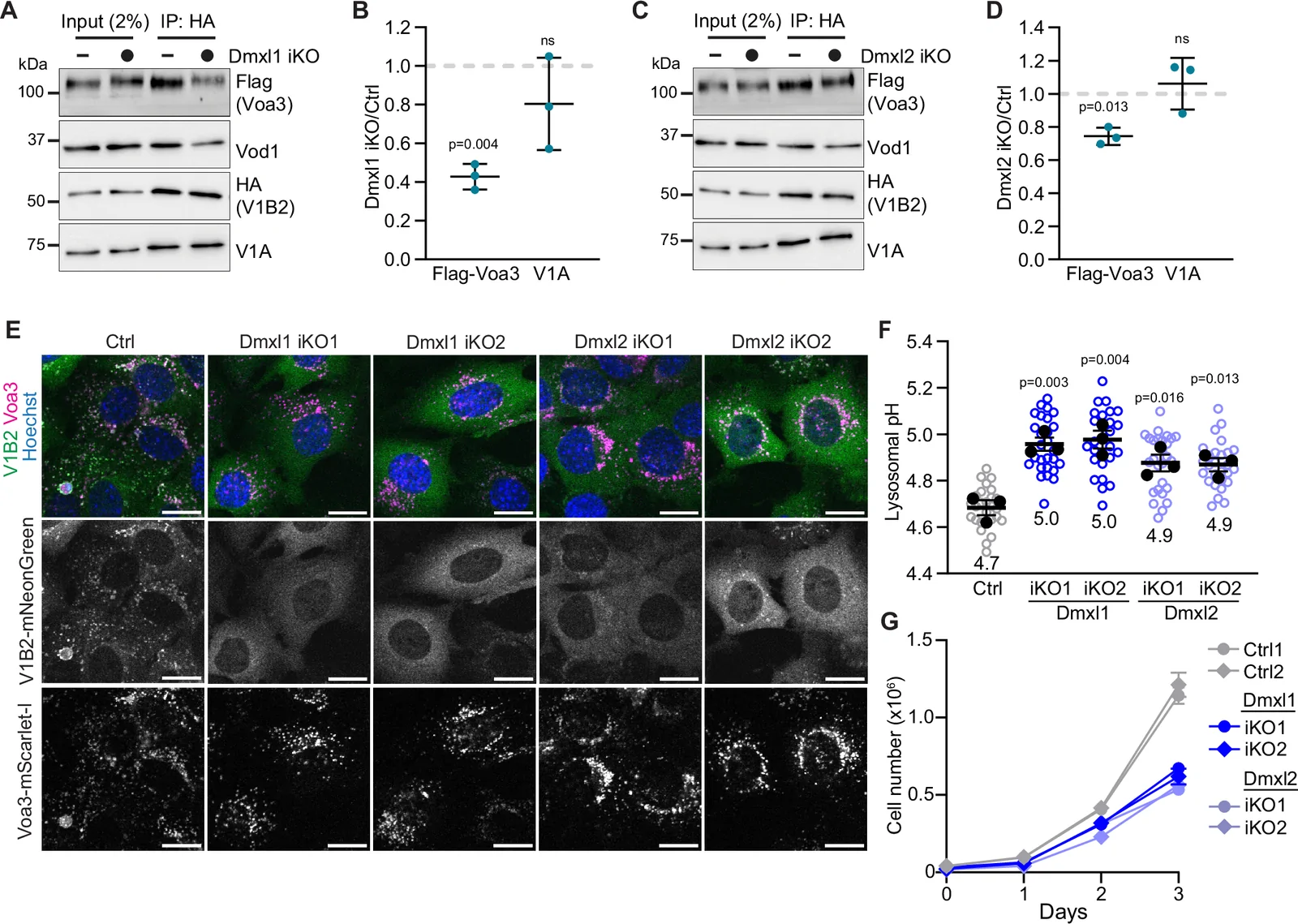
Dmxl1 and Dmxl2 have non-redundant functions (related to Fig. 1). (**A**) HA co-IP in Dmxl1 iKO MEFs expressing HA-V1B2 and Flag-Voa3, analyzed by immunoblotting for V-ATPase subunits. (**B**) Quantification of V-ATPase subunit abundance in co-IP immunoblots as in (**A**). (**C**) HA co-IP in Dmxl2 iKO MEFs expressing HA-V1B2 and Flag-Voa3, analyzed by immunoblotting for V-ATPase subunits. (**D**) Quantification of V-ATPase subunit abundance in co-IP immunoblots as in (**C**). (**E**) Subcellular localization of V1B2-mNeonGreen and Voa3-mScarlet-I in Dmxl1 iKO and Dmxl2 iKO MEFs, analyzed by live-cell imaging. (**F**) Lysosomal pH in Dmxl1 iKO and Dmxl2 iKO MEFs, quantified by ratiometric imaging of 10 kDa Dextran Oregon Green and Alexa Fluor 647. (**G**) Proliferation of Dmxl1 iKO and Dmxl2 iKO MEFs. (**B**,** D**) Data are mean ± SD (*n* = 3 independent experiments). (**F**) Data are replicate mean ± SEM (closed circles; *n* = 3 independent experiments) and fields of view (open circles; ≥10 per replicate). (**G**) Data are mean ± SD (*n* = 3 technical replicates). *p* values were calculated using a two-tailed unpaired *t*-test with Welch correction (**B**, **D**) or a two-tailed unpaired *t*-test (**F**). Scale bars, 20 µm.

To examine the contributions of the two Dmxl homologs, Dmxl1 and Dmxl2, we depleted each protein individually. Loss of either Dmxl1 or Dmxl2 reduced the interaction between V_1_ domains and Voa3-containing V_o_ domains (Fig. EV2A–D), shifted V1B2-mNeonGreen toward the cytosol (Fig. EV2E), elevated lysosomal pH (Fig. EV2F) and compromised cell proliferation (Fig. EV2G). Thus, cells lacking either Dmxl1 or Dmxl2 displayed similar phenotypes to those of cells deficient for both homologs, albeit less severe, suggesting partially non-redundant functions. Given the penetrant phenotypes resulting from loss of Wdr7, we focused subsequent analyses on Wdr7 as an essential mRAVE subunit.

### mRAVE is broadly required for V-ATPase assembly

**Figure EV3:**
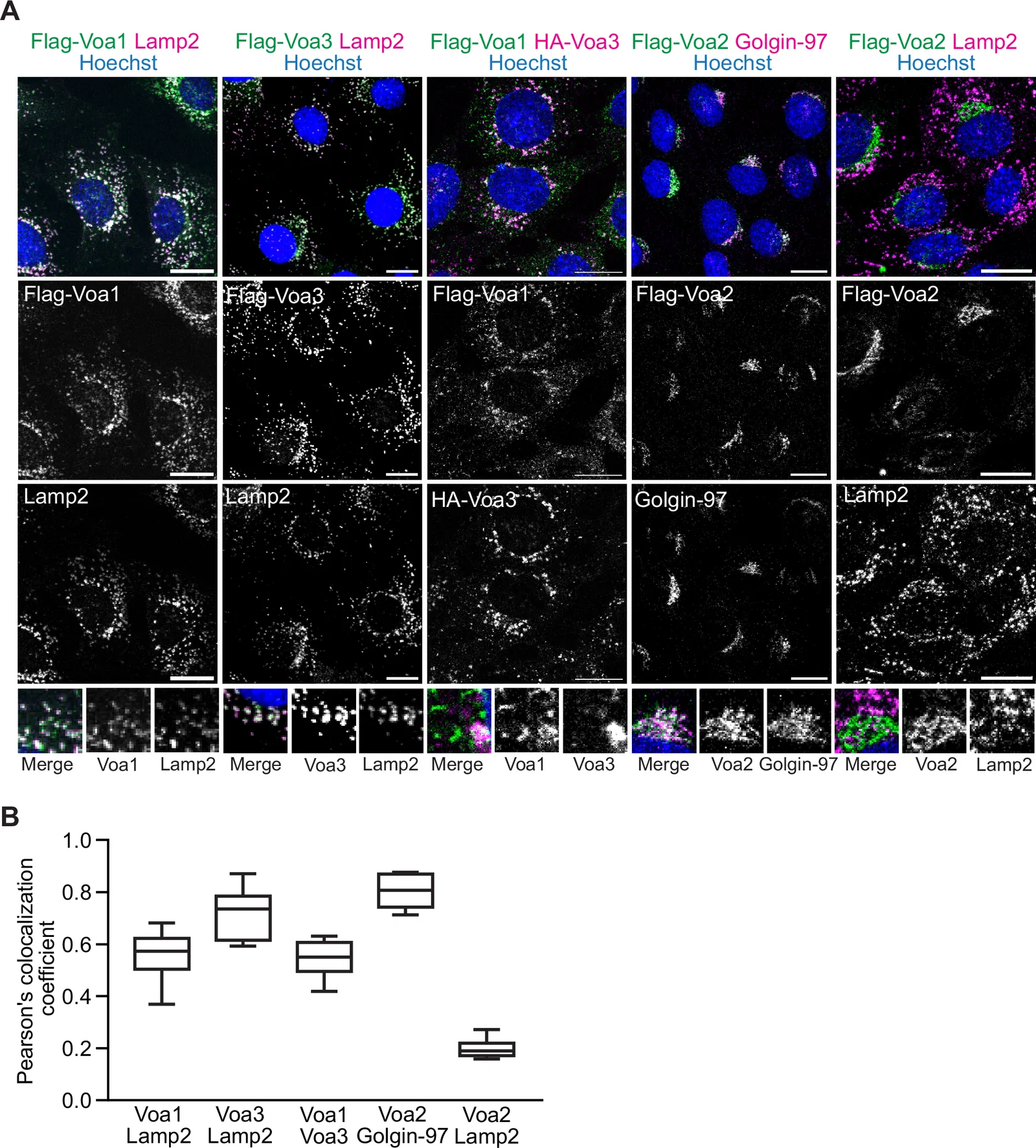
Subcellular localization of different Voa subunits (related to Fig. 2). (**A**) Colocalization of Flag-tagged Voa1, Voa2 and Voa3 with endogenous Lamp2 or Golgin-97 in MEFs, analyzed by immunofluorescence (IF). (**B**) Quantification of colocalization of cells shown in (**A**). (**B**) Data are median (center line), 75th and 25th percentiles (upper and lower bound of the box) and min to max range (whiskers) of *n* = 10 fields of views. Scale bars, 20 µm.

**Figure 2:**
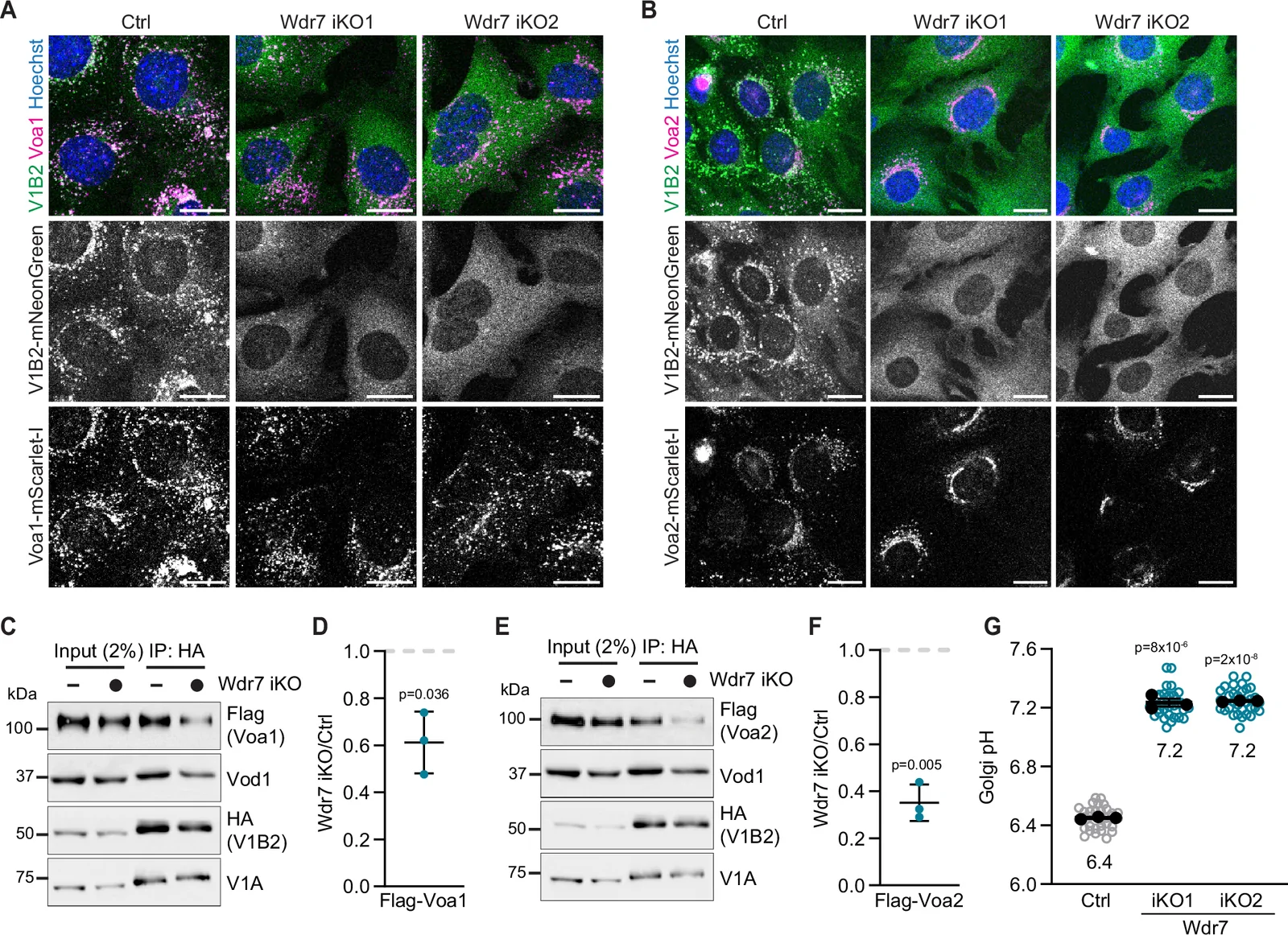
mRAVE supports V-ATPase assembly across different organelles. (**A**, **B**) Subcellular localization of V1B2-mNeonGreen and Voa1-mScarlet-I (**A**) or Voa2-mScarlet-I (**B**) in Wdr7 iKO MEFs, analyzed by live-cell imaging. (**C**) HA co-IP in Wdr7 iKO MEFs expressing HA-V1B2 and Flag-Voa1, analyzed by immunoblotting for V-ATPase subunits. (**D**) Quantification of V-ATPase subunit abundance in co-IP immunoblots as in (**C**). (**E**) HA co-IP in Wdr7 iKO MEFs expressing HA-V1B2 and Flag-Voa2, analyzed by immunoblotting for V-ATPase subunits. (**F**) Quantification of V-ATPase subunit abundance in co-IP immunoblots as in (**E**). (**G**) Golgi pH in Wdr7 iKO MEFs, quantified by ratiometric imaging of MGAT-SEP-mRuby. (**D**,** F**) Data are mean ± SD (*n* = 3 independent experiments). (**G**) Data are replicate mean ± SEM (closed circles; *n* = 3 independent experiments) and fields of view (open circles; ≥10 per replicate). *p* values were calculated using a two-tailed unpaired *t*-test with Welch correction (**D**,** F**) or a two-tailed unpaired *t*-test (**G**). Scale bars, 20 µm. Source data are available online for this figure.

In the absence of mRAVE, we observed a pronounced decrease in the assembly of Voa3-containing V-ATPases at lysosomes. To examine whether mRAVE assembles other V-ATPase complexes, we expressed fluorescently labeled Voa1, which localizes to the endolysosomal compartment, including Voa3-positive lysosomes, and Voa2, which localizes to the Golgi (Fig. EV3A,B) (Collins and Forgac, 2020; Vasanthakumar and Rubinstein, 2020). In control cells, V1B2-mNeonGreen showed robust colocalization with Voa1-mScarlet-I as well as Voa2-mScarlet-I (Fig. 2A,B). In Wdr7-deficient cells, however, very little colocalization of V1B2 with either Voa1- or Voa2-containing organelles was detectable. Consistently, HA-V1B2 immunoprecipitated significantly less Flag-Voa1 or Flag-Voa2 (Fig. 2C–F). To assess the functional impact of decreased V-ATPase assembly at the Golgi, we measured luminal pH using the Golgi-specific pH sensor MGAT-SEP-mRuby (Liu et al, 2023). Golgi pH increased from 6.4 in control cells to ~7.2 in Wdr7-deficient cells, indicating near-complete neutralization of the organelle lumen (Fig. 2G). We were unable to quantify endosomal pH due to the lack of compartment-specific pH sensors. Together, these results establish mRAVE as broadly required for the assembly of distinct V-ATPase complexes at different organelles.

### mRAVE promotes assembly of lysosomal V-ATPases in response to mTORC1 inhibition

**Figure 3:**
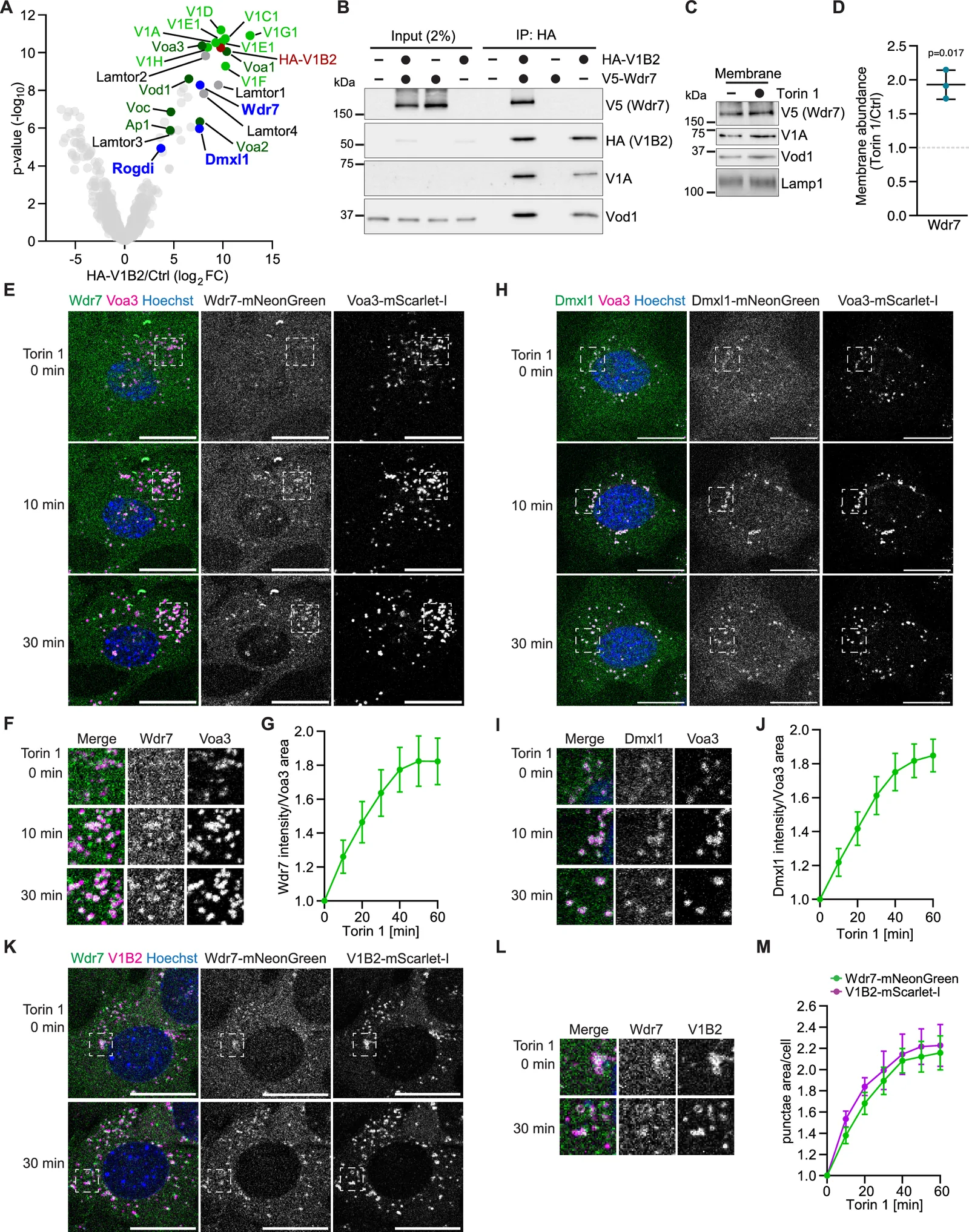
mTORC1 suppresses mRAVE recruitment to lysosomes. (**A**) HA co-IP in MEFs expressing HA-V1B2, analyzed by label-free LC–MS. (**B**) HA co-IP in MEFs expressing HA-V1B2 and V5-Wdr7, analyzed by immunoblotting. (**C**) V5-Wdr7 abundance in membrane fractions of dextran-loaded lysosomes from MEFs after 1 h ± torin 1 [400 nM], analyzed by immunoblotting. (**D**) Quantification of V5-Wdr7 abundance in immunoblots from membrane fractions as in (**C**). (**E**) Subcellular localization of Wdr7-mNeonGreen and Voa3-mScarlet-I in MEFs after addition of torin 1 [400 nM], analyzed by live-cell imaging. (**F**) Magnification of areas highlighted in (**E**). (**G**) Change of Wdr7-mNeonGreen intensity in Voa3-mScarlet-I-containing lysosomes, quantified by live-cell imaging as in (**E**). (**H**) Subcellular localization of Dmxl1-mNeonGreen and Voa3-mScarlet-I in MEFs after addition of torin 1 [400 nM], analyzed by live-cell imaging. (**I**) Magnification of areas highlighted in (**H**). (**J**) Change of Dmxl1-mNeonGreen intensity in Voa3-mScarlet-I-containing lysosomes, quantified by live-cell imaging as in (**H**). (**K**) Subcellular localization of Wdr7-mNeonGreen and V1B2-mScarlet-I in MEFs after addition of torin 1 [400 nM], analyzed by live-cell imaging. (**L**) Magnification of areas highlighted in (**K**). (**M**) Change in organellar Wdr7-mNeonGreen and V1B2-mScarlet-I, quantified by live-cell imaging as in (**K**). (**A**) *n* = 5 (HA-V1B2) and *n* = 4 (Ctrl) independent experiments. (**D**) Data are mean ± SD (*n* = 3 independent experiments). (**G**,** J**,** M**) Data are mean ± SD (≥15,000 organelles in ≥18 fields of view with a total of ≥160 cells). Scale bars, 10 µm. Source data are available online for this figure.

To further define the role of mRAVE in V-ATPase assembly, we identified V_1_ domain interaction partners by co-IP and LC–MS. V_1_ and V_o_ domain subunits were highly enriched in HA-V1B2 immunoprecipitates, confirming the specificity of the co-IP (Fig. 3A; Dataset EV3). V1B2 immunoprecipitates were also enriched in subunits of the Ragulator complex (Lamtor1–4), which associates with lysosomal V-ATPases (Zoncu et al, 2011). Notably, V1B2 co-IPs robustly recovered the different mRAVE subunits, Dmxl1, Wdr7 and Rogdi. Immunoblotting confirmed that HA-V1B2 co-precipitated V5-tagged Wdr7 and V_o_ domains (Fig. 3B). Thus, the V_1_ domain interacts with both the V_o_ domain and mRAVE, consistent with previous results (Lee et al, 2025; Nardone et al, 2025).

**Figure EV4:**
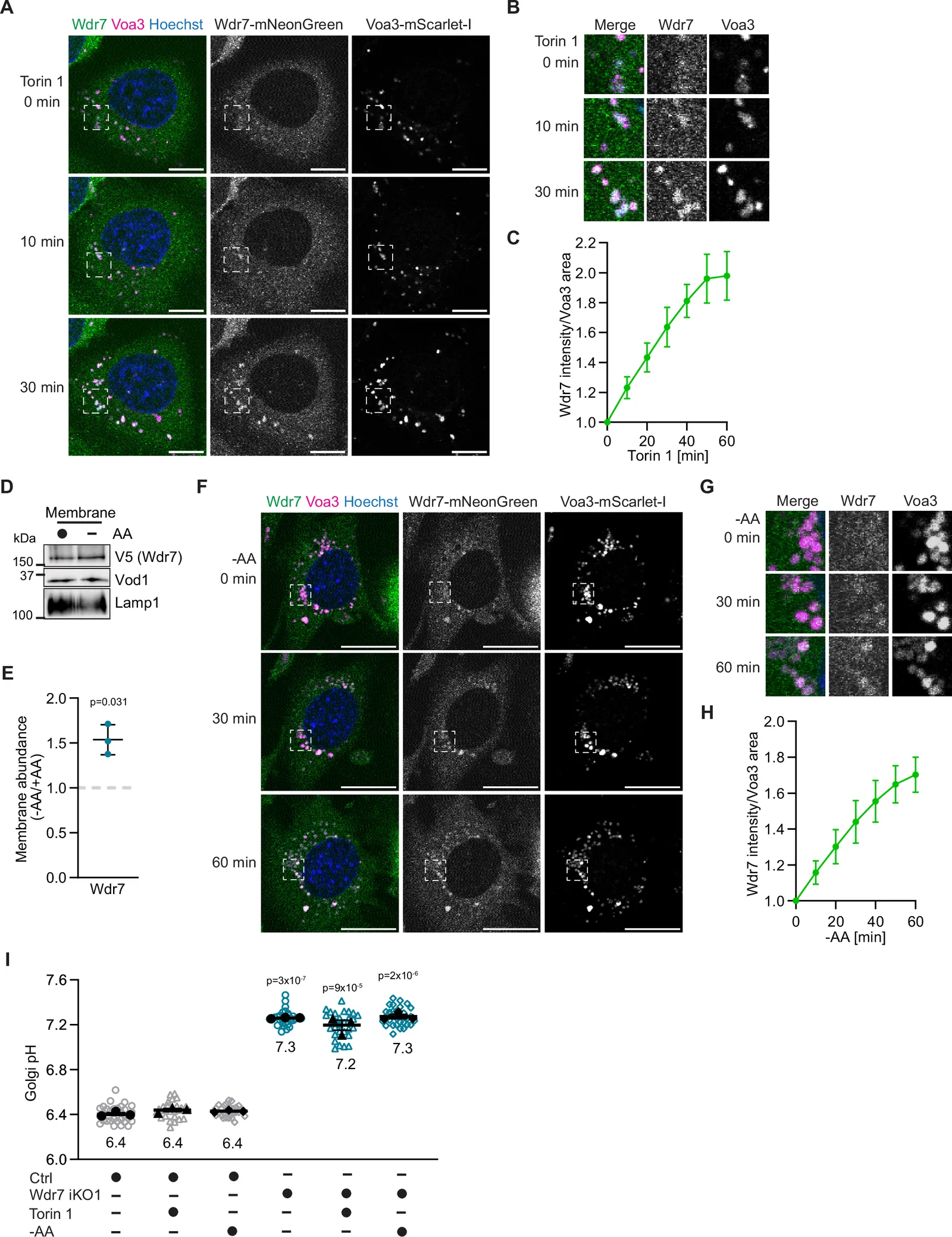
mRAVE accumulates at lysosomes in response to mTORC1 inactivation (related to Figs. 3 and 4). (**A**) Subcellular localization of Wdr7-mNeonGreen and Voa3-mScarlet-I in U2OS cells after addition of torin 1 [400 nM], analyzed by live-cell imaging. (**B**) Magnification of areas highlighted in (**A**). (**C**) Change of Wdr7-mNeonGreen intensity in Voa3-mScarlet-I-containing lysosomes, quantified by live-cell imaging as in (**A**). (**D**) V5-Wdr7 abundance in membrane fractions of dextran-loaded lysosomes from MEFs after 1 h ± amino acid starvation, analyzed by immunoblotting. (**E**) Quantification of V5-Wdr7 abundance in immunoblots from membrane fractions as in (**D**). (**F**) Subcellular localization of Wdr7-mNeonGreen and Voa3-mScarlet-I in MEFs after the onset of amino acid starvation, analyzed by live-cell imaging. (**G**) Magnification of areas highlighted in (**F**). (**H**) Change of Wdr7-mNeonGreen intensity in Voa3-mScarlet-I-containing lysosomes, quantified by live-cell imaging as in (**F**). (**I**) Golgi pH in Wdr7 iKO MEFs after 1 h ± torin 1 [400 nM] or amino acid starvation, quantified by ratiometric imaging of MGAT-SEP-mRuby. (**C**,** H**) Data are mean ± SD (≥15,000 organelles in ≥21 fields of view with a total of ≥180 cells). (**E**) Data are mean ± SD (*n* = 3 independent experiments). (**I**) Data are replicate mean ± SEM (closed circles; *n* = 3 independent experiments) and fields of view (open circles; ≥10 per replicate). *p* values were calculated using a two-tailed unpaired *t*-test. Scale bars, 10 µm.

mTORC1 inactivation triggers the recruitment of V_1_ domains to lysosomal V_o_ domains to assemble active proton pumps (Ratto et al, 2022). To investigate a potential role of mRAVE in this process, we treated cells with the mTOR kinase inhibitor torin 1 and examined the localization of the assembly factor. Torin 1 treatment increased the abundance of V5-Wdr7 in organelle fractions enriched for dextran-weighted lysosomes (Fig. 3C, D). Consistently, live-cell imaging showed that Wdr7-mNeonGreen was predominantly cytosolic under basal conditions but rapidly accumulated at Voa3-mScarlet-I-containing lysosomes upon torin 1 treatment (Fig. 3E–G). Similar lysosomal accumulation was observed for Dmxl1-mNeonGreen in MEFs (Fig. 3H–J) and for Wdr7-mNeonGreen in U2OS cells (Fig. EV4A–C). Physiological mTORC1 inactivation by amino acid starvation likewise led to Wdr7 enrichment in organelle fractions and recruitment to Voa3-containing lysosomes (Fig. EV4D–H). Wdr7-mNeonGreen and V1B2-mScarlet-I co-accumulated at lysosomes with similar kinetics (Fig. 3K–M), suggesting coordinated recruitment of mRAVE and V_1_ domains to the organelle upon mTORC1 inactivation.

**Figure 4:**
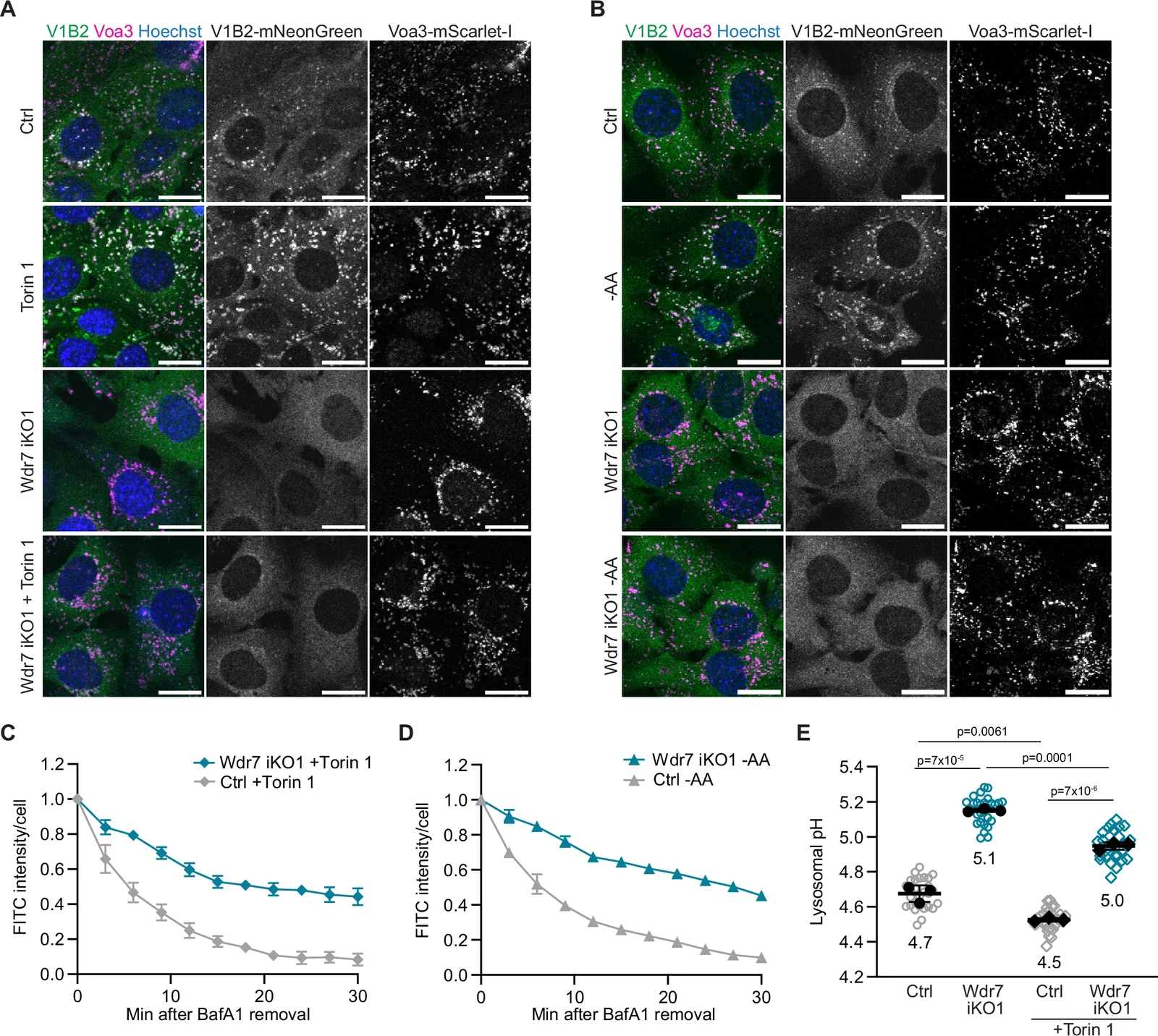
mRAVE is required for enhanced V-ATPase assembly upon mTORC1 inactivation. (**A**,** B**) Subcellular localization of V1B2-mNeonGreen and Voa3-mScarlet-I in Wdr7 iKO MEFs after 1 h ±  torin 1 [400 nM] (**A**) or 1 h ± amino acid starvation (**B**), analyzed by live-cell imaging. (**C**, **D**) Fluorescence quenching of lysosomally loaded FITC-dextran in Wdr7 iKO MEFs after 1 h bafilomycin A1 [20 nM] and torin 1 [400 nM] (**C**) or amino acid starvation (**D**) and subsequent bafilomycin A1 removal. (**E**) Lysosomal pH in Wdr7 iKO cells after 1 h ± torin 1 [400 nM], quantified by ratiometric imaging of 10 kDa Dextran Oregon Green and Alexa Fluor 647. (**C**,** D**) Data are replicate mean ± SEM (*n* = 3 independent experiments). (**E**) Data are replicate mean ± SEM (closed circles; *n* = 3 independent experiments) and fields of view (open circles; ≥9 per replicate). *p* values were calculated using a two-tailed unpaired *t*-test. Scale bars, 20 µm. Source data are available online for this figure.

Next, we examined whether mRAVE is required for mTORC1-regulated V-ATPase assembly. In control cells, torin 1 treatment or amino acid starvation triggered recruitment of V1B2 to Voa3-containing lysosomes, indicating enhanced V-ATPase assembly (Fig. 4A,B). In Wdr7-deficient cells, however, V1B2 did not accumulate at lysosomes upon mTORC1 inactivation but remained cytosolic. Consequently, the rate and extent of lysosomal acidification were strongly reduced in Wdr7-deficient cells subjected to mTORC1 inhibition as measured by FITC quenching (Fig. 4C,D), and lysosomal pH was markedly increased (Fig. 4E). Thus, mRAVE is required for enhanced lysosomal V-ATPase assembly and luminal acidification in response to mTORC1 inactivation. In contrast, Golgi pH was not affected by torin 1 or amino acid starvation, suggesting that V-ATPase assembly at the Golgi depends on mRAVE but is not regulated by mTORC1 (Fig. EV4I).

### mRAVE deficiency causes accumulation of dysfunctional lysosomes

**Figure 5:**
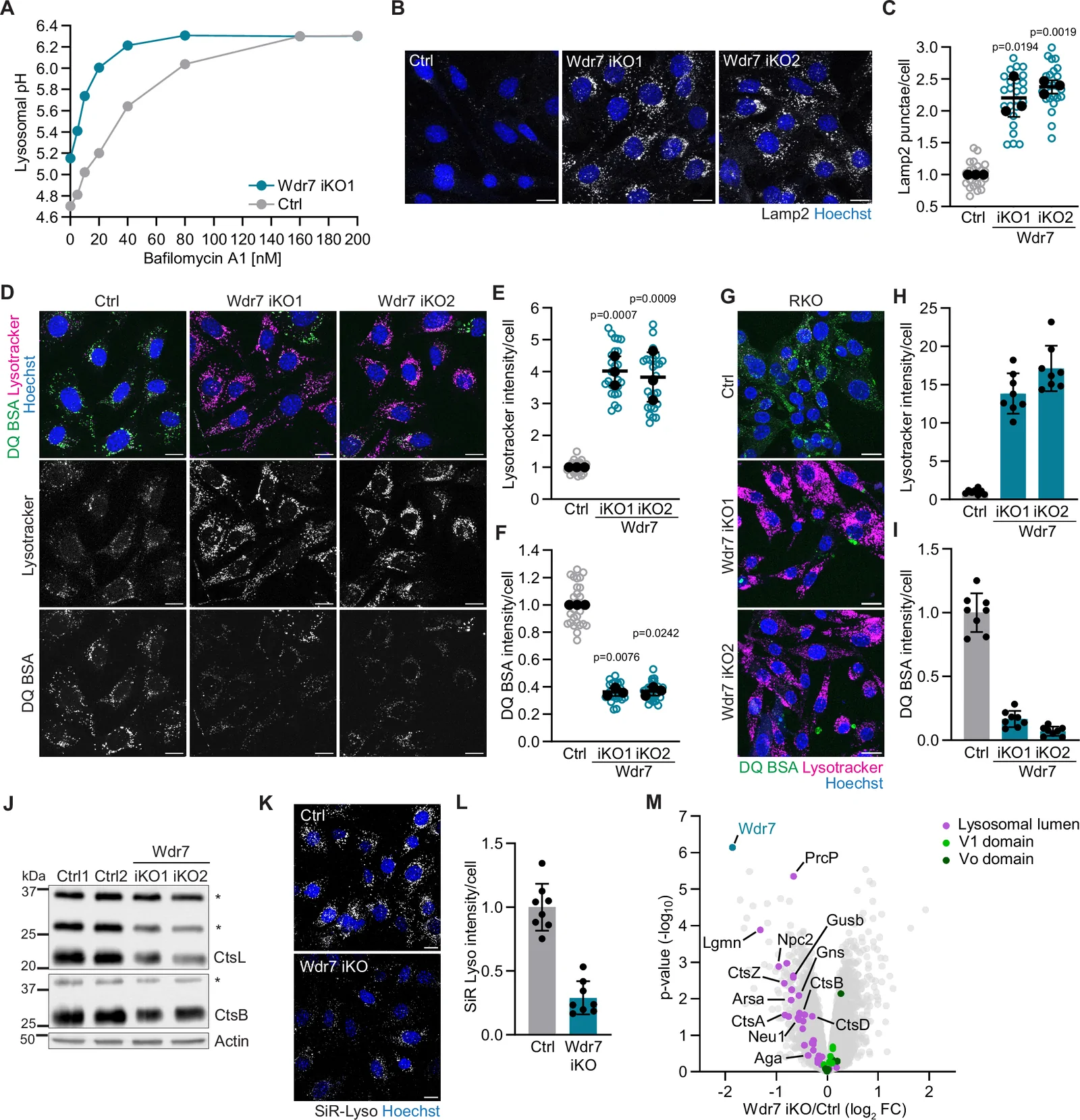
Loss of mRAVE causes accumulation of lysosomes with impaired catabolic activity. (**A**) Lysosomal pH in Wdr7 iKO MEFs after 1.5 h bafilomycin A1 at indicated concentrations, quantified by ratiometric imaging of 10 kDa Dextran Oregon Green and Alexa Fluor 647. (**B**) Lysosome accumulation in Wdr7 iKO MEFs, analyzed by Lamp2 immunofluorescence. (**C**) Quantification of Lamp2 in Wdr7 iKO MEFs as in (**B**). (**D**) Lysotracker accumulation and DQ BSA degradation in Wdr7 iKO MEFs, analyzed by live-cell imaging. (**E**, **F**) Quantification of Lysotracker (**E**) and DQ BSA (**F**) in Wdr7 iKO MEFs as in (**D**). (**G**) Lysotracker accumulation and DQ BSA degradation in Wdr7 iKO RKO cells, analyzed by live-cell imaging. (**H**,** I**) Quantification of Lysotracker (**H**) and DQ BSA (**I**) in Wdr7 iKO RKO cells as in (**G**). (**J**) Cathepsin abundance in Wdr7 iKO MEFs, analyzed by immunoblotting. Asterisk denotes immature proenzymes. (**K**) Active cathepsin D abundance in Wdr7 iKO MEFs, analyzed by SiR-Lysosome imaging. (**L**) Quantification of SiR-Lysosome fluorescence of cells shown in (**K**). (**M**) Changes in the proteome of Wdr7 iKO MEFs, quantified by label-free LC–MS. (**A**) Data are replicate mean (*n* = 3 independent replicates; ≥8 fields of view per replicate). (**C**,** E**,** F**) Data are replicate mean ± SEM (closed circles; *n* = 3 independent replicates) and fields of view (open circles; ≥8 per replicate). (**H**,** I**,** L**) Data are mean ± SD and fields of view (*n* = 8). (**M**) *n* = 4 independent experiments. *p* values were calculated using a two-tailed unpaired *t*-test with Welch correction. Scale bars, 20 µm. Source data are available online for this figure.

**Figure EV5:**
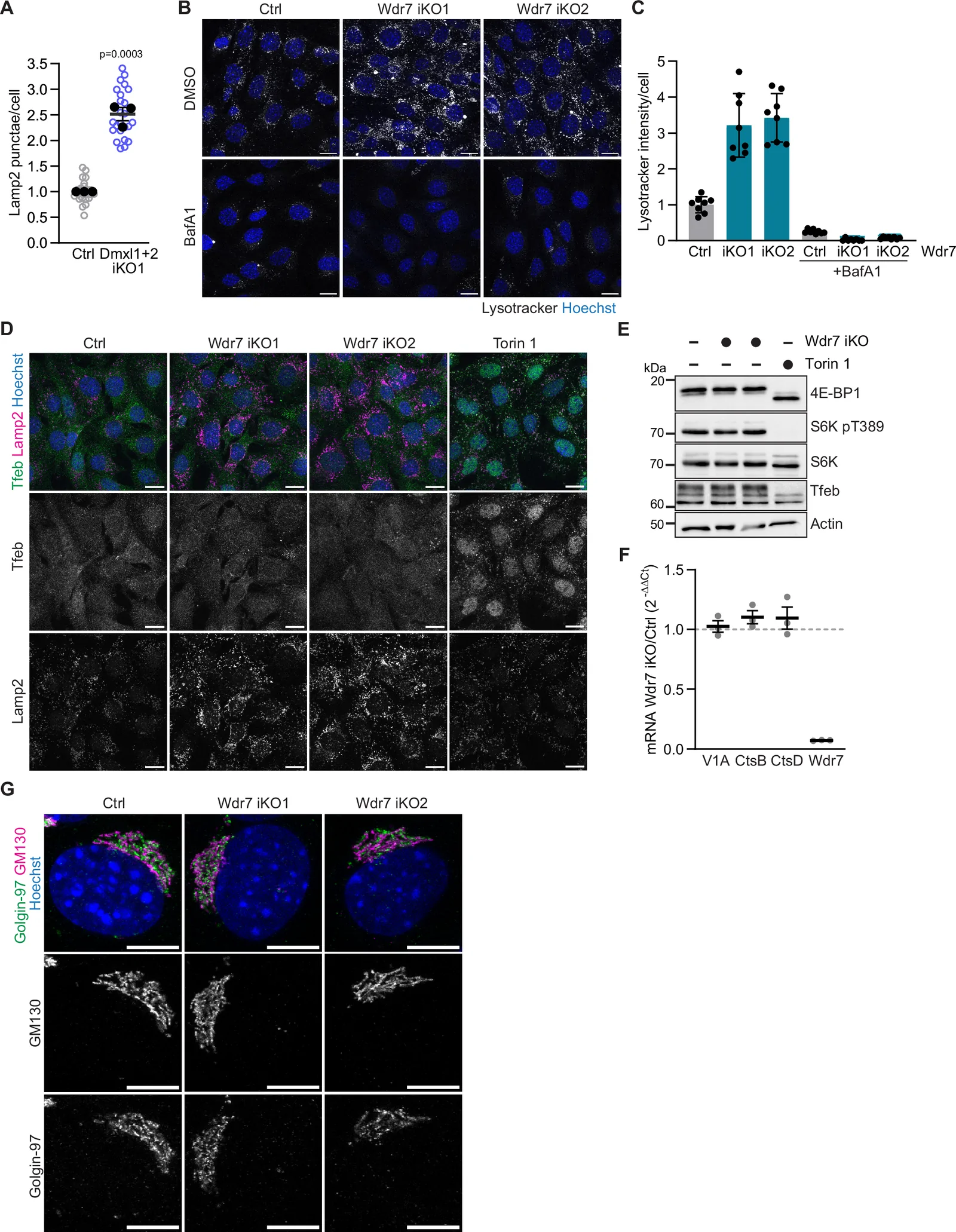
Accumulation of lysosomes in mRAVE-deficient cells is not accompanied by nuclear translocation of Tfeb or changes in Golgi morphology (related to Fig. 5). (**A**) Quantification of lysosome accumulation in Dmxl1 + Dmxl2 iKO MEFs, assessed by Lamp2 immunofluorescence. (**B**) Lysotracker accumulation in Wdr7 iKO MEFs after 1 h ± bafilomycin A1 [20 nM], analyzed by live-cell imaging. (**C**) Quantification of lysotracker in Wdr7 iKO MEFs as in (**B**). (**D**) Subcellular localization of Tfeb in MEFs ± Wdr7 iKO or 1 h torin 1 [400 nM], analyzed by immunofluorescence. (**E**) mTORC1 signaling activity in MEFs ± Wdr7 iKO or 1 h torin 1 [400 nM], analyzed by phosphorylation of indicated downstream targets. (**F**) Relative mRNA abundance of indicated genes in Wdr7 iKO MEFs, analyzed by RT-qPCR. (**G**) Golgi morphology in Wdr7 iKO MEFs, analyzed by immunofluorescence against the cis-Golgi marker GM130 and the trans-Golgi marker Golgin-97. (**A**) Data are replicate mean ± SEM (closed circles; *n* = 3 independent replicates) and fields of view (open circles; ≥8 per replicate). (**C**) Data are mean ± SD and fields of view (*n* = 8). (**F**) Data are replicate mean ± SEM (*n* = 3 independent replicates). *p* values were calculated using a two-tailed unpaired *t*-test with Welch correction. Scale bars, 20 µm (**B**, **D**) or 5 μm (**G**).

Because lysosomes in mRAVE-deficient cells were partially acidic—despite the strong reduction in assembled V-ATPases—we examined whether the remaining acidification depends on residual V-ATPase activity. Treating Wdr7-deficient cells with bafilomycin A1 elevated lysosomal pH, indicating that some luminal acidification was sustained by V-ATPases (Fig. 5A). However, lysosomes were de-acidified at substantially lower inhibitor concentrations than in control cells, consistent with a reduced pool of active proton pumps. To assess the functional consequences of decreased acidification, we examined lysosome abundance by staining for Lamp2, a ubiquitous lysosomal membrane protein (Chen et al, 1985). Cells deficient for Wdr7 or for both Dmxl1 and Dmxl2 displayed a marked accumulation of Lamp2-positive organelles (Figs. 5B,C and EV5A). This change coincided with an increase in lysotracker red, a fluorescent dye whose accumulation in cells reflects both lysosomal abundance and acidity (Fig. 5D,E). Bafilomycin A1 treatment abolished lysotracker signal (Fig. EV5B,C), suggesting that without mRAVE, lysosomes remained sufficiently acidic to retain the dye. The expansion of the lysosomal compartment in Wdr7-deficient cells was not accompanied by increased activity of Tfeb, a transcription factor that promotes lysosomal biogenesis (Fig. EV5D–F). In contrast to lysosomes, Golgi morphology was not obviously altered, despite neutralization of the Golgi lumen (Fig. EV5G). Together, these results show that loss of mRAVE leads to accumulation of lysosomes with diminished acidity.

**Figure EV6:**
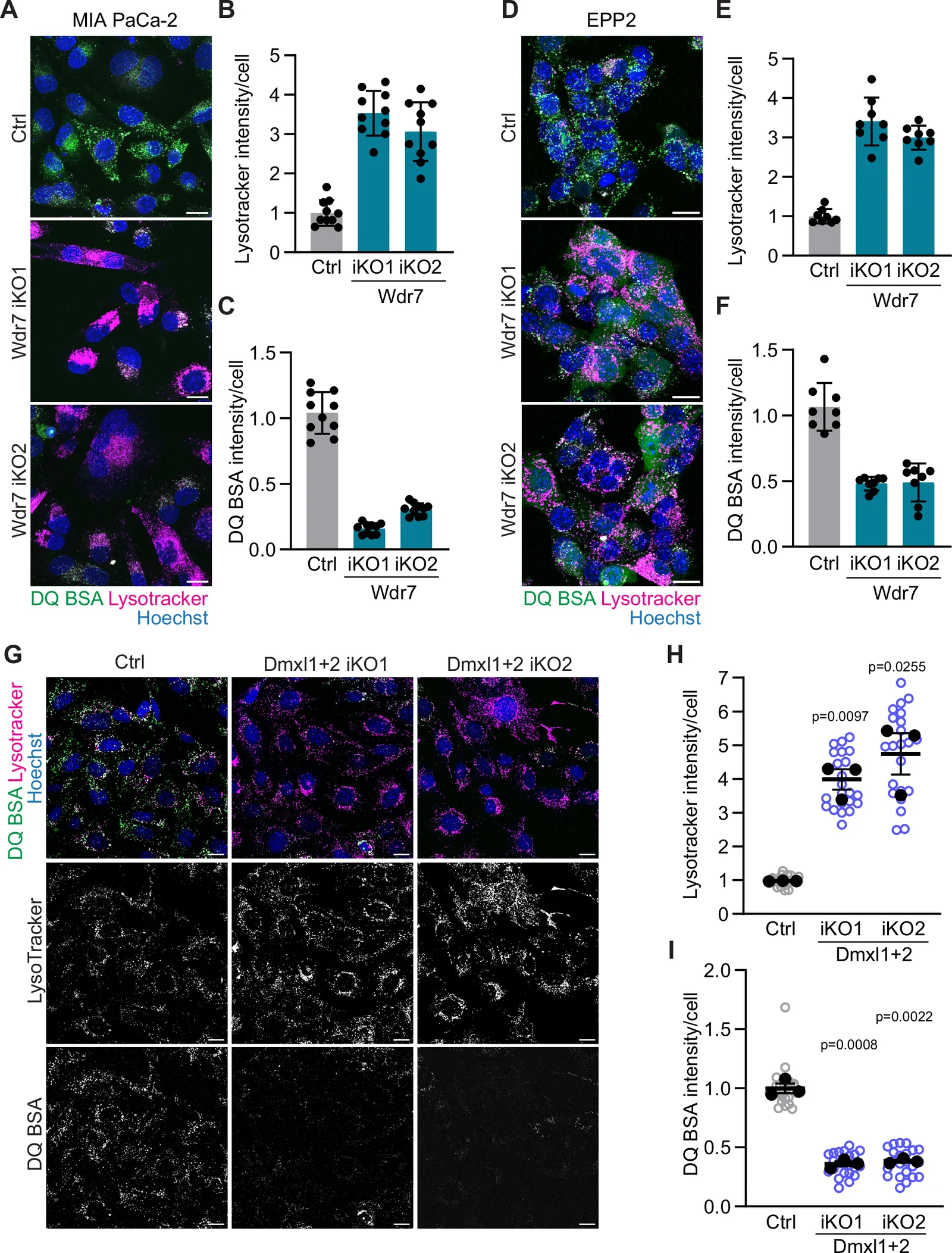
Loss of Wdr7 suppresses lysosomal catabolism (related to Fig. 5). (**A**) Lysotracker accumulation and DQ BSA degradation in Wdr7 iKO MIA PaCa-2 cells, analyzed by live-cell imaging. (**B**, **C**) Quantification of lysotracker (**B**) and DQ BSA (**C**) in Wdr7 iKO MIA PaCa-2 cells, as shown in (**A**). (**D**) Lysotracker accumulation and DQ BSA degradation in Wdr7 iKO EPP2 cells, analyzed by live-cell imaging. (**E**, **F**) Quantification of lysotracker (**E**) and DQ BSA (**F**) in Wdr7 iKO EPP2 cells, as shown in (**D**). (**G**) Lysotracker accumulation and DQ BSA degradation in Dmxl1 +  Dmxl2 iKO MEFs, analyzed by live-cell imaging. (**H**, **I**) Quantification of lysotracker (**H**) and DQ BSA (**I**) in Dmxl1 + Dmxl2 iKO MEFs, as shown in (**G**). (**B**,** C**,** E**,** F**) Data were mean ± SD and fields of view (*n* = 8–10). (**H**,** I**) Data are replicate mean ± SEM (closed circles; *n* = 3 independent replicates) and fields of view (open circles; ≥8 per replicate). *p* values were calculated using a two-tailed unpaired *t*-test with Welch correction. Scale bars, 20 µm.

**Figure EV7:**
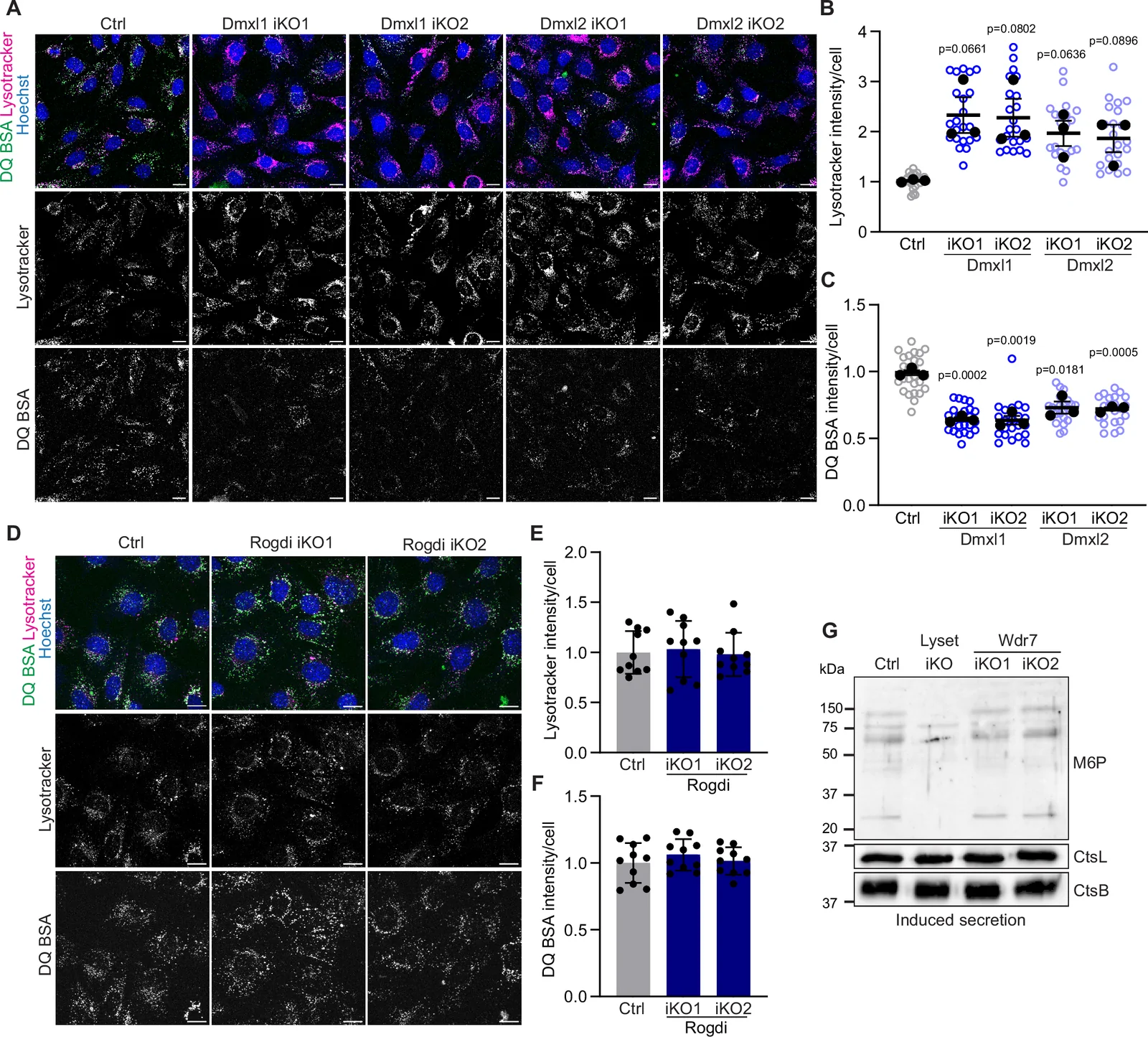
Lysosomal phenotypes in Dmxl1, Dmxl2 or Rogdi iKO cells (related to Fig. 5). (**A**) Lysotracker accumulation and DQ BSA degradation in Dmxl1 iKO and Dmxl2 iKO MEFs, analyzed by live-cell imaging. (**B**, **C**) Quantification of lysotracker (**B**) and DQ BSA (**C**) in Dmxl1 iKO and Dmxl2 iKO MEFs, as shown in (**A**). (**D**) Lysotracker accumulation and DQ BSA degradation in Rogdi iKO MEFs, analyzed by live-cell imaging. (**E**, **F**) Quantification of lysotracker (**E**) and DQ BSA (**F**) in Rogdi iKO MEFs, as shown in (**D**). (**G**) M6P modification of newly synthesized proteins under induced secretion conditions (10 mM NH₄Cl, 24 h) in Wdr7 iKO and Lyset iKO MEFs, analyzed by immunoblotting. (**B**,** C**) Data are replicate mean ± SEM (closed circles; *n* = 3 independent replicates) and fields of view (open circles; ≥7 per replicate). (**E**,** F**) Data are mean ± SD and fields of view (*n* = 10). *p* values were calculated using a two-tailed unpaired *t*-test with Welch correction. Scale bars, 20 µm.

To examine the impact of mRAVE on the catabolic activity of lysosomes, we incubated cells with DQ BSA, an albumin probe whose fluorescence becomes dequenched upon degradation in lysosomes (Palm et al, 2015). Loss of Wdr7 led to a substantial decrease in DQ BSA degradation in MEFs (Fig. 5D,F), as well as in several mouse and human cancer cell lines (Figs. 5G,I and EV6A,C,D,F). A comparable decrease in DQ BSA degradation was observed in MEFs deficient for both Dmxl1 and Dmxl2 (Fig. EV6G,I). In each of these cell lines, loss of mRAVE led to a strong accumulation of lysotracker-positive organelles (Figs. 5D,E,G,H and EV6A,B,D,E,G,H). We also examined the individual impact of Dmxl1, Dmxl2 and Rogdi. Loss of Dmxl1 or Dmxl2 moderately decreased DQ BSA degradation and increased lysotracker signal (Fig. EV7A–C), while loss of Rogdi had no effect (Fig. EV7D–F), mirroring the impact of these genes on cell proliferation.

To further characterize the defects in lysosomal catabolism resulting from loss of mRAVE, we examined the abundance of lysosomal enzymes. Wdr7-deficient MEFs showed a marked reduction in the mature forms of cathepsins B and L, two ubiquitous lysosomal proteases (Fig. 5J) (Turk et al, 2012). Consistently, SiR-lysosome, a fluorescent probe that binds to active cathepsin D, was decreased (Fig. 5K,L) (Lukinavičius et al, 2016). To further characterize the phenotypes of mRAVE-deficient cells, we quantified cellular proteins in Wdr7-deficient MEFs by LC–MS and LFQ. Loss of Wdr7 led to an overall decrease in the abundance of lysosomal enzymes and other luminal lysosomal proteins (Fig. 5M; Dataset EV4). This decrease in lysosomal enzymes and lysosomal proteolytic activity in the absence of mRAVE, despite an expanded lysosomal compartment, indicates a pronounced defect in lysosomal catabolic activity.

### mRAVE deficiency results in lysosomal exocytosis

**Figure 6:**
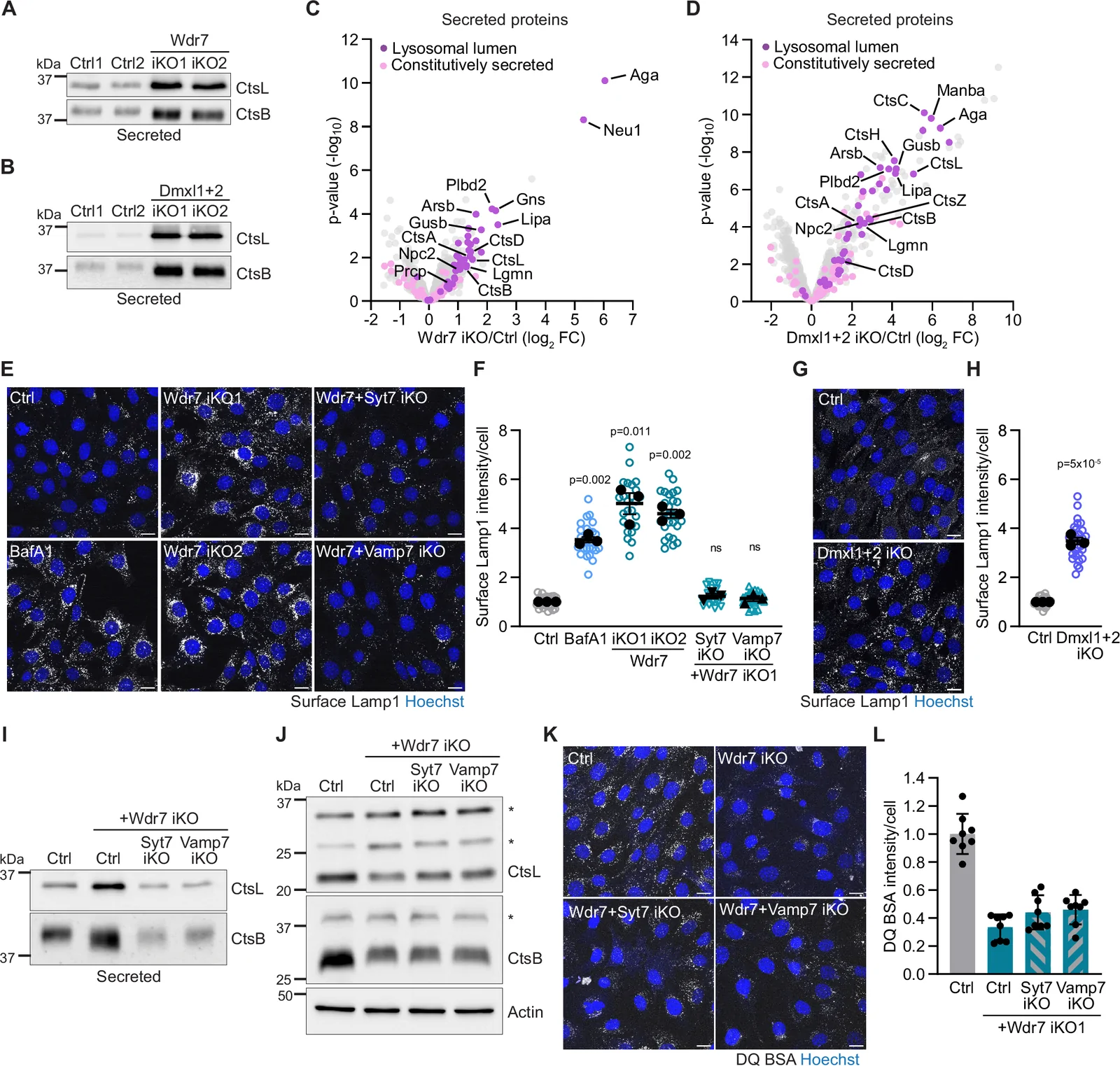
Loss of mRAVE results in lysosomal exocytosis. (**A**, **B**) Immature cathepsin abundance in supernatants from Wdr7 (**A**) and Dmxl1 + Dmxl2 (**B**) iKO MEFs, analyzed by immunoblotting. (**C**,** D**) Changes in luminal lysosomal proteins and constitutively secreted proteins in supernatants from Wdr7 (**C**) and Dmxl1 + Dmxl2 (**D**) iKO MEFs, quantified by label-free LC–MS. (**E**) Lysosomal exocytosis in MEFs with Wdr7 iKO ± Syt7 or Vamp7 iKO or 2 h bafilomycin A1 [1 µM], analyzed by immunofluorescence of cell-surface Lamp1. (**F**) Quantification of cell-surface Lamp1 in cells as in (**E**). (**G**) Lysosomal exocytosis in MEFs with Dmxl1 +  Dmxl2 iKO, analyzed by immunofluorescence of cell-surface Lamp1. (**H**) Quantification of cell-surface Lamp1 in cells as in (**G**). (**I**) Immature cathepsin secretion in Wdr7 iKO MEFs ± Syt7 iKO or Vamp7 iKO, analyzed by immunoblotting. (**J**) Cathepsin abundance in Wdr7 iKO MEFs ± Syt7 iKO or Vamp7 iKO, analyzed by immunoblotting. Asterisk denotes immature proenzyme. (**K**) DQ BSA degradation in Wdr7 iKO MEFs ± Syt7 iKO or Vamp7 iKO, analyzed by live-cell imaging. (**L**) Quantification of DQ BSA in cells shown in (**K**). (**C**, **D**) *n* = 5 independent experiments. (**F**,** H**) Data are replicate mean ± SEM (closed circles; *n* = 3 independent experiments) and fields of view (open circles; ≥8 per replicate). (**L**) Data are mean ± SD and fields of view (*n* = 8). *p* values were calculated using a two-tailed unpaired *t*-test with Welch correction. Scale bars, 20 µm. Source data are available online for this figure.

Lysosomal enzymes are synthesized as inactive precursors and proteolytically processed into their mature forms after reaching the lysosome (Braulke et al, 2024). We noted that mature cathepsins were reduced in mRAVE-deficient cells, whereas their mRNA and immature proenzyme levels appeared normal (Figs. 5J and EV5F), suggesting perturbed enzyme trafficking. To assess whether enzymes were missecreted, we analyzed cellular supernatants by immunoblotting. Immature pro-cathepsins B and L were strongly increased in supernatants from cells deficient for Wdr7 or for both Dmxl1 and Dmxl2 (Fig. 6A,B). To more broadly examine lysosomal enzyme secretion, we quantified proteins in cellular supernatants using LC–MS and LFQ. Lysosomal enzymes were overall increased in supernatants from cells deficient for Wdr7 or for both Dmxl1 and Dmxl2, with little change in constitutively secreted proteins (Fig. 6C,D; Dataset EV5). Thus, loss of mRAVE leads to increased release of lysosomal enzymes into the extracellular space.

Lysosomal enzyme secretion can result from disruption of mannose 6-phosphate (M6P) modification, the lysosomal trafficking signal which is added to newly synthesized enzymes in the Golgi (Braulke et al, 2024). Upon inducing enzyme secretion with ammonium chloride, however, M6P-modified proteins were detected at comparable levels in supernatants from Wdr7-deficient cells and controls (Fig. EV7G). In contrast, they were barely detectable in supernatants from cells deficient for LYSET, a core M6P pathway component (Pechincha et al, 2022; Richards et al, 2022). Thus, M6P modification of lysosomal enzymes is not perturbed in mRAVE-deficient cells, despite elevated Golgi pH.

An alternative route for lysosomal enzyme secretion is lysosomal exocytosis. During this process, which can be triggered by impaired organellar acidification, lysosomes fuse with the plasma membrane, resulting in cell surface exposure of lysosomal membrane proteins and release of luminal contents (Miao et al, 2015; Tancini et al, 2020). To determine whether exocytosis accounts for lysosomal enzyme secretion in mRAVE-deficient cells, we performed cell-surface staining for the luminal domain of the lysosomal transmembrane protein Lamp1 (Reddy et al, 2001). Cells deficient for Wdr7 or for both Dmxl1 and Dmxl2 displayed a strong increase in Lamp1 at the cell surface, similar to the effect of V-ATPase inhibition with bafilomycin A1 (Fig. 6E–H). To directly test the involvement of lysosomal exocytosis, we genetically ablated Syt7 or Vamp7, which are required for fusion of lysosomes with the plasma membrane (Rao et al, 2004). Co-depletion of Wdr7 together with Syt7 or Vamp7 suppressed the cell-surface localization of Lamp1 (Fig. 6E,F). Consistently, depleting Syt7 or Vamp7 in Wdr7-deficient cells abrogated the increased secretion of cathepsins B and L (Fig. 6I). Thus, secretion of immature lysosomal enzymes in mRAVE-deficient cells occurs through the lysosomal exocytosis pathway. Notably, suppressing lysosomal exocytosis in Wdr7-deficient cells, did not restore intracellular levels of mature cathepsins (Fig. 6J) or rescue the defect in DQ BSA degradation (Fig. 6K,L). These results indicate that lysosomal exocytosis and the resulting secretion of luminal enzymes are a consequence of the lysosomal dysfunction which arises in the absence of mRAVE.

### mRAVE couples V-ATPase assembly to mTORC1-regulated control of lysosomal catabolism

**Figure EV8:**
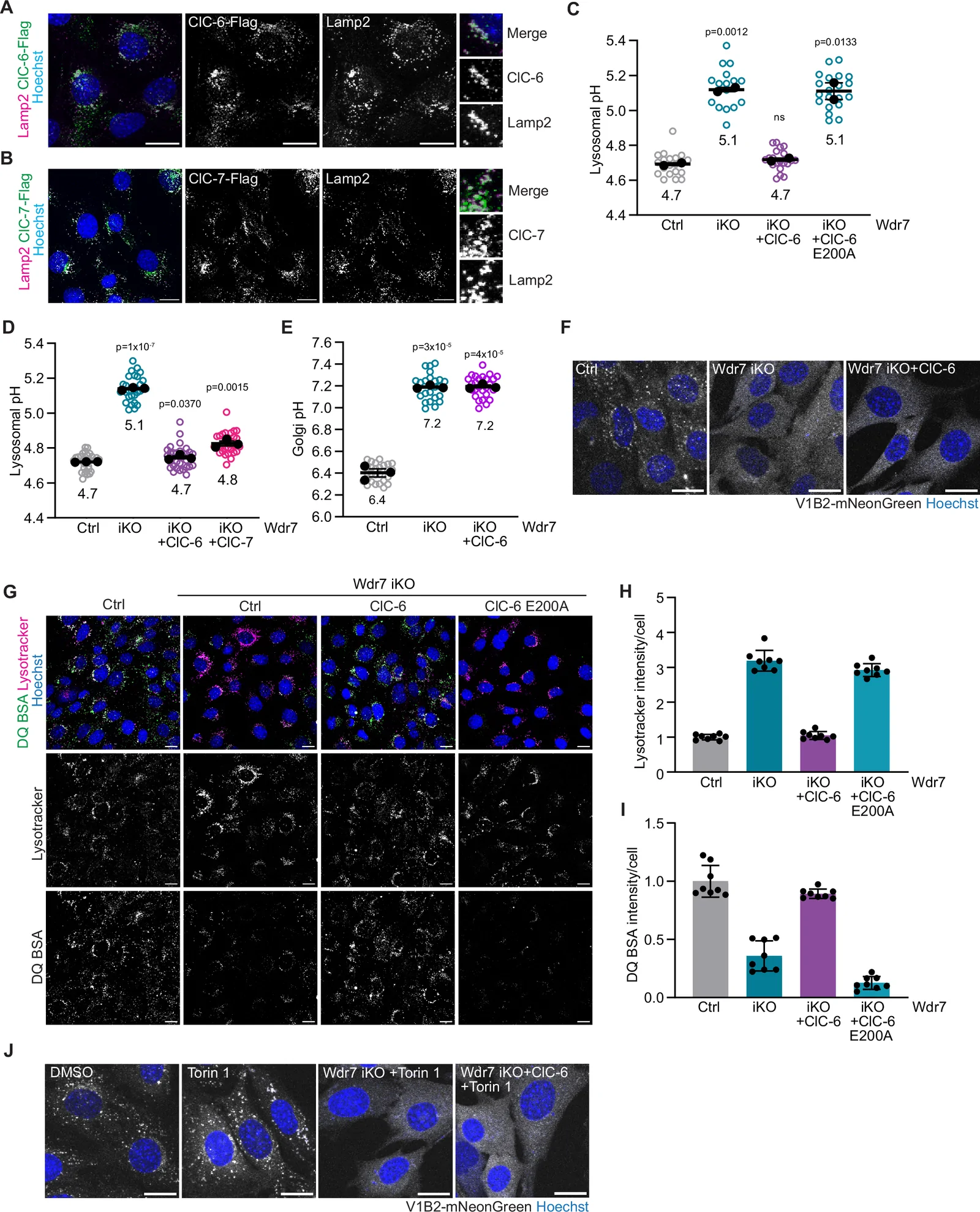
The Cl^-^/H^+^ antiporters ClC-6 and ClC-7 restore lysosomal defects of Wdr7 iKO MEFs (related to Fig. 7). (**A**,** B**) Colocalization of ClC-6-Flag (**A**) or ClC-7-Flag (**B**) with endogenous Lamp2 in MEFs, analyzed by immunofluorescence. (**C**) Lysosomal pH in Wdr7 iKO MEFs ± expression of ClC-6 or mutant ClC-6^E200A^, which is deficient for proton transport but retains chloride conductance (Neagoe et al, 2010), quantified by ratiometric imaging of 10 kDa Dextran Oregon Green and Alexa Fluor 647. (**D**) Lysosomal pH in Wdr7 iKO MEFs ± ClC-6 or ClC-7 expression, quantified by ratiometric imaging of 10 kDa Dextran Oregon Green and Alexa Fluor 647. (**E**) Golgi pH in Wdr7 iKO MEFs ± ClC-6 expression, quantified by ratiometric imaging of MGAT-SEP-mRuby. (**F**) Subcellular localization of V1B2-mNeonGreen in Wdr7 iKO MEFs ± ClC-6 expression, analyzed by live-cell imaging. (**G**) Lysotracker accumulation and DQ BSA degradation in Wdr7 iKO MEFs ± expression of ClC-6 or proton transport-deficient ClC-6^E200A^, analyzed by live-cell imaging. (**H**, **I**) Quantification of lysotracker (**H**) and DQ BSA (**I**) in Wdr7 iKO MEFs ± expression of ClC-6 or proton transport-deficient ClC-6^E200A^ as shown in (**G**). (**J**) Subcellular localization of V1B2-mNeonGreen in Wdr7 iKO MEFs after 1 h ± torin 1 [400 nM], analyzed by live-cell imaging. (**C**–**E**) Data are replicate mean ± SEM (closed circles; *n* = 2–3 independent experiments) and fields of view (open circles; ≥8 per replicate). (**H**,** I**) Data are mean ± SD and fields of view (*n* = 8). *p* values were calculated using a two-tailed unpaired *t*-test. Scale bars, 20 µm.

**Figure 7:**
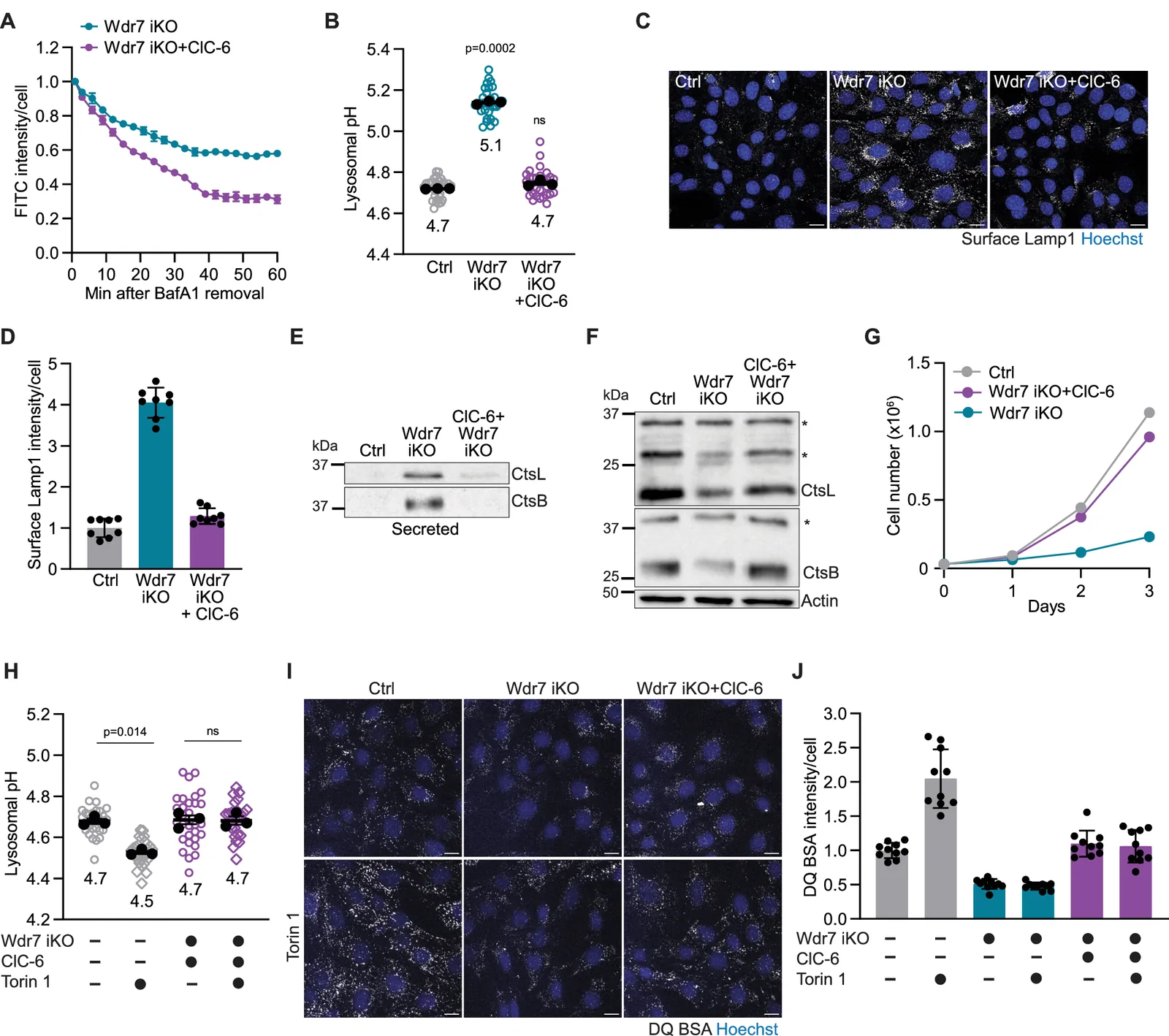
ClC-6 expression restores basal but not mTORC1-regulated lysosomal catabolism in the absence of mRAVE. (**A**) Fluorescence quenching of lysosomal FITC-dextran in Wdr7 iKO MEFs ± ClC-6 expression after 1 h bafilomycin A1 [20 nM] and subsequent bafilomycin A1 removal. (**B**) Lysosomal pH in Wdr7 iKO MEFs ± ClC-6 expression, quantified by ratiometric imaging of 10 kDa Dextran Oregon Green and Alexa Fluor 647. (**C**) Lysosomal exocytosis in Wdr7 iKO MEFs ± ClC-6 expression, analyzed by immunofluorescence of cell-surface Lamp1. (**D**) Quantification of cell-surface Lamp1 in cells shown in (**C**). (**E**) Immature cathepsin abundance in supernatants from Wdr7 iKO MEFs ± ClC-6 expression, analyzed by immunoblotting. (**F**) Cathepsin abundance in Wdr7 iKO MEFs ± ClC-6 expression, analyzed by immunoblotting. Asterisk denotes immature proenzymes. (**G**) Proliferation of Wdr7 iKO MEFs ± ClC-6 expression. (**H**) Lysosomal pH in Wdr7 iKO MEFs ± ClC-6 expression after 1 h ±  torin 1 [400 nM], quantified by ratiometric imaging of 10 kDa Dextran Oregon Green and Alexa Fluor 647. (**I**) DQ BSA degradation in Wdr7 iKO MEFs ± ClC-6 expression after 1 h ± torin 1 [400 nM], analyzed by live-cell imaging. (**J**) Quantification of DQ BSA fluorescence in cells shown in (**I**). (**A**) Data are replicate mean ± SEM (*n* = 3 independent experiments). (**B**,** H**) Data are replicate mean ± SEM (closed circles; *n* = 3 independent experiments) and fields of view (open circles; ≥10 per replicate). (**D**,** J**) Data are mean ± SD and fields of view (*n* = 8–10). (**G**) Data are mean ± SD (*n* = 3 technical replicates). *p* values were calculated using a two-tailed unpaired *t*-test. Scale bar = 20 µm. Source data are available online for this figure.

The above results establish a central role for mRAVE in the assembly of V-ATPase complexes across multiple organelles. To determine which phenotypes depend specifically on the acidification of lysosomes, we sought to restore lysosomal pH independently of mRAVE. We ectopically expressed H^+^/Cl^-^ antiporters, ClC-6 and ClC-7, which localize to endolysosomal compartments (Fig. EV8A,B) and facilitate V-ATPase-dependent luminal acidification (Jentsch and Pusch, 2018; Settembre and Perera, 2024). Expression of ClC-6 in Wdr7-deficient cells increased the rate of lysosomal acidification (Fig. 7A) and restored lysosomal pH to control levels in a proton transport-dependent manner (Figs. 7B and EV8C). ClC-7 had a slightly weaker effect (Fig. EV8D). We therefore utilized ClC-6, whose expression in Wdr7-deficient cells did not rescue Golgi pH (Fig. EV8E) or V-ATPase assembly (Fig. EV8F). Thus, ClC-6 expression provides a mean to functionally uncouple lysosomal acidification from mRAVE-dependent V-ATPase assembly.

Restoring lysosomal acidification corrected multiple defects caused by loss of mRAVE. Expression of ClC-6 in Wdr7-deficient cells suppressed Lamp1 cell-surface localization (Fig. 7C, D) and prevented secretion of cathepsins B and L (Fig. 7E). Intracellularly, this was accompanied by restoration of mature cathepsin levels, lysosomal proteolytic activity and lysotracker accumulation (Figs. 7F and EV8G–I). Whereas loss of Wdr7 severely compromised cell proliferation, expression of ClC-6 restored proliferation to rates comparable to controls (Fig. 7G). These findings indicate that sustaining lysosomal acidification is the essential function of mRAVE in proliferating cells. Restoring basal acidification in the absence of mRAVE, however, was not sufficient to support adaptive increases in lysosomal activity. Torin 1 treatment enhanced V-ATPase assembly, lysosomal acidification and DQ BSA degradation in control cells, but failed to do so in Wdr7-deficient cells—even when basal lysosomal acidification was restored via ClC-6 (Figs. 7H–J and EV8J). Thus, mRAVE-mediated V-ATPase assembly is critical to enhance the catabolic activity of lysosomes in response to mTORC1 inactivation.

## Discussion

**Figure 8:**
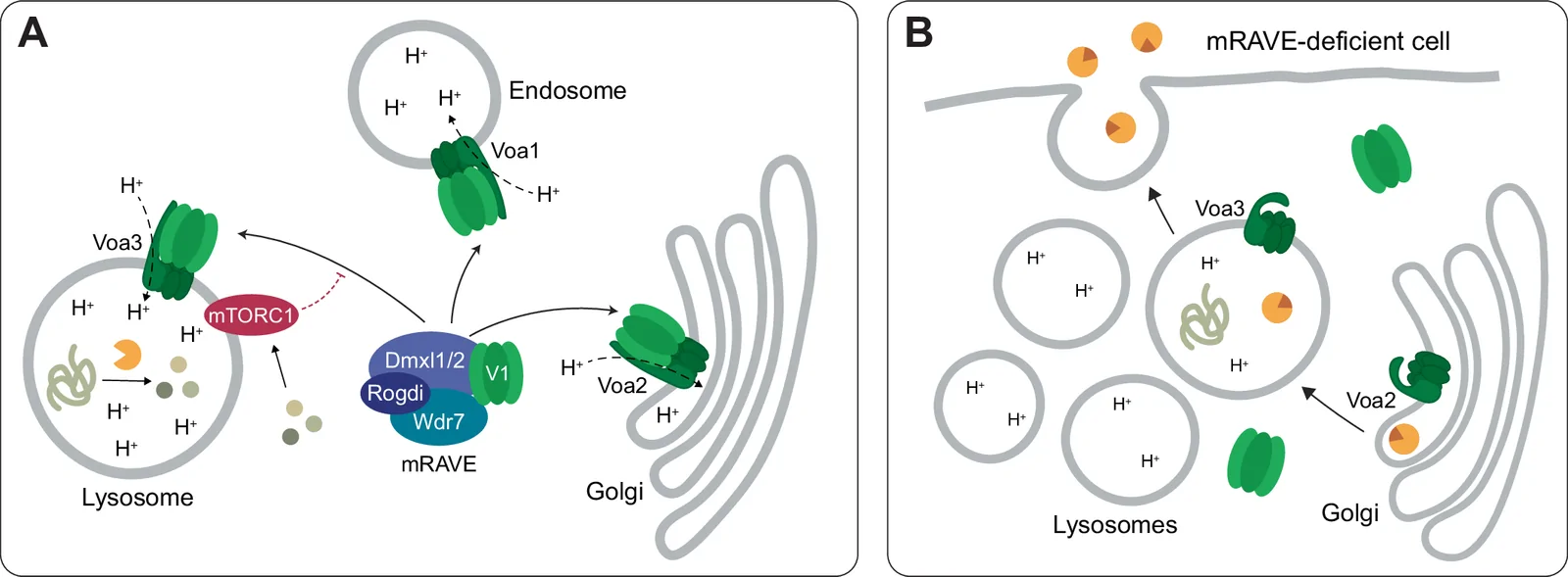
The V-ATPase assembly factor mRAVE governs organelle acidification and function. (**A**) Model for the role of mRAVE in assembling V-ATPase V_1_ domains with V_o_ domains containing the ubiquitous Voa1–3 subunits, thereby sustaining organelle acidification across the endomembrane system. mTORC1 is activated by amino acids and in turn antagonizes recruitment of mRAVE and V_1_ to lysosomes, suppressing V-ATPase assembly, acidification and breakdown of macromolecules. (**B**) mRAVE deficiency disrupts V-ATPase assembly, leading to reduced organellar acidification, accumulation of lysosomes with impaired catabolic activity, and secretion of lysosomal enzymes via lysosomal exocytosis.

Reversible association of the V-ATPase V₁ and V₀ domains is a conserved mechanism to regulate organelle acidification. Here, we establish mRAVE as the central assembly factor for mammalian V-ATPases, which supports luminal acidification across the endomembrane system, sustains lysosomal function and enables adaptive changes in lysosomal catabolism downstream of mTORC1 signaling (Fig. 8A,B).

The RAVE complex, composed of Rav1, Rav2 and Skp1, was identified in budding yeast as an organelle-specific V-ATPase assembly factor that promotes V_1_–V_o_ domain association at the vacuole (Jaskolka et al, 2021b; Seol et al, 2001). This organelle selectivity results from the specific interaction of RAVE with the vacuolar Voa subunit, Vph1, but not with its Golgi-resident homolog, Stv1 (Smardon et al, 2014). By contrast, the present study demonstrates that mRAVE mediates the assembly of V-ATPase complexes containing the ubiquitously expressed subunits Voa1–3, which target proton pumps to lysosomes, endosomes and the Golgi in mammalian cells (Collins and Forgac, 2020; Nixon and Rubinsztein, 2024). Genetic studies in mice further indicate that mRAVE assembles kidney-specific Voa4-containing V-ATPases at the plasma membrane (Eaton et al, 2024). Thus, unlike its yeast counterpart, mRAVE is broadly required for V-ATPase assembly across different membrane compartments. Despite evolutionary conservation of the V-ATPase, the metazoan RAVE-related complex differs substantially in size and composition (Lee et al, 2025; Wang et al, 2024; Winkley and Kane, 2025). The Dmxl1/2 subunit is more than twice as large as Rav1, and Wdr7 represents an additional large subunit absent from yeast RAVE. This expansion of the assembly factor may reflect the need to accommodate a broader diversity of V-ATPase complexes and integrate additional regulatory inputs in metazoan cells.

Regulation of V-ATPase assembly couples organelle acidification to stress responses and nutrient signaling. In yeast, RAVE reacidifies the vacuole by promoting V-ATPase reassembly in response to glucose refeeding following starvation-induced disassembly (Jaskolka et al, 2021a; Smardon et al, 2002). Similarly, mRAVE restores acidification of lysosomes by promoting V-ATPase assembly in response to membrane damage and resulting proton gradient collapse in mammalian cells (Lee et al, 2025; Nardone et al, 2025). Beyond this function in organelle reacidification, our findings identify a broader physiological role of mRAVE. Under basal conditions, mRAVE is required to sustain V-ATPase levels and thus normal acidification of lysosomes and the Golgi, indicating ongoing assembly of proton pumps. mTORC1 inactivation promotes the concerted recruitment of mRAVE and V₁ to lysosomes, enhancing V-ATPase assembly, luminal acidification and, consequently, catabolic activity. The mechanisms underlying RAVE recruitment to membranes remain incompletely understood, but direct interaction of yeast RAVE with the Vo domain may suffice for glucose-regulated recruitment to the vacuole (Jaskolka and Kane, 2020). In mammalian cells, lysosomal membrane damage has been shown to induce mRAVE recruitment via CASM (conjugation of ATG8 to single membranes), a non-canonical autophagy pathway (Durgan and Florey, 2022; Lee et al, 2025). mTORC1 is not involved in CASM, however, and how mRAVE recruitment to lysosomes is triggered upon mTORC1 inactivation remains unclear. Golgi V-ATPases are also assembled by mRAVE but appear not to respond to mTORC1, suggesting that regulation of V-ATPase assembly by nutrient signaling is specific to lysosomes.

Our findings demonstrate that the activity of lysosomes is remarkably sensitive to changes in luminal pH. In mRAVE-deficient cells, impaired V-ATPase assembly elevates lysosomal pH by ~0.4, leading to the accumulation of dysfunctional lysosomes. mRAVE-deficient cells also display increased lysosomal exocytosis and secretion of luminal enzymes—reminiscent of the response to lysosomal deacidification during pathogen invasion (Miao et al, 2015)—yet lysosomal proteolysis remains largely suppressed even when exocytosis is blocked. This suggests that mRAVE-dependent V-ATPase assembly is required to sustain a pH environment that supports the catabolic activity of lysosomal enzymes. mRAVE also governs Golgi acidification, although we did not observe overt Golgi phenotypes in mRAVE-deficient cells. Potential defects may be subtle or context-dependent, however, as observed in human disorders and mouse models linked to Golgi V-ATPase dysfunction (Kopp et al, 2024; Kornak et al, 2008). In contrast, mTORC1 inactivation enhances V-ATPase assembly at lysosomes, thereby decreasing lysosomal pH by ~0.2–0.4 and boosting catabolic activity (the present study; (Ratto et al, 2022)). The pronounced consequences of subtle changes in lysosomal pH indicate that regulation of V-ATPase assembly enables cells to tune the acidification of lysosomes and, consequently, their degradative activity. Lysosomal pH changes of similar magnitude have been observed in cancer, neurodegeneration and senescence (Eaton et al, 2021; Nixon and Rubinsztein, 2024; Tan and Finkel, 2023; Webb et al, 2021), suggesting that dysregulated acidification may broadly compromise cellular homeostasis in human disease.

## Methods

**Table:** Reagents and tools table

Reagent/resource	Reference or source	Identifier or catalog number
**Cell lines**		
iCas9 MEFs	(Ratto et al, 2022)	
iCas9 EPP2	(Pechincha et al, 2022)	
iCas9 MIA PaCa-2	(Pechincha et al, 2022)	
iCas9 RKO	(Groessl et al, 2025)	
Wild-type MEFs	(Ratto et al, 2022)	
U2OS	ATCC	HTB-96
HEK 293T	ATCC	CRL-3216
**Recombinant DNA**		
pCMVR8.74	Addgene	22036
pCMV-VSV-G	Addgene	8454
Gag/pol	Addgene	14887
pRRL-SFFV-rtTA3-IRES-HygroR	(Michlits et al, 2020)	
pRLL-TRE3G-Cas9-P2A-BFP	(Michlits et al, 2020)	
Dual-hU6-sgRNA-mU6-sgRNA-Ef1a-Thy1.1-P2A-NeoR/-BlastR	(Pechincha et al, 2022)	
pMaCTag-Z07	Addgene	124790
pMaCTag-Z11	Addgene	120054
pBabe	Addgene	1764
pRRL-UbC-HA-Atp6v1b2-mNeonGreen	This study	
pRRL-UbC-HA-Atp6v1b2-mScarlet-I	This study	
pLV-UbC-HA-Atp6v1b2	This study	
pLV-Ef1a-3xHA-Atp6v1b2-mNeonGreen	This study	
pLV-UbC-3xFlag-Atp6v0a1-mScarlet-I	This study	
pLV-UbC-3xFlag-Atp6v0a1	This study	
pLV-Ef1a-3xFlag-Atp6v0a2-mScarlet-I	This study	
pLV-Ef1a-3xFlag-Atp6v0a2	This study	
pLV-UbC-3xFlag-Atp6v0a3-mScarlet-I	This study	
pLV-UbC-3xFlag-Atp6v0a3	This study	
pLV-UbC-V5-Wdr7	This study	
pLV-UbC-HA-Wdr7-mNeonGreen	This study	
pLV-UbC-V5-Wdr7 sgRNA-resistant	This study	
pBabe-Dmxl1-mNeonGreen	This study	
pLV-UbC-ClCN6-Flag	This study	
pLV-UbC-ClCN6^E200A^-Flag	This study	
pLV-UbC-ClCN7-Flag-P2A-Ostm1-V5	This study	
pLV-UbC-TMEM192-3xHA	This study	
pLV-UbC-TMEM192-2xFlag	This study	
pLJC5-TMEM192-3xHA	Addgene	102930
pLJC5-TMEM192-2xFlag	Addgene	102929
MGAT-SEP-Ruby3	Addgene	200937
**Antibodies**		
rabbit anti-HA	Cell Signaling Technology	3724
rabbit anti-Golgin-97	Cell Signaling Technology	13192
rabbit anti-Lamp1	Cell Signaling Technology	3243
rabbit anti-Rab7	Cell Signaling Technology	9367
rabbit anti-Atp6v1b2	Cell Signaling Technology	14617
rabbit anti-V5	Cell Signaling Technology	13202
mouse anti-β-actin	Sigma-Aldrich	A5441
goat anti-Cathepsin B	R&D Systems	AF965
goat anti-Cathepsin L	R&D Systems	AF952
goat anti-Cathepsin D	R&D Systems	AF1029
mouse anti-Flag M2	Sigma-Aldrich	F1804
rat anti-Lamp2	Origene	AM08170BT-N
rabbit anti-Atp6v0d1	Abcam	ab202899
rabbit anti-Atp6v1a	Abcam	ab199326
rabbit anti-Atp6v1e1	ProteinTech	15280-1-AP
rabbit anti-Lamp1	Santa Cruz Biotechnology	sc-19992
rabbit anti-CD63	Abcam	ab217345
rabbit anti-EEA1	Cell Signaling Technology	3288
rabbit anti-Calreticulin	Cell Signaling Technology	12238
rabbit anti-TOM20	ProteinTech	11802-1-AP
rabbit anti-VDAC	Cell Signaling Technology	4661
rabbit anti-S6K	Cell Signaling Technology	2708
rabbit anti-S6K pT389	Cell Signaling Technology	9234
rabbit anti-Histone H3	Cell Signaling Technology	4499
mouse anti-GM130	BD Biosciences	610822
scFv M6P	(Müller-Loennies et al, 2010)	N/A
mouse anti-Myc-Tag	Cell Signaling Technology	2276
rabbit anti-Tfeb	ProteinTech	13372-1-AP
rabbit anti-4E-BP1	Cell Signaling Technology	9452
rabbit anti-Alexa Fluor 488	Life Technologies	A11034
rat anti-Alexa Fluor 488	Life Technologies	A11006
mouse anti-Alexa Fluor 568	Life Technologies	A11004
rat anti-Alexa Fluor 647	Life Technologies	A21247
HRP anti-goat	Life Technologies	31402
HRP anti-mouse	Cytiva	NA931
HRP anti-rabbit	Cytiva	NA934
HRP anti-rat	Jackson ImmunoResearch	112-035-143
**Oligonucleotides and other sequence-based reagents**		
sgRNA sequences	This study	Appendix Table S1
Genotyping PCR primers	This study	Appendix Table S2
RT-qPCR primers	This study	Appendix Table S3
cDNA cloning primers	This study	Appendix Table S4
**Chemicals, enzymes and other reagents**		
Torin 1	Tocris	4247
Nigericin	Tocris	4312/10
Bafilomycin A1	Cayman	11038
Valinomycin	MedChemExpress	HY-N6693
Monesin	Selleckchem	S2324
Halt Protease and Phosphatase Inhibitor Cocktail	Thermo Fisher Scientific	78444
Doxycycline	Sigma-Aldrich	J67043
PEI	Polysciences	23966
Polybrene	Sigma-Aldrich	TR-1003
Blasticidin	Santa Cruz Biotechnology	sc-495389
Hygromycin	Invitrogen	10687010
Geneticin	Invitrogen	10131035
Fatty acid-free BSA	EMD Millipore	126579
Clarity Max Western ECL substrates	Bio-Rad	1705062
SuperSignal West Atto	Thermo Fisher Scientific	A38554
Triton X-100	Sigma-Aldrich	X100
TRIzol	Invitrogen	15596026
Chloroform	Carl Roth	7331
Isopropanol	Serva	39559
Ethanol	Th. Geyer	11832330
SYBR green master mix	Life Technologies	4309155
Pierce anti-HA magnetic beads	Thermo Fisher Scientific	88836
Oregon Green 488 dextran 10 kDa	Life Technologies	D7170
Alexa Fluor 647 dextran 10 kDa	Life Technologies	D22914
FITC-dextran 10 kDa	Life Technologies	D1820
Dextran 10 kDa	Thermo Fisher Scientific	D1860
DQ Green BSA	Life Technologies	D12050
Hoechst	Cayman	15547
LysoTracker Red	Life Technologies	L7528
SiR-Lysosome	SpiroChrome	SC012
Albumin bovine Fraction V (receptor grade)	Serva	11924.02
Pierce BCA Protein Assay Kit	Fisher Scientific	18064090
Bio-Rad Protein Assay Kit	Bio-Rad	5000001
Amicon Ultra–15 Centrifugal Filters Ultracel – 10 K	Merck Millipore	UFC901024
MycoAlertMycoplasma Detection Kit	Lonza	126579
SuperScript IV Reverse Transcriptase	Thermo Fisher Scientific	18090200
Random Hexamers	Thermo Fisher Scientific	N8080127
Q5 High-Fidelity DNA Polymerase	NEB	M0491
DMEM/F-12 (for -AA experiments)	USBiological	D9807-11
Dialyzed FBS	Capricorn	FBS-DIA-12A
**Software**		
NEBuilder Assembly Tool version 2.10.8	NEB	
ImageJ 1.54 f (Fiji)	(Schindelin et al, 2012)	
Prism Version 10.4.1	GraphPad	
ZEN Blue 3.7.97.07000	Carl Zeiss Microscopy	
ZEN Black 8.1.0.484	Carl Zeiss Microscopy	
Spectronaut 20.2.250922.92449	(Bruderer et al, 2015)	
MaxQuant 2.1.4.0	(Tyanova et al, 2016)	
R version 4.5.1	R Foundation for Statistical Computing	
R package limma_3.64.1	(Ritchie et al, 2015)	
R package vsn_3.76.0	(Huber et al, 2002)	
R package missForest_1.5	(Stekhoven and Bühlmann, 2012)	

### Cell lines and cell culture conditions

Gene editing experiments were conducted in single-cell-derived doxycycline-inducible Cas9 (iCas9) cell lines (MEFs, EPP2, MIA PaCa-2 and RKO) described previously (Groessl et al, 2025; Pechincha et al, 2022; Ratto et al, 2022); HA-V1B2–V5-Wdr7 co-IP were conducted in wild-type MEFs. Cells were cultured in DMEM/F-12 supplemented with 10% fetal bovine serum and 2 mM L-glutamine. HEK 293T cells were used for virus production and cultured in DMEM supplemented with 10% FBS and 2 mM L-glutamine. All cell culture reagents were obtained from Gibco unless otherwise specified. Cells were cultured at 37 °C in a humidified incubator with 5% CO₂. Cell lines were routinely tested for mycoplasma contamination with the MycoAlertMycoplasma Detection Kit; human cancer cell lines were authenticated by single nucleotide polymorphism (SNP) typing (Multiplexion).

Amino acid starvation experiments were conducted with amino acid- and glucose-free DMEM/F-12. Glucose and bicarbonate were added to concentrations of standard DMEM/F-12; pH was adjusted to 7.3 with HCl. Media were supplemented with 10% dialyzed FBS. For amino acid-replete conditions, amino acids were added at DMEM/F-12 concentrations with an additional 2 mM L-glutamine. Amino acids, glucose, and bicarbonate were from Sigma-Aldrich.

### Lenti- and retrovirus production and cell transduction

Lentiviral particles were produced by co-transfecting HEK 293T cells with a lentiviral expression vector, packaging plasmid pCMVR8.74 and envelope plasmid pCMV-VSV-G, using polyethylenimine (PEI MW 25,000). Retroviral particles were produced by co-transfecting HEK 293T cells with retroviral expression vector, packaging plasmid Gag-Pol and envelope plasmid pCMV-VSV-G, using PEI. Viral supernatants were collected 48 h post-transfection, filtered through a 0.45 μm PES filter, and used to transduce target cells in the presence of 10 μg/ml polybrene. Lentiviral supernatant was used at a 1:10 dilution, retroviral supernatant at a 1:1 dilution.

### Generation of gene knockouts

For the generation of inducible knockouts (iKO) with CRISPR/Cas9, iCas9 cell lines were transduced with lentiviral vectors encoding dsgRNAs targeting the respective gene: Dual-hU6-sgRNA-mU6-sgRNA-Ef1a-Thy1.1-P2A-NeoR, Dual-hU6-sgRNA-mU6-sgRNA-Ef1a-Thy1.1-P2A-BlastR, or Dual-hU6-sgRNA-mU6-sgRNA-EF1a-BFP. DsgRNA-expressing cells were selected with neomycin or blasticidin or sorted by FACS for BFP expression. Cells were cultured in antibiotics until seeded for the experiment to maintain dsgRNA expression, and Cas9 was induced with 0.5 μg/ml doxycycline for 4 days. Control dsgRNAs were Ctrl1 targeting a non-coding chromosome region (Chr1.3) and Ctrl2 targeting a non-expressed gene (CD19). Ctrl1 and Wdr7 iKO1 were used for knockout experiments if not otherwise indicated. dsgRNA sequences are listed in Appendix Table S1.

### PCR validation of CRISPR/Cas9 editing

The efficiency of CRISPR/Cas9 editing in iKOs was validated by PCR with primers flanking the genomic locus targeted by the sgRNAs. Genomic DNA was extracted with the DNeasy Blood & Tissue Kit and PCR performed using a reaction mix containing 2x PCR buffer (50 mM Tris-HCl, pH 8.3; 50 mM KCl; 2.5 mM MgCl₂), 10 µM primer mix, 1.5 mM MgCl₂, 0.2 mM dNTPs, 200 mM betaine, and 5 U/µl Taq DNA Polymerase. PCR products were analyzed with agarose gel electrophoresis. Genomic PCR primers are listed in Appendix Table S2.

### RT–qPCR

RNA was isolated from cultured cells using TRIzol according to the manufacturer’s instructions. Briefly, cells were lysed directly in TRIzol and incubated for 5 min at room temperature. Chloroform was added, and samples were centrifuged at 18,000 × *g* for 15 min at 4 °C. The aqueous phase was collected; RNA was precipitated with isopropanol and incubated for 10 min at room temperature. Samples were incubated at −80 °C for 2 h, followed by centrifugation at 18,000 × *g* for 10 min. RNA pellets were washed with 75% ethanol, air-dried, and resuspended in RNase-free water. RNA concentration and purity were determined with a NanoDrop One.

cDNA was synthesized from 1 µg of RNA using SuperScript IV Reverse Transcriptase and random hexamer primers. qPCR was performed with the SYBR Green Master Mix. Samples were measured in technical duplicates and averaged. Relative gene expression was calculated using the delta-delta Ct method (2^−ΔΔCt^). Expression levels were normalized to the geometric mean of two control genes, Tbp and Gapdh. qPCR primers are listed in Appendix Table S3.

### Generation of cDNA expression constructs

Atp6v1b2, Atp6v0a1, Atp6v0a2, Atp6v0a3, and Wdr7 cDNAs were amplified by PCR from a mouse cDNA library generated by reverse transcription of mRNA isolated from MEFs; Dmxl1-mNeonGreen from a template synthesized by Twist Bioscience; OSTM1 cDNA from a human cDNA library generated from RKO cells (human); ClCN6 (ClC-6) and ClCN7 (ClC-7) cDNAs from human Gateway ORF clones (provided by the Genomics and Proteomics Core Facility, DKFZ); and TMEM192-3xHA and TMEM192-2xFlag from Addgene plasmids #102930, #102929 (Abu-Remaileh et al, 2017). N- or C-terminal HA, Flag and V5 tags were introduced with the PCR primer. mNeonGreen and mScarlet-I were amplified from pMaCTag-Z07 (Addgene #124790) and pMaCTag-Z11 (Addgene #120054), respectively, and fused to the cDNA of interest with a GGGS linker. CLCN7 and OSTM1 cDNAs were cloned into the same expression vector, separated by a P2A cleavage site, to express equal quantities of the two transporter subunits. sgRNA-resistant Wdr7 cDNA for genetic rescue experiments and proton transport-deficient ClC-6^E200A^ (Neagoe et al, 2010) were generated using the Qiagen Site-Directed Mutagenesis Kit.

Cloning primers were designed using the NEBuilder Assembly Tool, and cloning was performed with the Q5 HiFi polymerase and the HiFi DNA Assembly Master Mix following the manufacturer’s instructions. Expression plasmids generated in this study are listed in the reagents and tools table. cDNA cloning primers are listed in Appendix Table S4.

### Cell proliferation

Cells were maintained in medium containing the corresponding antibiotic, and Cas9 expression was induced with 0.5 µg/ml doxycycline for 4 days prior to the experiment. Cells were seeded in standard medium without any other supplements in 12-well plates in three technical replicates. Cell numbers were counted with a CASY Cell Counter (OMNI Life Science) on day 0 (>4 h after seeding) and then daily or on day 3 as indicated.

### Immunoblotting

Cells were washed with ice-cold PBS and lysed in lysis buffer (50 mM HEPES, pH 7.4, 40 mM NaCl, 2 mM EDTA, 1% Triton X-100, supplemented with 1 mM sodium orthovanadate, 50 mM sodium fluoride, 10 mM sodium pyrophosphate, 10 mM sodium glycerophosphate, Halt protease, and phosphatase inhibitor cocktail). Lysates were incubated for 15 min on ice and cleared by centrifugation at 18,000 × *g* for 5 min at 4 °C. Protein concentration was measured by the BCA assay.

To analyze secreted proteins, cells were cultured in Opti-MEM for 24 h. Cellular supernatants were centrifuged at 1000 × *g* for 5 min at 4 °C, then at 18,000 × *g* for 20 min at 4 °C. Cell numbers were quantified from parallel culture plates with a CASY Cell Counter and used to normalize cellular supernatant volumes.

Proteins were denatured in Laemmli buffer containing 10% β-mercaptoethanol for 10 min at 95 °C. Equal amounts of protein were analyzed by SDS gel electrophoresis and immunoblotting on nitrocellulose membranes following standard protocols. When samples were probed across multiple membranes, blotting was performed in parallel, under identical conditions. Primary antibodies were used at a 1:1000 dilution in TBS-T (20 mM Tris, 150 mM NaCl, and 0.1% Tween) with 5% BSA, secondary antibodies at a 1:5000 dilution in TBS-T with 5% skim milk. Immunoblots were imaged with the ChemiDoc Touch system (Bio-Rad) using ECL substrates Clarity Max Western. For stripping, membranes were incubated in stripping buffer (200 mM glycine, 0.1% SDS, pH 2.2), washed three times with TBS-T (10 min each) and re-blocked with 5% skim milk in TBS-T for 30 min. Band-integrated intensities were quantified with ImageJ.

M6P modification of lysosomal enzymes was analyzed in supernatants from cells in which secretion of newly synthesized enzymes was induced by NH_4_Cl. To this end, cells were cultured in Opti-MEM supplemented with 10 mM NH₄Cl for 24 h. Supernatants were centrifuged at 1000 × *g* for 5 min at 4 °C, then at 18,000 × *g* for 20 min at 4 °C. Resulting supernatants were concentrated ~10-fold with an Amicon Ultra–15 Centrifugal Filter, 10 kDa molecular weight cut-off. Immunodetection was conducted as above with the following changes: blocking and primary antibody solutions contained 3% receptor-grade BSA in TBS-T; washing steps were five times TBS-T; M6P-scFv was used at 5 µg/ml overnight and detected with Myc-Tag antibody (4 h at room temperature) followed by anti-mouse HRP-conjugated (2 h at room temperature).

### Isolation of lysosome-enriched membrane fractions

Lysosomes were loaded overnight with 2 mg/ml 10 kDa dextran, followed by a 4 h chase in fresh medium. Cells were washed, resuspended in homogenization buffer (20 mM HEPES, pH 7.5, 125 mM KCl, 50 mM sucrose, 1 mM EDTA, protease and phosphatase inhibitors) and homogenized using 12 strokes of a KIMBLE Dounce tissue grinder (large clearance pestle, Sigma-Aldrich). Post-nuclear supernatants were obtained by centrifugation at 800 × *g* for 5 min at 4 °C. Membranes were subsequently pelleted at 18,000 × *g* for 20 min at 4 °C. Pellets were lysed as above and analyzed by immunoblotting. Protein concentrations were determined using the Bio-Rad Protein Assay Kit.

### Co-immunoprecipitation

For co-IP, cells were seeded at 3 × 10^5^ cells in six-well plates (immunoblotting) or at 4 × 10^6^ cells in 15 cm dishes (proteomics) 1 day prior to the experiment. Cells were washed once with ice-cold PBS, scraped with 300 µl (six-well plates) or 1 ml (15-cm dishes) of IP buffer (V_1_–V_o_ co-IP: 40 mM HEPES, pH 7.4, 150 mM NaCl, 2 mM EDTA, 2.5 mM MgCl₂, 5% glycerol; V_1_ – Wdr7 co-IP: 40 mM HEPES pH 7.4, 150 mM NaCl; 1% Triton X-100, protease and phosphatase inhibitors were added immediately before use), collected into precooled tubes and incubated on ice for 15 min. Lysates were centrifuged at 11,500 rpm for 5 min at 4 °C and supernatants were transferred to new precooled tubes. During lysis, magnetic beads were equilibrated by washing three times with IP buffer. 10% of the lysate was collected as input and mixed with 4x Laemmli buffer. The remaining lysate was incubated with antibody-coupled magnetic beads for 2 h at 4 °C with end-to-end rotation. Beads were washed once with IP buffer, resuspended in 2x Laemmli and heated at 65 °C for 10 min at 500 rpm. Beads were removed using a magnetic rack, and supernatants were used for SDS–PAGE or LC–MS analysis. 1–2% of the input was loaded as indicated.

### Lysosomal immunoprecipitation

For lysosomal immunoprecipitation (Lyso-IP), 3 ×10^6^ MEFs stably expressing pLV-UbC-TMEM192-3xHA (Lyso-IP) or pLV-UbC-TMEM192-2xFlag (Ctrl-IP) were seeded into 15 cm dishes 1 day before the experiment. Magnetic beads were equilibrated by washing three times with 1 ml KPBS (136 mM KCl, 10 mM KH₂PO₄, pH 7.25, protease and phosphatase inhibitors) and kept on ice. Cells were washed twice with ice-cold PBS, scraped into 1 ml ice-cold KPBS, centrifuged at 1000 × *g* for 2 min at 4 °C and resuspended in 950 µl KPBS. About 50 µl of cell suspension were taken as input. Remaining suspensions were homogenized with 20 strokes in a KIMBLE Dounce tissue grinder (large clearance pestle, Sigma-Aldrich) on ice. Homogenates were centrifuged at 1000 × *g* for 2 min at 4 °C and incubated with 60 µl of equilibrated magnetic beads for 5 min at 4 °C with end-to-end rotation. Beads were washed three times with ice-cold KPBS, resuspended in 90 µl lysis buffer and incubated for 10 min on ice. Beads were removed using a magnetic rack, supernatants were mixed with 4x Laemmli buffer and proteins were denatured at 65 °C for 15 min. Input samples were incubated with 90 µl lysis buffer for 20 min on ice, centrifuged at 13,000 × *g* for 10 min at 4 °C and the resulting supernatants were mixed with 4x Laemmli buffer.

### Sample preparation for proteomics

For Lyso-IP and co-IP analyses, samples were prepared as described above. For cellular proteome analysis, cells were washed twice with ice-cold DPBS, scraped into 1 ml ice-cold DPBS and centrifuged at 1000 × *g* for 2 min at 4 °C. Pellets were resuspended in lysis buffer (50 mM HEPES, pH 7.4, 40 mM NaCl, 2 mM EDTA, 1% Triton X-100, 1 mM sodium orthovanadate, 50 mM sodium fluoride, 10 mM sodium pyrophosphate, 10 mM sodium glycerophosphate, Halt protease and phosphatase inhibitor cocktail), incubated for 20 min on ice and cleared by centrifugation at 13,000 × *g* for 10 min at 4 °C. Supernatants were collected and mixed with 4x Laemmli buffer. For all analyses, 5 µg of protein were digested with trypsin with an AssayMAP Bravo liquid handling system (Agilent Technologies) running the autoSP3 protocol (Müller et al, 2020).

For secretome analysis, cellular supernatants were collected as described above, centrifuged for 5 min at 15,000 rpm at room temperature and transferred to new tubes. Samples were mixed with an equal amount of RIPA buffer, and protein concentration was determined by BCA assay. About 10 µg of proteins were digested with trypsin with an AssayMAP Bravo liquid handling system (Agilent Technologies) running the autoSP3 protocol (Müller et al, 2020).

### LC–MS/MS analysis

Dried peptide samples were reconstituted in 97.4% H_2_O, 2.5% hexafluoro-2-propanol and 0.1% trifluoroacetic acid, and one third was used for subsequent analysis. LC– MS/MS was carried out with a Vanquish Neo UPLC system (Thermo Fisher Scientific) for Lyso-IP, cell lysate and secretome analyses or with an Ultimate 3000 UPLC system (Thermo Fisher Scientific) for co-IP analysis, directly connected to an Orbitrap Exploris 480 mass spectrometer for a total of 60 min (Lyso-IP, secretome), 90 min (co-IP) or 120 min (cell lysate) per sample. Peptides were online desalted on a trapping cartridge (Acclaim PepMap300 C18, 5 µm, 300 Å wide pore; Thermo Fisher Scientific) with a loading volume of 60 µl using 30 µl/min flow of 0.05% trifluoroacetic acid in H_2_O (Vanquish Neo UPLC) or for 3 min at 30 µl/min flow (Ultimate 3000 UPLC). The analytical multistep gradient (300 nl/min) was performed with a nanoEase MZ Peptide analytical column (300 Å, 1.7 µm, 75 µm ×  200 mm, Waters) using solvent A (0.1% formic acid in H_2_O) and solvent B (0.1% formic acid in acetonitrile). For 45 min (secretome, Lyso-IP), 72 min (co-IP) or 104 min (cell lysate) the concentration of B was linearly ramped from 4% (cell lysate, co-IP) or 5% (secretome, Lyso-IP) to 30%, followed by a quick ramp to 78% (co-IP) or 80% (secretome, cell lysate, Lyso-IP), after 2 min (co-IP) or 4 min (secretome, cell lysate, Lyso-IP) the concentration of B was lowered to 2% and 10 min (co-IP) or a three column volumes (secretome, cell lysate, Lyso-IP) equilibration appended. Eluting peptides were analyzed in the mass spectrometer using data-independent acquisition (DIA) mode (secretome, cell lysate, Lyso-IP) or data-dependent acquisition (DDA) mode (co-IP).

For DIA, a full scan at 120k resolution (350–1400 m/z, 300% AGC target, 45 ms maxIT) was followed by 20 (secretome), 25 (Lyso-IP), or 40 (cell lysate) DIA windows. The DIA acquisition covered a mass range of 400–1000 m/z (secretome, cell lysate) or 380–880 m/z (Lyso-IP) using windows of variable width. Windows overlapped by 1 m/z, the AGC target was 1000% with a maxIT of 54 ms and spectra were recorded at a resolution of 30k.

For DDA, a full scan at 60k resolution (380–1400 m/z, 300% AGC target, 45 ms maxIT) was followed by up to 2 s of MS/MS scans. Peptide features were isolated with a window of 1.4 m/z, fragmented using 26% NCE. Fragment spectra were recorded at 15k resolution (100% AGC target, 54 ms maxIT). Unassigned and singly charged eluting features were excluded from fragmentation, and dynamic exclusion was set to 30 s.

Each sample was followed by a wash run (22 min) to minimize carry-over between samples. Instrument performance throughout the course of the measurement was monitored by regular (approx. one per 48 h) injections of a standard sample to track performance in terms of protein group IDs, TIC, MS1-, and MS2-error.

### Proteomics data analysis

Analysis of DIA RAW files was performed with Spectronaut (Biognosys, version 20.2.250922.92449 (Bruderer et al, 2015)) in directDIA+ (deep) library-free mode. Default settings were applied with the following adaptations. Within DIA analysis under identification, the precursor PEP cutoff was set to 0.01, the protein *Q* value cutoff (Run) to 0.01, the protein PEP cutoff to 0.01, and run-level protein scoring to the highest scoring observation (SN19 default). In quantification, the proteotypicity filter was set to only protein group-specific and the protein LFQ method to MaxLFQ; cross-run normalization was disabled (secretome, Lyso-IP) or enabled (cell lysate), and the quantification window was set to not synchronized (SN17). In post analysis, use all MS-level quantities was set to true. Data were searched against the mouse proteome from Uniprot (mouse reference database with one protein sequence per gene, containing 21,757 unique entries from January 2025), Cas9 sequence (secretome, cell lysate, and Lyso-IP) and contaminants FASTA from MaxQuant (246 unique entries from 22 August 2025). Conditions were included in the setup.

For DDA, data analysis was carried out in MaxQuant (version 2.1.4.0 (Tyanova et al, 2016)), using an organism-specific database extracted from Uniprot (mouse reference database with one protein sequence per gene, containing 21,757 unique entries from January 2025). Default settings were applied with the following adaptations. Match between runs (MBR) was enabled to transfer peptide identifications across RAW files based on accurate retention time and m/z. Fractions were set in a way that MBR was only performed within replicates. Label-free quantification (LFQ) was enabled with default settings. iBAQ-value (Schwanhäusser et al, 2011) generation was enabled.

### Proteomics statistical analysis

For the statistical analysis, log2-transformed quantity values (secretome, cell lysate, Lyso-IP) or intensity values (co-IP) were used. The following steps were performed per statistical contrast and not across the whole data matrix. Protein groups with valid values in 70% of the samples of at least one condition were used for statistics. Variance stabilization normalization (Huber et al, 2002) was applied to normalize across samples. Adapted from the Perseus recommendations (Tyanova and Cox, 2018), missing values completely absent in one condition were imputed with random values drawn from a downshifted (2.2 standard deviations) and narrowed (0.3 standard deviations) intensity distribution of the individual samples. For missing values with no complete absence in one condition, the R package missForest was used for imputation (Stekhoven and Bühlmann, 2012). The statistical analysis was performed with the R package limma (Ritchie et al, 2015); single contrasts were adapted from the user guide chapter *Two Group*. The setup was adapted for proteomics data via setting the *eBayes* options *trend* and *robust* to *TRUE*. Proteins with a minimum of two identified peptides in at least three replicates were considered for further analysis. Wdr7 iKO Lyso-IP was analyzed using a subproteome curated from lysosome-enriched proteins identified in this study, the UniProt reference proteome ‘mouse lysosome’ and previous publications (Ratto et al, 2022; Wyant et al, 2018).

### Live-cell imaging

Live-cell imaging was performed in a humidified chamber at 37 °C and 5% CO₂. Hoechst 33342 (0.5 µg/ml) was added 30 min before imaging. Where indicated, cells were treated with Torin 1 [400 nM] directly on the microscope stage. Confocal microscopy was performed with a Zeiss LSM780 SD or LSM900 Airyscan 2 microscope equipped with a 40x or 63x (NA 1.40) oil immersion objective. For live imaging, cells were seeded in eight-well chambered IBIDI coverslips and left to attach overnight. Lysosomal proteolysis, abundance and acidity were monitored by incubating cells with 0.1 mg/ml DQ Green BSA for 4 h, with 50 nM LysoTracker Red added for the final 1 h prior to imaging. Activated cathepsin D was measured by incubating cells with 1 µM SiR-Lysosome for 1 h. V-ATPase-dependent lysosomal reacidification was measured by FITC-dextran quenching. Cells were incubated overnight with 1 mg/ml FITC-dextran (10 kDa), followed by a 4 h chase in fresh medium. V-ATPase-mediated reacidification of lysosomes was monitored by quantifying the rate of FITC fluorescence quenching in neutralized lysosomes after relief of V-ATPase inhibition. To this end, cells were treated with 20 nM bafilomycin A1 for 30 min. Bafilomycin A1 was removed by washing cells with medium containing 5 mg/ml fatty acid-free BSA, and FITC fluorescence quenching was imaged every 3 min for 60 min.

### Organelle pH measurements

To measure lysosomal pH, cells were seeded in eight-well chambered IBIDI coverslips. The following day, cells were incubated with 0.1 mg/ml Oregon Green 488 dextran (10 kDa) and 0.1 mg/ml Alexa Fluor 647 dextran (10 kDa) for 4 h. After a 4 h chase in fresh medium, cells were treated as indicated. To generate a pH calibration curve, cells were incubated with calibration buffers (125 mM KCl, 25 mM NaCl, 25 mM MES, pH 4.0–6.5) containing 10 µM nigericin, 10 µM monensin, and 10 µM valinomycin to clamp lysosomal pH to buffer pH. Fluorescence was measured by confocal microscopy. Lysosomal pH was calculated based on the ratio of Oregon Green (pH-sensitive) to Alexa Fluor 647 (pH-insensitive) fluorescence using the standard curve.

To measure Golgi pH, cells were stably transduced with MGAT-SEP-mRuby3 (Liu et al, 2023) and sorted by flow cytometry with the 488 nm and 568 nm lasers to establish a cell population with homogeneous expression of both fluorophores (50–70% fluorescence brightness). For imaging, cells were seeded in eight-well chambered IBIDI coverslips. The following day, cells were stained with 0.5 µg/ml Hoechst 33342 30 min prior to imaging. To generate a pH calibration curve, control cells were incubated in calibration buffers (125 mM KCl, 25 mM NaCl, 25 mM HEPES (pH 7.0–7.5)/MES (pH 6.0–6.5)) containing 10 µM nigericin, 10 µM monensin and 10 µM valinomycin to clamp intracellular pH to buffer pH. Fluorescence was measured by confocal microscopy. Golgi pH was calculated based on the ratio of SEP (pH-sensitive) to mRuby3 (pH-insensitive) fluorescence using the standard curve.

### Immunofluorescence

Cells were seeded onto IBIDI chamber slides and cultured for 16–24 h. Cells were rinsed with ice-cold PBS, fixed with 4% paraformaldehyde in PBS for 15 min at room temperature and permeabilized with 0.05% Triton X-100 in PBS for 5 min. After washing with PBS, cells were blocked for 30 min in blocking solution (4% normal goat serum, 0.5% BSA in PBS). Primary antibodies were diluted 1:400 in blocking solution and applied overnight at 4 °C. After two washes with blocking solution, cells were incubated with Alexa Fluor-conjugated secondary antibodies (1:1000 dilution) in blocking solution for 2 h. Cells were washed with PBS, stained with 10 μg/ml Hoechst 33342 in PBS for 5 min, washed twice with PBS and imaged immediately. For cell-surface staining of LAMP1, cells were stained as above, but the permeabilization step was omitted.

### Microscopy images and analysis

For visualization of microscopy data, maximum intensity z-projections are shown for cellular DQ BSA, lysotracker and Lamp1/2 levels, single z-planes for subcellular localization of mRAVE and V_1_ and other colocalization analyses. Fluorescence intensity was quantified with the particle analyzer function of ImageJ (Schindelin et al, 2012) in randomly chosen fields of view across the entirety of each sample. Mean cellular fluorescence was determined by normalizing the integrated signal density of the respective fluorescent probe to cell number. Pearson’s coefficients were calculated using the JACoP plugin in ImageJ. Where indicated, individual data points in super blots were normalized to the mean value of the corresponding control group.

### Statistical analysis

Data were represented as mean ± SD for technical replicates and independent biological replicates with a single observation or as mean ± SEM for independent biological experiments with multiple observations. Statistical analyses were performed with Excel for immunoblot quantifications and with Graphpad Prism for all the other quantifications. *p* values were calculated using a two-tailed unpaired *t*-test assuming equal variance; for data in which controls were normalized to 1, *p* values were calculated using a two-tailed unpaired *t*-test with Welch correction.

## Supplementary information


Appendix
Peer Review File
Dataset EV1
Dataset EV2
Dataset EV3
Dataset EV4
Dataset EV5
Source data Fig. 1
Source data Fig. 2
Source data Fig. 3
Source data Fig. 4
Source data Fig. 5
Source data Fig. 6
Source data Fig. 7
Expanded View Figures


## Data Availability

The mass spectrometry data generated in this study have been deposited at ProteomeXchange via the PRIDE partner repository: Lyso-IP enrichment and Lyso-IP Wdr7 iKO (PXD070950), HA-V1b2 co-IP (PXD070848), cellular proteome Wdr7 iKO (PXD070900) and secretome Wdr7 iKO and Dmxl1 + Dmxl2 iKO (PXD070740). The source data of this paper are collected in the following database record: biostudies:S-SCDT-10_1038-S44318-026-00838-5.
