# Supplementary material for: mRAVE governs lysosomal catabolism through basal and mTORC1-regulated V-ATPase assembly
Source: EMBO J. 2026 Jun 22;45(17):5988–6020. doi: 10.1038/s44318-026-00838-5 (PMC13534531; doi:10.1038/s44318-026-00838-5)
Supplement: Supplementary file 1 [file 44318_2026_838_MOESM15_ESM.pdf]

Expanded View Figures

Figure EV1. mRAVE CRISPR editing and Lyso-IP quality controls (related to Fig. 1).

(**A**) CRISPR editing of mRAVE subunits in iKO MEFs cells after Cas9 induction for 4 days with doxycycline [0.5 µg/ml]. Successful editing was confirmed by PCR using primers that amplify the genomic locus spanning the cutting sites of the respective sgRNAs. Each gene was disrupted using two distinct sgRNA pairs. (**B**) Proliferation of Wdr7 iKO2 MEFs expressing sgRNA-resistant V5-Wdr7 cDNA. (**C**) Fold change (FC) in cell number of Wdr7 iKO2 RKO cells expressing sgRNA-resistant V5-Wdr7 cDNA after 3 days of proliferation. (**D**) Proliferation of Rogdi iKO MEFs. (**E**) Enrichment/depletion of organelle markers in Lyso-IP from MEFs, analyzed by immunoblotting. (**F**) Lysosome enrichment, analyzed by Lyso-IP and label-free LC–MS in MEFs. (**G**) Organelle enrichment/depletion, analyzed by Lyso-IP and label-free LC–MS in MEFs. (**H**) Lysosomal pH in Dmxl1 + 2 iKO MEFs, quantified by ratiometric imaging of 10 kDa Dextran Oregon Green and Alexa Fluor 647. (**B–D**) Data are mean ± SD ($n = 3$ technical replicates). (**F**, **G**) $n = 4$ independent experiments. (**H**) Data are replicate mean ± SEM (closed circles; $n = 3$ independent experiments) and fields of view (open circles; ≥9 per replicate). p values were calculated using a two-tailed unpaired t-test.

© The Author(s)

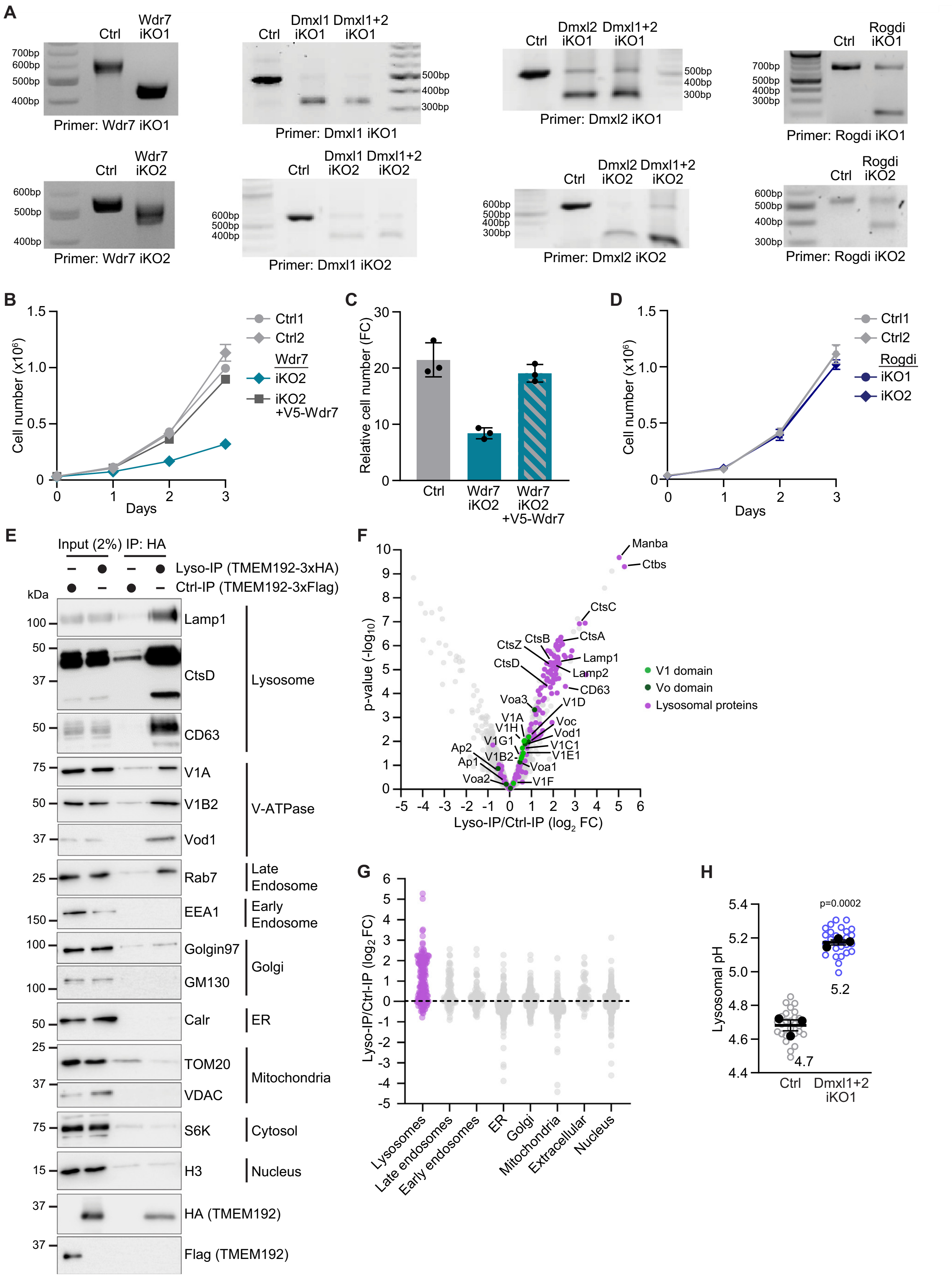
A
Ctrl
Wdr7 iKO1
700bp
600bp
500bp
400bp
Primer: Wdr7 iKO1
Ctrl
Dmxl1 iKO1
Dmxl1+2 iKO1
500bp
400bp
300bp
Primer: Dmxl1 iKO1
Ctrl
Dmxl2 iKO1
Dmxl1+2 iKO1
500bp
400bp
300bp
Primer: Dmxl2 iKO1
Ctrl
Rogdi iKO1
700bp
500bp
400bp
300bp
Primer: Rogdi iKO1
Ctrl
Wdr7 iKO2
600bp
500bp
400bp
Primer: Wdr7 iKO2
Ctrl
Dmxl1 iKO2
Dmxl1+2 iKO2
600bp
500bp
400bp
Primer: Dmxl1 iKO2
Ctrl
Dmxl2 iKO2
Dmxl1+2 iKO2
600bp
500bp
400bp
300bp
Primer: Dmxl2 iKO2
Ctrl
Rogdi iKO2
600bp
500bp
400bp
300bp
Primer: Rogdi iKO2
B
Cell number (x10⁶)
Days
Ctrl1
Ctrl2
Wdr7
iKO2
iKO2 +V5-Wdr7
C
Relative cell number (FC)
Ctrl
Wdr7 iKO2
Wdr7 iKO2 +V5-Wdr7
D
Cell number (x10⁶)
Days
Ctrl1
Ctrl2
Rogdi
iKO1
iKO2
E
Input (2%)
IP: HA
Lyso-IP (TMEM192-3xHA)
Ctrl-IP (TMEM192-3xFlag)
kDa
Lamp1
CtsD
CD63
Lysosome
V1A
V1B2
Vod1
V-ATPase
Rab7
Late Endosome
EEA1
Early Endosome
Golgin97
GM130
Golgi
Calr
ER
TOM20
VDAC
Mitochondria
S6K
Cytosol
H3
Nucleus
HA (TMEM192)
Flag (TMEM192)
F
p-value (-log10)
Lyso-IP/Ctrl-IP (log2 FC)
Manba
Ctbs
CtsC
CtsA
CtsB
CtsZ
Lamp1
CtsD
Lamp2
CD63
Voa3
V1D
V1A
Voc
V1H
Vod1
V1G1
V1C1
Ap2
V1E1
V1B2
Ap1
Voa1
Voa2
V1F
V1 domain
Vo domain
Lysosomal proteins
G
Lyso-IP/Ctrl-IP (log2 FC)
Lysosomes
Late endosomes
Early endosomes
ER
Golgi
Mitochondria
Extracellular
Nucleus
H
Lysosomal pH
p=0.0002
5.2
4.7
Ctrl
Dmxl1+2 iKO1

 © The Author(s)

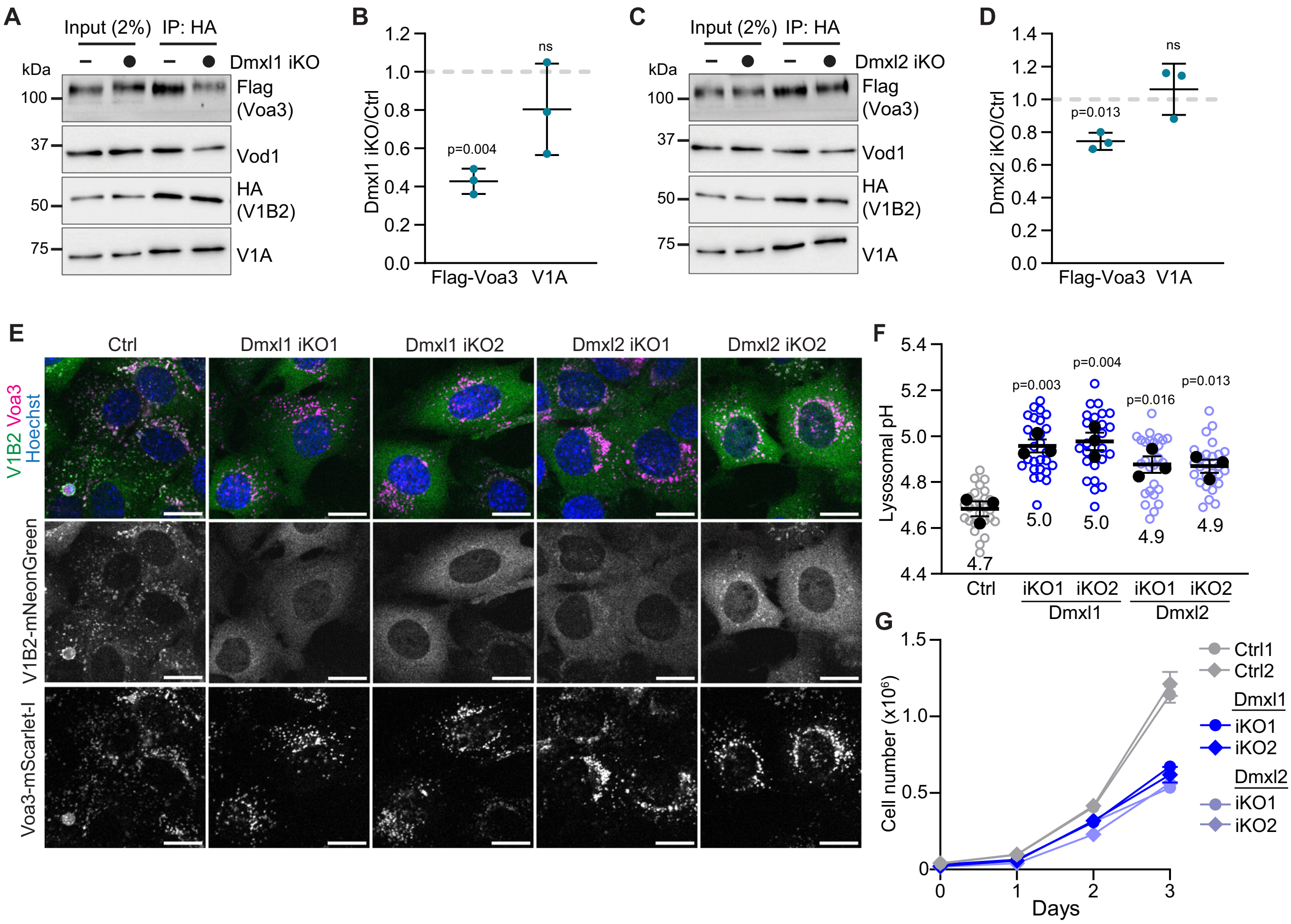

Figure EV2. Dmxl1 and Dmxl2 have non-redundant functions (related to Fig. 1).

(**A**) HA co-IP in Dmxl1 iKO MEFs expressing HA-V1B2 and Flag-Voa3, analyzed by immunoblotting for V-ATPase subunits. (**B**) Quantification of V-ATPase subunit abundance in co-IP immunoblots as in (**A**). (**C**) HA co-IP in Dmxl2 iKO MEFs expressing HA-V1B2 and Flag-Voa3, analyzed by immunoblotting for V-ATPase subunits. (**D**) Quantification of V-ATPase subunit abundance in co-IP immunoblots as in (**C**). (**E**) Subcellular localization of V1B2-mNeonGreen and Voa3-mScarlet-I in Dmxl1 iKO and Dmxl2 iKO MEFs, analyzed by live-cell imaging. (**F**) Lysosomal pH in Dmxl1 iKO and Dmxl2 iKO MEFs, quantified by ratiometric imaging of 10 kDa Dextran Oregon Green and Alexa Fluor 647. (**G**) Proliferation of Dmxl1 iKO and Dmxl2 iKO MEFs. (**B**, **D**) Data are mean ± SD ($n = 3$ independent experiments). (**F**) Data are replicate mean ± SEM (closed circles; $n = 3$ independent experiments) and fields of view (open circles; ≥10 per replicate). (**G**) Data are mean ± SD ($n = 3$ technical replicates). p values were calculated using a two-tailed unpaired t-test with Welch correction (**B**, **D**) or a two-tailed unpaired t-test (**F**). Scale bars, 20 µm.

© The Author(s)

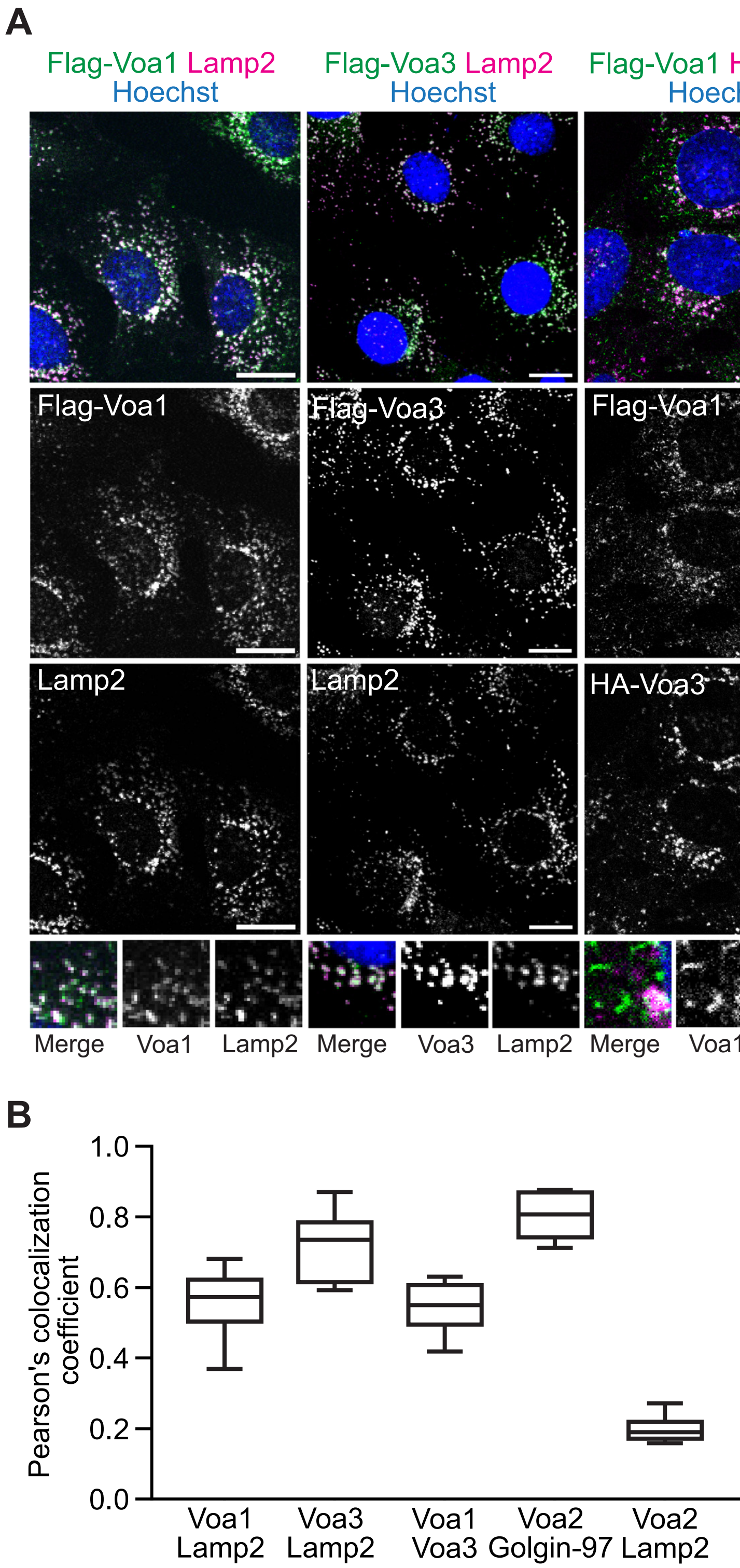

Figure EV3. Subcellular localization of different Voa subunits (related to Fig. 2).

(**A**) Colocalization of Flag-tagged Voa1, Voa2 and Voa3 with endogenous Lamp2 or Golgin-97 in MEFs, analyzed by immunofluorescence (IF). (**B**) Quantification of colocalization of cells shown in (**A**). (**B**) Data are median (center line), 75th and 25th percentiles (upper and lower bound of the box) and min to max range (whiskers) of $n = 10$ fields of views. Scale bars, 20 µm.

 © The Author(s)

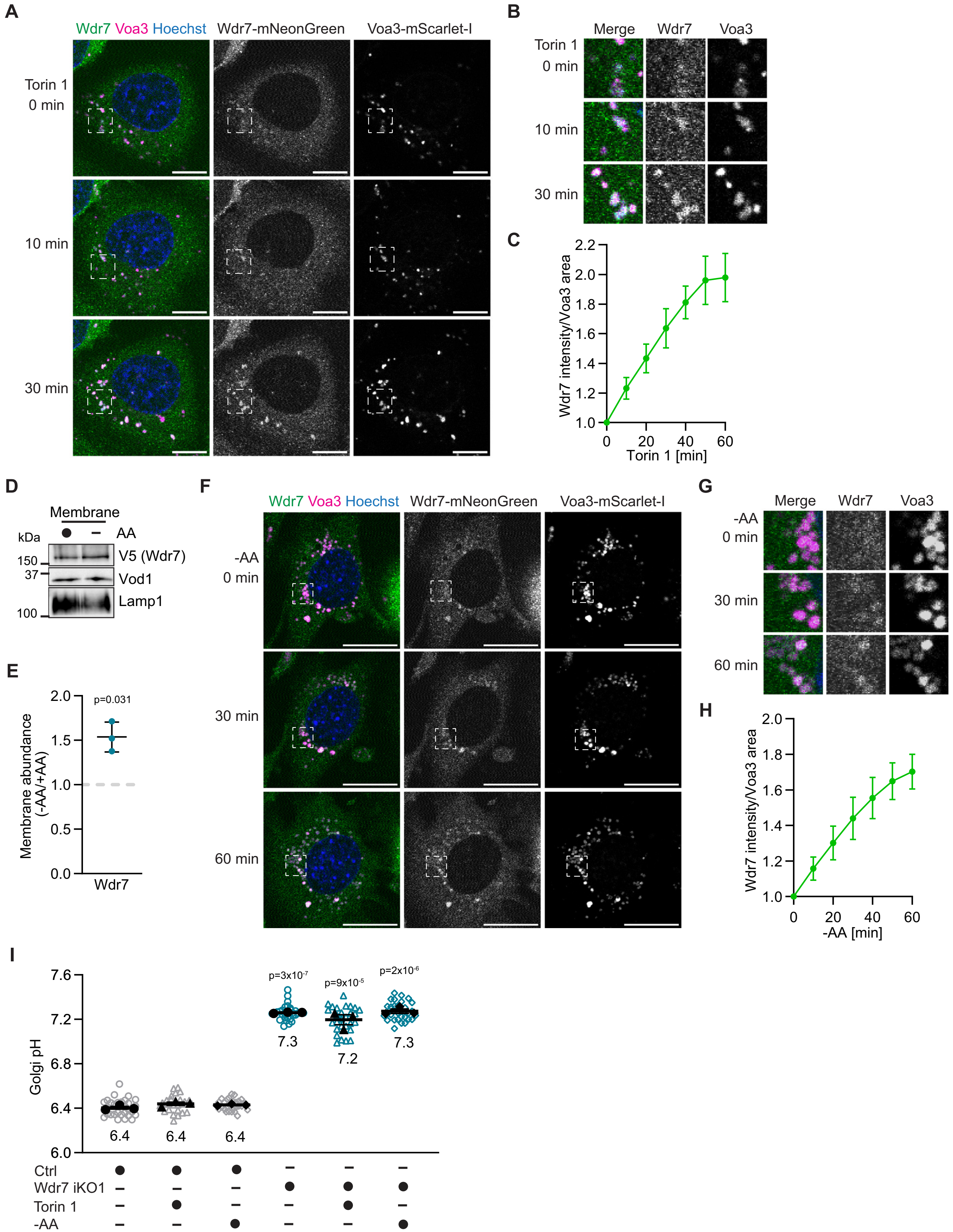
A
Wdr7 Voa3 Hoechst
Wdr7-mNeonGreen
Voa3-mScarlet-I
Torin 1
0 min
10 min
30 min
B
Merge
Wdr7
Voa3
C
Wdr7 intensity/Voa3 area
Torin 1 [min]
D
Membrane
kDa
AA
V5 (Wdr7)
Vod1
Lamp1
150
37
100
E
Membrane abundance (-AA/+AA)
p=0.031
Wdr7
F
-AA
60 min
G
H
-AA [min]
I
Golgi pH
p=3x10-7
p=9x10-5
p=2x10-6
7.3
7.2
6.4
Ctrl
Wdr7 iKO1
Torin 1
-AA

Figure EV4. mRAVE accumulates at lysosomes in response to mTORC1 inactivation (related to Figs. 3 and 4).

(**A**) Subcellular localization of Wdr7-mNeonGreen and Voa3-mScarlet-I in U2OS cells after addition of torin 1 [400 nM], analyzed by live-cell imaging. (**B**) Magnification of areas highlighted in (**A**). (**C**) Change of Wdr7-mNeonGreen intensity in Voa3-mScarlet-I-containing lysosomes, quantified by live-cell imaging as in (**A**). (**D**) V5-Wdr7 abundance in membrane fractions of dextran-loaded lysosomes from MEFs after 1 h ± amino acid starvation, analyzed by immunoblotting. (**E**) Quantification of V5-Wdr7 abundance in immunoblots from membrane fractions as in (**D**). (**F**) Subcellular localization of Wdr7-mNeonGreen and Voa3-mScarlet-I in MEFs after the onset of amino acid starvation, analyzed by live-cell imaging. (**G**) Magnification of areas highlighted in (**F**). (**H**) Change of Wdr7-mNeonGreen intensity in Voa3-mScarlet-I-containing lysosomes, quantified by live-cell imaging as in (**F**). (**I**) Golgi pH in Wdr7 iKO MEFs after 1 h ± torin 1 [400 nM] or amino acid starvation, quantified by ratiometric imaging of MGAT-SEP-mRuby. (**C**, **H**) Data are mean ± SD (≥15,000 organelles in ≥21 fields of view with a total of ≥180 cells). (**E**) Data are mean ± SD ($n = 3$ independent experiments). (**I**) Data are replicate mean ± SEM (closed circles; $n = 3$ independent experiments) and fields of view (open circles; ≥10 per replicate). *p* values were calculated using a two-tailed unpaired *t*-test. Scale bars, 10 µm.

 © The Author(s)

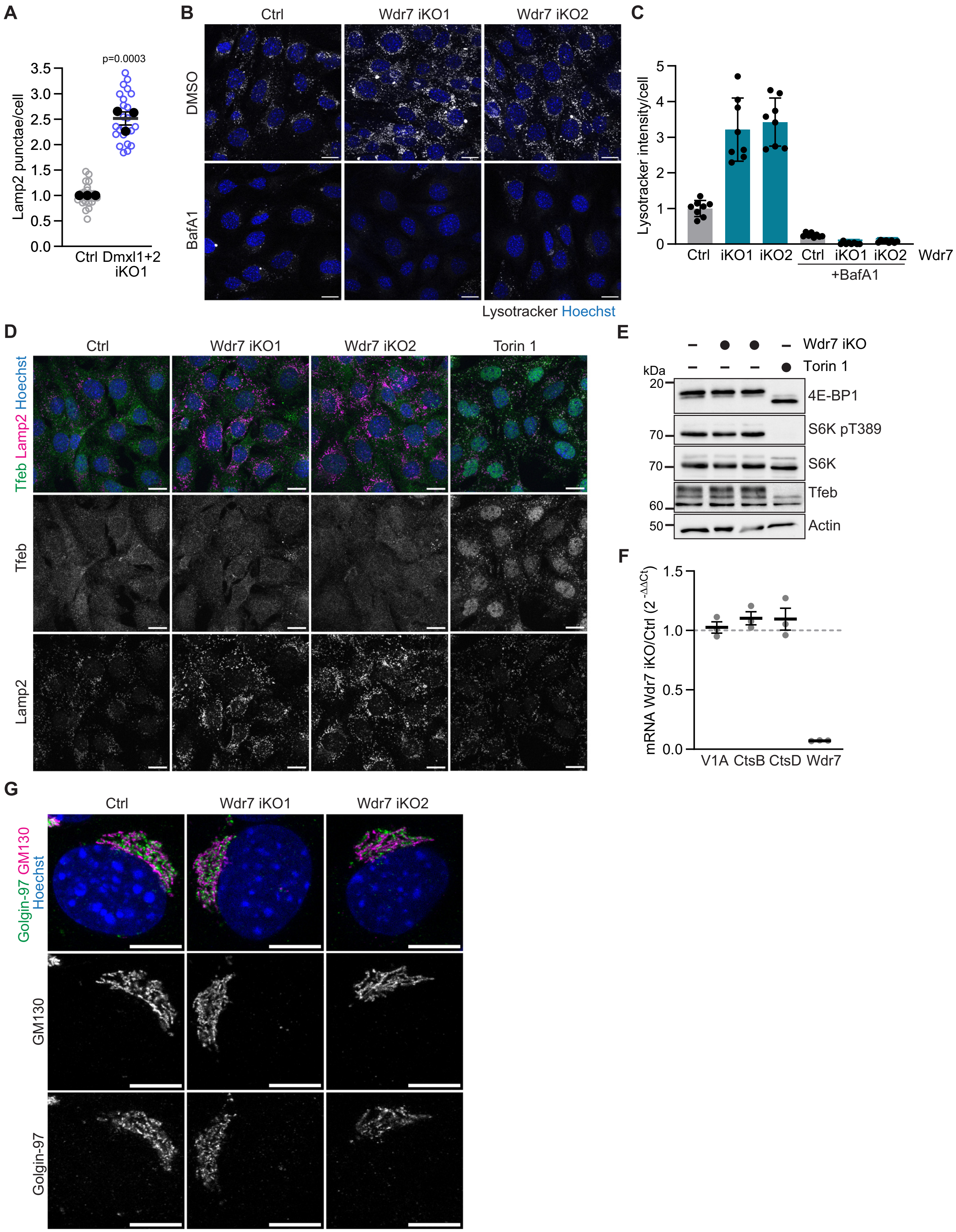
A
p=0.0003
Lamp2 punctae/cell
3.5
3.0
2.5
2.0
1.5
1.0
0.5
0.0
Ctrl Dmxl1+2 iKO1
B
Ctrl
Wdr7 iKO1
Wdr7 iKO2
DMSO
BafA1
Lysotracker Hoechst
C
Lysotracker intensity/cell
5
4
3
2
1
0
Ctrl iKO1 iKO2 Ctrl iKO1 iKO2 Wdr7
+BafA1
D
Ctrl
Wdr7 iKO1
Wdr7 iKO2
Torin 1
Tfeb Lamp2 Hoechst
Tfeb
Lamp2
E
Wdr7 iKO
Torin 1
kDa
20
4E-BP1
70
S6K pT389
70
S6K
60
Tfeb
50
Actin
F
mRNA Wdr7 iKO/Ctrl (2^-ΔΔCt)
1.5
1.0
0.5
0.0
V1A CtsB CtsD Wdr7
G
Ctrl
Wdr7 iKO1
Wdr7 iKO2
Golgin-97 GM130 Hoechst
GM130
Golgin-97

© The Author(s)

Figure EV5. Accumulation of lysosomes in mRAVE-deficient cells is not accompanied by nuclear translocation of Tfeb or changes in Golgi morphology (related to Fig. 5).

(**A**) Quantification of lysosome accumulation in Dmxl1 + Dmxl2 iKO MEFs, assessed by Lamp2 immunofluorescence. (**B**) Lysotracker accumulation in Wdr7 iKO MEFs after 1 h ± bafilomycin A1 [20 nM], analyzed by live-cell imaging. (**C**) Quantification of lysotracker in Wdr7 iKO MEFs as in (**B**). (**D**) Subcellular localization of Tfeb in MEFs ± Wdr7 iKO or 1 h torin 1 [400 nM], analyzed by immunofluorescence. (**E**) mTORC1 signaling activity in MEFs ± Wdr7 iKO or 1 h torin 1 [400 nM], analyzed by phosphorylation of indicated downstream targets. (**F**) Relative mRNA abundance of indicated genes in Wdr7 iKO MEFs, analyzed by RT-qPCR. (**G**) Golgi morphology in Wdr7 iKO MEFs, analyzed by immunofluorescence against the cis-Golgi marker GM130 and the trans-Golgi marker Golgin-97. (**A**) Data are replicate mean ± SEM (closed circles; $n = 3$ independent replicates) and fields of view (open circles; ≥8 per replicate). (**C**) Data are mean ± SD and fields of view ($n = 8$). (**F**) Data are replicate mean ± SEM ($n = 3$ independent replicates). p values were calculated using a two-tailed unpaired t-test with Welch correction. Scale bars, 20 µm (**B**, **D**) or 5 µm (**G**).

© The Author(s)

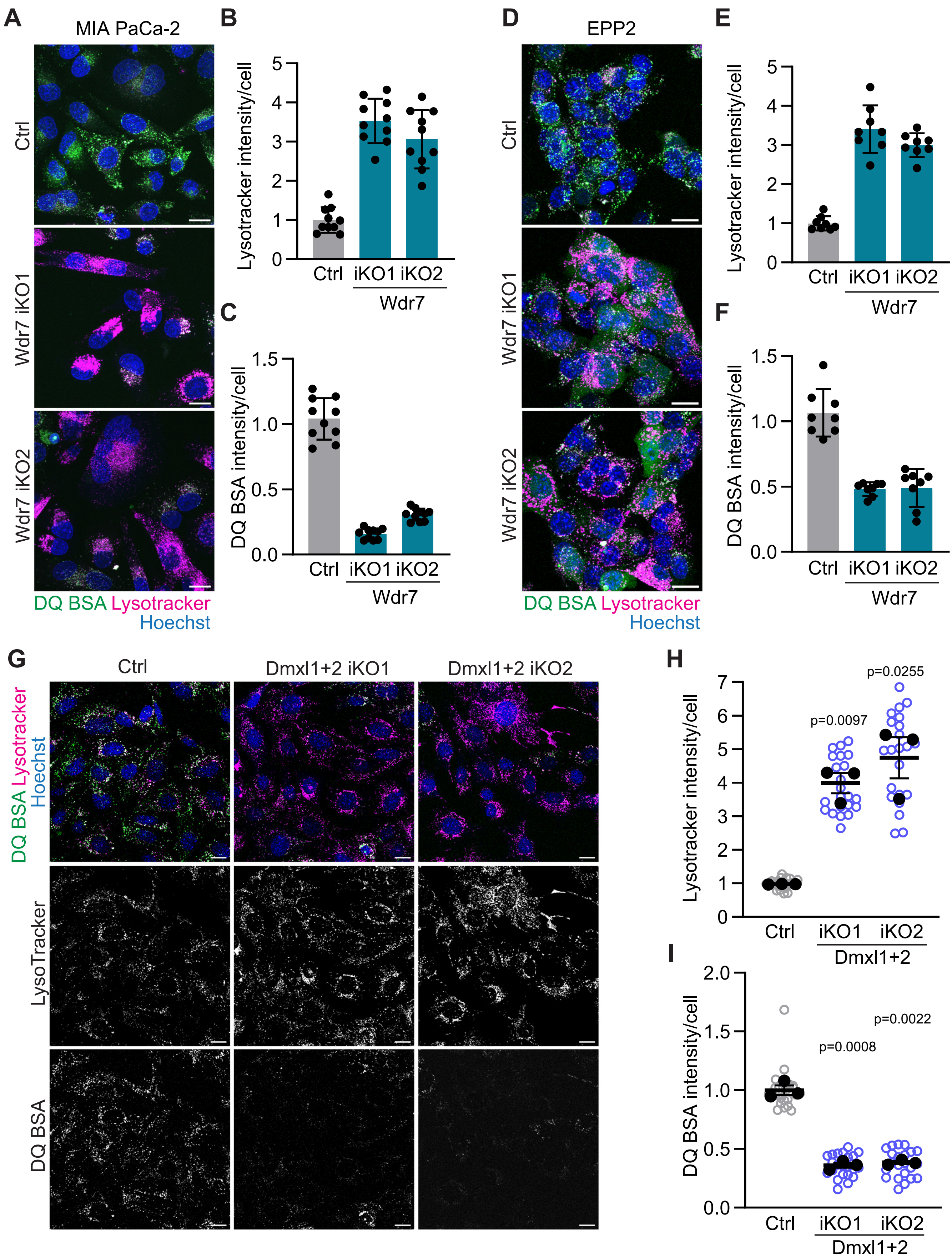
A
MIA PaCa-2
Ctrl
Wdr7 iKO1
Wdr7 iKO2
DQ BSA Lysotracker
Hoechst
B
Lysotracker intensity/cell
Ctrl iKO1 iKO2
Wdr7
C
DQ BSA intensity/cell
Ctrl iKO1 iKO2
Wdr7
D
EPP2
Ctrl
Wdr7 iKO1
Wdr7 iKO2
DQ BSA Lysotracker
Hoechst
E
Lysotracker intensity/cell
Ctrl iKO1 iKO2
Wdr7
F
DQ BSA intensity/cell
Ctrl iKO1 iKO2
Wdr7
G
Ctrl
Dmxl1+2 iKO1
Dmxl1+2 iKO2
DQ BSA Lysotracker
Hoechst
LysoTracker
DQ BSA
H
Lysotracker intensity/cell
p=0.0097
p=0.0255
Ctrl iKO1 iKO2
Dmxl1+2
I
DQ BSA intensity/cell
p=0.0008
p=0.0022
Ctrl iKO1 iKO2
Dmxl1+2

Figure EV6. Loss of Wdr7 suppresses lysosomal catabolism (related to Fig. 5).

(**A**) Lysotracker accumulation and DQ BSA degradation in Wdr7 iKO MIA PaCa-2 cells, analyzed by live-cell imaging. (**B**, **C**) Quantification of lysotracker (**B**) and DQ BSA (**C**) in Wdr7 iKO MIA PaCa-2 cells, as shown in (**A**). (**D**) Lysotracker accumulation and DQ BSA degradation in Wdr7 iKO EPP2 cells, analyzed by live-cell imaging. (**E**, **F**) Quantification of lysotracker (**E**) and DQ BSA (**F**) in Wdr7 iKO EPP2 cells, as shown in (**D**). (**G**) Lysotracker accumulation and DQ BSA degradation in Dmxl1 + Dmxl2 iKO MEFs, analyzed by live-cell imaging. (**H**, **I**) Quantification of lysotracker (**H**) and DQ BSA (**I**) in Dmxl1 + Dmxl2 iKO MEFs, as shown in (**G**). (**B**, **C**, **E**, **F**) Data were mean ± SD and fields of view ($n = 8$–10). (**H**, **I**) Data are replicate mean ± SEM (closed circles; $n = 3$ independent replicates) and fields of view (open circles; ≥8 per replicate). p values were calculated using a two-tailed unpaired t-test with Welch correction. Scale bars, 20 µm.

 © The Author(s)

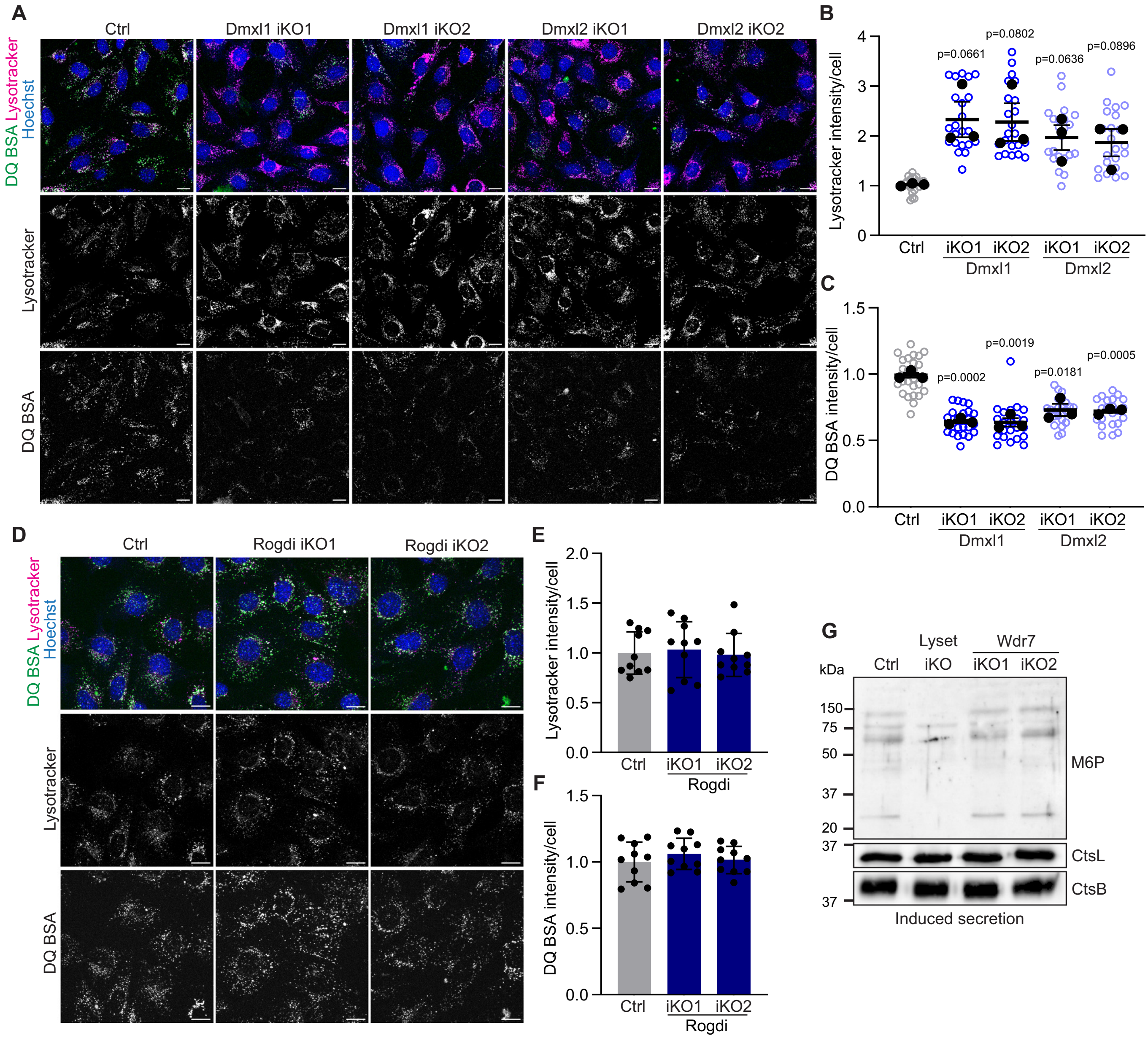

Figure EV7. Lysosomal phenotypes in Dmxl1, Dmxl2 or Rogdi iKO cells (related to Fig. 5).

(**A**) Lysotracker accumulation and DQ BSA degradation in Dmxl1 iKO and Dmxl2 iKO MEFs, analyzed by live-cell imaging. (**B**, **C**) Quantification of lysotracker (**B**) and DQ BSA (**C**) in Dmxl1 iKO and Dmxl2 iKO MEFs, as shown in (**A**). (**D**) Lysotracker accumulation and DQ BSA degradation in Rogdi iKO MEFs, analyzed by live-cell imaging. (**E**, **F**) Quantification of lysotracker (**E**) and DQ BSA (**F**) in Rogdi iKO MEFs, as shown in (**D**). (**G**) M6P modification of newly synthesized proteins under induced secretion conditions (10 mM NH_4Cl, 24 h) in Wdr7 iKO and Lyset iKO MEFs, analyzed by immunoblotting. (**B**, **C**) Data are replicate mean ± SEM (closed circles; $n = 3$ independent replicates) and fields of view (open circles; ≥7 per replicate). (**E**, **F**) Data are mean ± SD and fields of view ($n = 10$). *p* values were calculated using a two-tailed unpaired *t*-test with Welch correction. Scale bars, 20 µm.

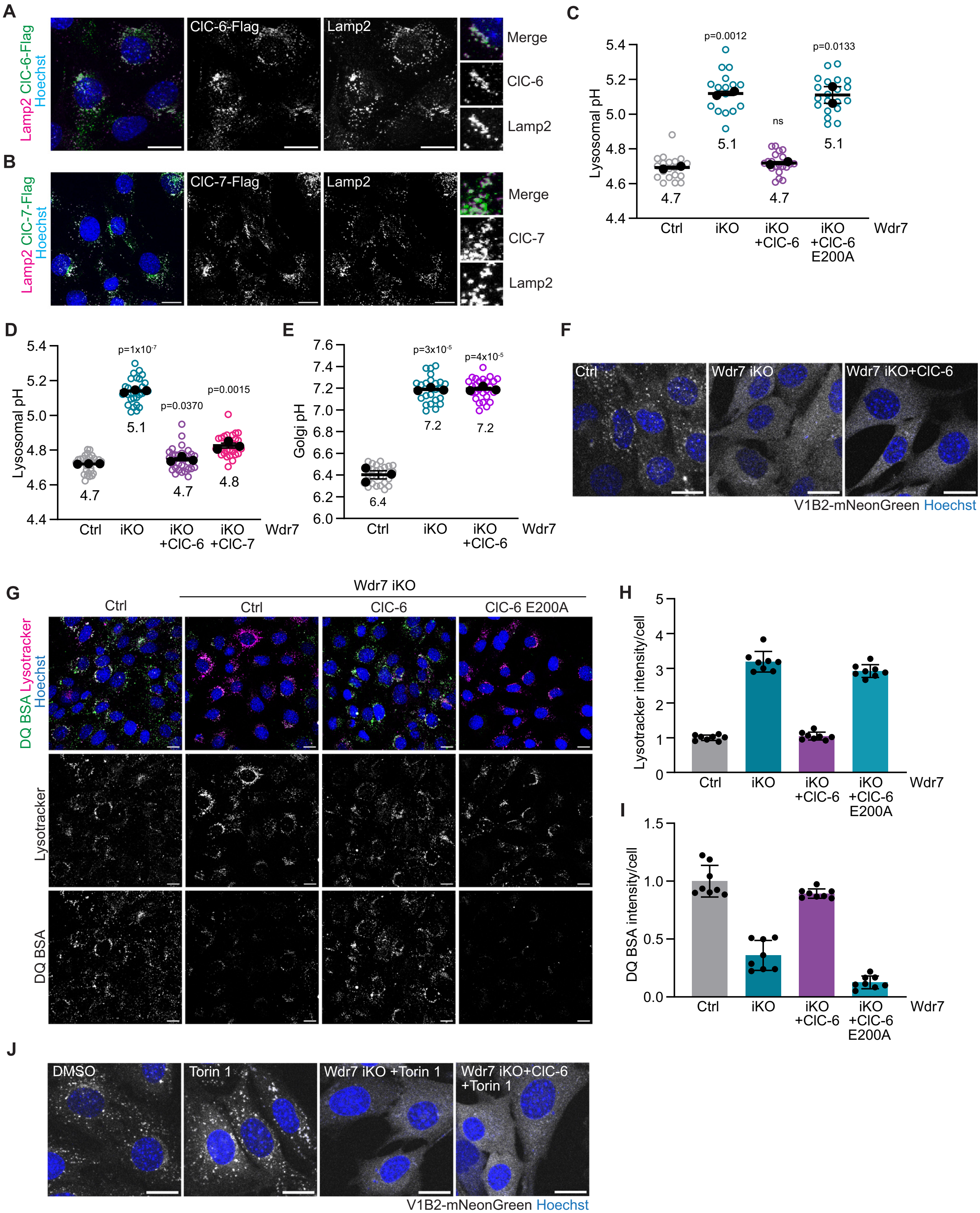
A
Lamp2 ClC-6-Flag Hoechst
ClC-6-Flag
Lamp2
Merge
ClC-6
Lamp2
B
Lamp2 ClC-7-Flag Hoechst
ClC-7-Flag
Lamp2
Merge
ClC-7
Lamp2
C
Lysosomal pH
p=0.0012
p=0.0133
ns
4.7
5.1
4.7
5.1
Ctrl
iKO
iKO +ClC-6
iKO +ClC-6 E200A
Wdr7
D
Lysosomal pH
p=1x10-7
p=0.0370
p=0.0015
4.7
5.1
4.7
4.8
Ctrl
iKO
iKO +ClC-6
iKO +ClC-7
Wdr7
E
Golgi pH
p=3x10-5
p=4x10-5
6.4
7.2
7.2
Ctrl
iKO
iKO +ClC-6
Wdr7
F
Ctrl
Wdr7 iKO
Wdr7 iKO+ClC-6
V1B2-mNeonGreen Hoechst
G
Ctrl
Wdr7 iKO
Ctrl
ClC-6
ClC-6 E200A
DQ BSA Lysotracker Hoechst
Lysotracker
DQ BSA
H
Lysotracker intensity/cell
Ctrl
iKO
iKO +ClC-6
iKO +ClC-6 E200A
Wdr7
I
DQ BSA intensity/cell
Ctrl
iKO
iKO +ClC-6
iKO +ClC-6 E200A
Wdr7
J
DMSO
Torin 1
Wdr7 iKO +Torin 1
Wdr7 iKO+ClC-6 +Torin 1
V1B2-mNeonGreen Hoechst

© The Author(s)

Figure EV8. The Cl^-/H^+ antiporters ClC-6 and ClC-7 restore lysosomal defects of Wdr7 iKO MEFs (related to Fig. 7).

(**A**, **B**) Colocalization of ClC-6-Flag (**A**) or ClC-7-Flag (**B**) with endogenous Lamp2 in MEFs, analyzed by immunofluorescence. (**C**) Lysosomal pH in Wdr7 iKO MEFs ± expression of ClC-6 or mutant ClC-6^{E200A}, which is deficient for proton transport but retains chloride conductance (Neagoe et al, 2010), quantified by ratiometric imaging of 10 kDa Dextran Oregon Green and Alexa Fluor 647. (**D**) Lysosomal pH in Wdr7 iKO MEFs ± ClC-6 or ClC-7 expression, quantified by ratiometric imaging of 10 kDa Dextran Oregon Green and Alexa Fluor 647. (**E**) Golgi pH in Wdr7 iKO MEFs ± ClC-6 expression, quantified by ratiometric imaging of MGAT-SEP-mRuby. (**F**) Subcellular localization of V1B2-mNeonGreen in Wdr7 iKO MEFs ± ClC-6 expression, analyzed by live-cell imaging. (**G**) Lysotracker accumulation and DQ BSA degradation in Wdr7 iKO MEFs ± expression of ClC-6 or proton transport-deficient ClC-6^{E200A}, analyzed by live-cell imaging. (**H**, **I**) Quantification of lysotracker (**H**) and DQ BSA (**I**) in Wdr7 iKO MEFs ± expression of ClC-6 or proton transport-deficient ClC-6^{E200A} as shown in (**G**). (**J**) Subcellular localization of V1B2-mNeonGreen in Wdr7 iKO MEFs after 1 h ± torin 1 [400 nM], analyzed by live-cell imaging. (**C–E**) Data are replicate mean ± SEM (closed circles; $n = 2$–3 independent experiments) and fields of view (open circles; ≥8 per replicate). (**H**, **I**) Data are mean ± SD and fields of view ($n = 8$). p values were calculated using a two-tailed unpaired t-test. Scale bars, 20 μm.

© The Author(s)
