# Supplementary material for: mRAVE governs lysosomal catabolism through basal and mTORC1-regulated V-ATPase assembly
Source: EMBO J. 2026 Jun 22;45(17):5988–6020. doi: 10.1038/s44318-026-00838-5 (PMC13534531; doi:10.1038/s44318-026-00838-5)
Supplement: Supplementary file 2 [file 44318_2026_838_MOESM1_ESM.pdf]

Appendix

mRAVE governs lysosomal catabolism through basal and mTORC1-regulated V-ATPase assembly

Nora S. Siefert, Andrea Zanotti, Anastasija Paneva, Martin Schneider, Dominic Helm, Wilhelm Palm

Table of Contents

Appendix Table S1 sgRNA sequences 2
Appendix Table S2 PCR primers for validation of CRISPR/Cas9 editing 2
Appendix Table S3 RT-qPCR primers 2
Appendix Table S4 PCR primers for cDNA cloning 3

Appendix Table S1 sgRNA sequences

Gene	Species	sgRNA sequence 1	sgRNA sequence 2
Dmxl1 dsg1	mouse	AGTTGGGGAAAGTACATGAG	TGATGGTGAGAGATCTAAGG
Dmxl1 dsg2	mouse	AGTAGGGAGCCATGCCACAT	GCATTAAGGACACCAAATGT
Dmxl2 dsg1	mouse	GTGGGACACAGTCAGAACAG	TATATGACAAAGGTCCAATG
Dmxl2 dsg2	mouse	GGTAAGAGATGGAATATTGG	TTGAATGCGAATCTACAGGA
Wdr7 dsg1	mouse	GAGGCCTTCCGACGACTACCTGG	GTAGCCAGTGACCACTCTGTAGG
Wdr7 dsg2	mouse	GGAATACAACACCTCAAGGCTGG	TTGGGAACCAGCAAGAGGGCAGG
Chr1.9 dsg1	mouse/ human	GACAATGAACATAAGCACAT	GTTTGGCCTGAAATCCCACC
CD19 dsg1	mouse/ human	CACCGCCGCTTTGGGACCACAGAA	GCCTAAAGCCCCTTGTGCT
Rogdi dsg1	mouse	AAGCAGGAGAACTTCATCCTGGG	GCTGGCAGCAATCTCCGGCAGGG
Rogdi dsg2	mouse	AGGGGATGCCCTCAGCCAAGCGG	CGTGCAGCTGGTATACAGTGAGG
Vamp7 dsg1	mouse	GCACAAGTGGATGAACTGAA	GGTGTGGAGGAAACTTCC
Vamp7 dsg2	mouse	GATAAAACAGAAAACCTTG	GTGTGGAGGAAACTTCCTGG
Syt7 dsg1	mouse	GAGGACGATAGTGACGCTA	GCCACTGGTGTCAGCGCAAAC
Syt7 dsg2	mouse	GATGAGTCTGACCGCAGAA	GCCCACTTCCAGGTTGCCTGC
Wdr7 dsg1	human	TGGTGGATCCAACTACTCGC	GGAGTAATAGGAGCTGAATT
Wdr7 dsg2	human	TGTGTGTTCCAAATATTGGA	GTGGTAGCACTCTCGGTGAC

Appendix Table S2 PCR primers for validation of CRISPR/Cas9 editing

Gene	Forward primer	Reverse primer
Dmxl1 dsg1	AGTTTTGCTGAGGAATCTGCTTT	GAAATTTCAGGGTTGGAAAGTTCAT
Dmxl1 dsg2	AGTAACTCATCAAGTTCTGGGCT	TTCTTTAAGGAACTCTCACTCCCA
Dmxl2 dsg1	CCCATCCCAGCAAGAGATGAT	TGGAAGCCATGCCACATTAG
Dmxl2 dsg2	GCAACCCATCCACCACAATG	CCAGCATAGAGGATTTAAGGGC
Wdr7 dsg1	CCAGTCTTTAGTCTTTGCA	GCTACACATGCCATCAT
Wdr7 dsg2	GGACTCTGATGGTAGAAG	GGCAGGTCACAAATTACT
Rogdi dsg1	GATGTTGCTTGCCGATGCTA	CAGAAACAGCAGAGTCTGTG
Rogdi dsg2	CAGGACTGGAATGAACGGGT	GCTAGCTGGTGGCAGAGCTA

Appendix Table S3 RT-qPCR primers

Gene	Species	Forward primer	Reverse primer
Atp6v1a	mouse	CTACCCAAAATCCGCGATGAG	CCATGTCACCTTCCAATCGAA
CtsB	mouse	TCCTTGATCCTTCTTTCTTGCC	ACAGTGCCACACAGCTTCTTC
CtsD	mouse	GCTTCCGGTCTTTGACAACCT	CACCAAGCATTAGTTCTCCTCC
Tbp	mouse	CCTTGTACCCTTCACCAATGAC	ACAGCCAAGATTCACGGTAGA
Gapdh	mouse	TCACCACCATGGAGAAGGC	GCTAAGCAGTTGGTGGTGCA

Appendix Table S4 PCR primers for cDNA cloning

Gene	Forward primer	Reverse primer
pLV-UbC-3xFLAG-Atp6v0a1	tttggcttttttgttagacgatggactacaaagaccatgacggtgattataaagatcatgatatcgattacaaggatgacgatgacaaggggggagcttttccggagtg	tagagcggccgccctcgaggTCACTCATCAAACTTCCC
pLV-UbC-3xFLAG-Atp6v0a1-mScarlet-I	tttggcttttttgttagacgatggactacaaagaccatgacggtgattataaagatcatgatatcgattacaaggatgacgatgacaaggggggagcttttccggagtg	ccttgctcacggatccgcctccgccctcatcaaacttcccttc
	gtttgatgagggcggaggcggatccgtgagcaagggcgaggca	tagagcggccgccctcgaggTCACTTGTACAGCTCGTCCATG
pLV-UbC-3xFLAG-Atp6v0a2	tttggcttttttgttagacgatggactacaaagaccatgacggtgattataaagatcatgatatcgattacaaggatgacgatgacaagggctctctcttccgcagc	tagagcggccgccctcgaggTCAGGATCCGCCTCCGCC
pLV-UbC-3xFLAG-Atp6v0a2-mScarlet-I	tttggcttttttgttagacgatggactacaaagaccatgacggtgattataaagatcatgatatcgattacaaggatgacgatgacaagggctctctcttccgcagc	cgcctccgccggatccgcctccgcctgctatgctgtcatcattactgaac
	cagcatagcaggcggaggcggatccggcggaggcggatccgtg	tagagcggccgccctcgaggTCACTTGTACAGCTCGTCCATG
pLV-UbC-3xFLAG-Atp6v0a3	tttggcttttttgttagacgatggactacaaagaccatgacggtgattataaagatcatgatatcgattacaaggatgacgatgacaagggctctatgttccggagtg	tagagcggccgccctcgaggTCAGTCACTGTCCACAGTG
pLV-UbC-3xFLAG-Atp6v0a3-mScarlet-I	tttggcttttttgttagacgatggactacaaagaccatgacggtgattataaagatcatgatatcgattacaaggatgacgatgacaagggctctatgttccggagtg	ccttgctcacggatccgcctccgccgtcactgtccacagtgaag
	ggacagtgacggcggaggcggatccgtgagcaagggcgaggca	tagagcggccgccctcgaggTCACTTGTACAGCTCGTCCATG
pLV-UbC-HA-Atp6v1b2	tttggcttttttgttagacgatgtacccatacgacgtc	tagagcggccgccctcgaggtcagtgttttgcagagtctcg
pRRL-UbC-HA-Atp6v1b2-mNeonGreen	ttggcttttttgttagacggatgtacccatacgacgtcccagactacgctgcgttgcgagcgatgcgg	ctcacagatccgcctccttggtgttttgcagagtctcgagggtaaaattc
	tgcaaaacacggcggaggcggatccgtgagcaagggcgagg	ccttgctcacggatccgcctccgccgtgttttgcagagtctcgagggtaaaattc
pLV-UbC-V5-Wdr7	tttggcttttttgttagacgatgggtaaaccaatcccc	tagagcggccgccctcgaggtcagaccatgaagcggtg
pLV-UbC-V5-Wdr7 dsg resistant	GTTGTATTCCttaGTATCGAAGATC	ACCTCAAGGCTGGTG
	GCAAGAGGGCagaCTCCTCTGCC	TGGTTCCCAACAGAGAACTGG
UbC-HA-Wdr7-NeonGreen	tttggcttttttgttagacgatgtacccatacgacgtcccagactacgctgcaggaaacagcctagttc	cttcatggtcggcggaggcggatcggtg
	cgcctccgccgaccatgaagcggtgttc	tagagcggccgccctcgaggTCACTTGTACAGCTCGTCCATG
pRRL-UbC-HA-Atp6v1b2-mScarlet-I	ttggcttttttgttagacggatgtacccatacgacgtcccagactacgctgcgttgcgagcgatgcgg	ctcacagatccgcctccttggtgttttgcagagtctcgagggtaaaattc
	tgcaaaacacggcggaggcggatccgtgagcaagggcgagg	ccttgctcacggatccgcctccgccgtgttttgcagagtctcgagggtaaaattc
pLV-UbC-hClCN6-FLAG	tttggcttttttgttagacgATGGCGGGGTGCAGGGGG	tggtctttgtagtcgcttccGATGGTCTGGTAGTGCTGCCTCAGC
pLV-UbC-hClCN6-FLAG E200A	CGTGGAGAAGgcgGGCCCCATGA	AAGAGCCCTCCAGCC
pLV-UbC-hClCN7-Flag-P2A-Ostm1-V5	tttggcttttttgttagacgatggccaacgtctctaagaaagtg	cttgtcatcgtcgtccttgtagtccgtctgggccagcgaaag
	gactacaaggacgacgatgacaaggctagcggcagcggcgcc	cccgagccatggggccggggttctcctc
	ccccggccccatggctcgggacgcggag	tagagcggccgccctcgaggtcatgtgctgtccagtcccagcagtgggttggggattggtttaccggtggcattttcttgaatgttggcaaaa
