# Supplementary material for: mRAVE governs lysosomal catabolism through basal and mTORC1-regulated V-ATPase assembly
Source: EMBO J. 2026 Jun 22;45(17):5988–6020. doi: 10.1038/s44318-026-00838-5 (PMC13534531; doi:10.1038/s44318-026-00838-5)
Supplement: Supplementary file 3 [file 44318_2026_838_MOESM2_ESM.pdf]

THE EMBO JOURNAL

mRAVE governs lysosomal catabolism through basal and mTORC1-regulated V-ATPase assembly

Nora Siefert, Andrea Zanotti, Anastasija Paneva, Martin Schneider, Dominic Helm, and Wilhelm Palm

Corresponding author(s): Wilhelm Palm (w.palm@dkfz-heidelberg.de)

Review Timeline:

Submission Date:	9th Dec 25
Editorial Decision:	2nd Feb 26
Revision Received:	15th Apr 26
Editorial Decision:	11th May 26
Revision Received:	18th May 26
Accepted:	22nd May 26

Editor: William Teale

Transaction Report:

(Note: With the exception of the correction of typographical or spelling errors that could be a source of ambiguity, letters and reports are not edited. Depending on transfer agreements, referee reports obtained elsewhere may or may not be included in this compilation. Referee reports are anonymous unless the Referee chooses to sign their reports.)

1st Editorial Decision **2nd Feb 2026**

Dear Wilhelm,

Thank you again for the submission of your manuscript entitled "Mammalian RAVE couples V-ATPase assembly to organelle acidification and function" and for your patience during the review process. We have now received reports from three referees, which I copy below.

As you can see from their comments, all thought that your manuscript could represent a useful and timely development to our understanding lysosome function in the context of the cell's nutrient status. That said, all of them point out potential contradictions that may need additional experimentation to resolve (for example on the translocation of Wdr7 upon torin 1 treatment). This will require your attention before your manuscript can be published in The EMBO Journal.

Based on the overall interest expressed in the reports, however, I would like to invite you to address the comments of all referees in a revised version of the manuscript. I should add that it is The EMBO Journal policy to allow only a single major round of revision and that it is therefore important to resolve the main concerns at this stage. I believe the concerns of the referees are reasonable and addressable, but please contact me if you have any questions (I am always available for a chat [over Zoom or even a coffee] about the revision plan), need further input on the referee comments or if you anticipate any problems in addressing any of their points. Please, follow the instructions below when preparing your manuscript for resubmission.

I would also like to point out that as a matter of policy, competing manuscripts published during this period will not be taken into consideration in our assessment of the novelty presented by your study ("scooping" protection). We have extended this 'scooping protection policy' beyond the usual 3 month revision timeline to cover the period required for a full revision to address the essential experimental issues. Please contact me if you see a paper with related content published elsewhere to discuss the appropriate course of action.

When preparing your letter of response to the referees' comments, please bear in mind that this will form part of the Review Process File, and will therefore be available online to the community. For more details on our Transparent Editorial Process, please visit our website: https://link.springer.com/journal/44318/submission-guidelines#cms-Revised-submissions

Again, please contact me at any time during revision if you need any help or have further questions.

Thank you very much again for the opportunity to consider your work for publication. I look forward to your revision.

Best regards,

William

William Teale, Ph.D.
Editor
The EMBO Journal

When submitting your revised manuscript, please carefully review the instructions below and include the following items:

1) a .docx formatted version of the manuscript text (including legends for main figures, EV figures and tables). Please make sure that the changes are highlighted to be clearly visible.

2) individual production quality figure files as .eps, .tif, .jpg (one file per figure).

3) a .docx formatted letter INCLUDING the reviewers' reports and your detailed point-by-point response to their comments. As part of the EMBO Press transparent editorial process, the point-by-point response is part of the Review Process File (RPF), which will be published alongside your paper.

4) a complete author checklist, which you can download from our author guidelines (https://link.springer.com/journal/44318/submission-guidelines#cms-Revised-submissions). Please insert information in the checklist that is also reflected in the manuscript. The completed author checklist will also be part of the RPF.

5) Please note that all corresponding authors are required to supply an ORCID ID for their name upon submission of a revised manuscript.

6) We require a 'Data Availability' section after the Materials and Methods. Before submitting your revision, primary datasets

produced in this study need to be deposited in an appropriate public database, and the accession numbers and database listed under 'Data Availability'. Please remember to provide a reviewer password if the datasets are not yet public (see https://link.springer.com/partners/embo-press/editorial-policies#Data%20deposition). If no data deposition in external databases is needed for this paper, please then state in this section: This study includes no data deposited in external repositories. Note that the Data Availability Section is restricted to new primary data that are part of this study.

Note - All links should resolve to a page where the data can be accessed.

7) When assembling figures, please refer to our figure preparation guideline in order to ensure proper formatting and readability in print as well as on screen:
https://link.springer.com/journal/44318/submission-guidelines#cms-Figure-and-data-presentation

Please remember: Digital image enhancement is acceptable practice, as long as it accurately represents the original data and conforms to community standards. If a figure has been subjected to significant electronic manipulation, this must be noted in the figure legend or in the 'Materials and Methods' section. The editors reserve the right to request original versions of figures and the original images that were used to assemble the figure.

8) For data quantification: please specify the name of the statistical test used to generate error bars and P values, the number (n) of independent experiments (specify technical or biological replicates) underlying each data point and the test used to calculate p-values in each figure legend. The figure legends should contain a basic description of n, P and the test applied. Graphs must include a description of the bars and the error bars (s.d., s.e.m.).

9) We would also encourage you to include the source data for figure panels that show essential data. Numerical data can be provided as individual .xls or .csv files (including a tab describing the data). For 'blots' or microscopy, uncropped images should be submitted (using a zip archive or a single pdf per main figure if multiple images need to be supplied for one panel). Additional information on source data and instruction on how to label the files are available at
https://link.springer.com/journal/44318/submission-guidelines#cms-Source-data

10) We replaced Supplementary Information with Expanded View (EV) Figures and Tables that are collapsible/expandable online (see examples in https://www.embopress.org/doi/10.15252/embj.201695874). A maximum of 5 EV Figures can be typeset. EV Figures should be cited as 'Figure EV1, Figure EV2" etc. in the text and their respective legends should be included in the main text after the legends of regular figures.

- For the figures that you do NOT wish to display as Expanded View figures, they should be bundled together with their legends in a single PDF file called *Appendix*, which should start with a short Table of Content. Appendix figures should be referred to in the main text as: "Appendix Figure S1, Appendix Figure S2" etc. See detailed instructions regarding expanded view here: < https://link.springer.com/journal/44318/submission-guidelines#cms-Expanded-View-data >.

- Additional Tables/Datasets should be labelled and referred to as Table EV1, Dataset EV1, etc. Legends have to be provided in a separate tab in case of .xls files. Alternatively, the legend can be supplied as a separate text file (README) and zipped together with the Table/Dataset file.

11) At EMBO Press we ask authors to provide source data for the main manuscript figures. Our source data coordinator will contact you to discuss which figure panels we would need source data for and will also provide you with helpful tips on how to upload and organize the files.

12) Our journal encourages inclusion of *data citations in the reference list* to directly cite datasets that were re-used and obtained from public databases. Data citations in the article text are distinct from normal bibliographical citations and should directly link to the database records from which the data can be accessed. In the main text, data citations are formatted as follows: "Data ref: Smith et al, 2001" or "Data ref: NCBI Sequence Read Archive PRJNA342805, 2017". In the Reference list, data citations must be labeled with "[DATASET]". A data reference must provide the database name, accession number/identifiers and a resolvable link to the landing page from which the data can be accessed at the end of the reference. Further instructions are available at < https://link.springer.com/journal/44318/submission-guidelines#cms-Reference-guidelines >.

13) In order to increase the reproducibility and reach of your work, The EMBO Journal includes a table of reagents that were used in the study. Please provide this along with your revisions.

Further instructions for preparing your revised manuscript:

Read our guidance for manuscript revisions and related editorial policies: https://link.springer.com/journal/44318/submission-guidelines#cms-Revised-submissions

Please make sure you upload a letter of response to the referees' comments together with the revised manuscript.

Please also check that the title and abstract of the manuscript are brief, yet explicit, even to non-specialists.

When assembling figures, please refer to our figure preparation guideline in order to ensure proper formatting and readability in print as well as on screen:
https://media.springernature.com/original/springer-cms/rest/v1/content/27825798/data/v1

At EMBO Press we ask authors to provide source data for the main manuscript figures. You will receive a separate email with instructions for providing source data with your revised manuscript, including how to upload and organize the files.

IMPORTANT: When you send the revision we will require
- a point-by-point response to the referees' comments, with a detailed description of the changes made (as a word file).
- a word file of the manuscript text.
- individual production quality figure files (one file per figure)
- a complete author checklist
- Expanded View files (replacing Supplementary Information)
- a Reagents and Tools Table as part of the Methods section

Please remember: Digital image enhancement is acceptable practice, as long as it accurately represents the original data and conforms to community standards. If a figure has been subjected to significant electronic manipulation, this must be noted in the figure legend or in the 'Materials and Methods' section. The editors reserve the right to request original versions of figures and the original images that were used to assemble the figure.

We realize that it is difficult to revise to a specific deadline. In the interest of protecting the conceptual advance provided by the work, we recommend a revision within 3 months (3rd May 2026). Please discuss the revision progress ahead of this time with the editor if you require more time to complete the revisions. Use the link below to submit your revision:

https://emboj.msubmit.net/cgi-bin/main.plex

Referee #1:

In this manuscript, the authors assess the role of V-ATPase assembly factor mRAVE in the function of the Golgi apparatus and lysosomes in mammalian cells (MEFs). Although a related RAVE complex has been described in yeast, the roles of the mRAVE complex (previously known as Rabconnectin-3) have not been thoroughly described in mammals. The experiments are well-done overall and significantly extend the authors' previous work showing that V-ATPase assembly at lysosomes is promoted by inhibition of mTORC1. They show that acidification of the Golgi is very sensitive to loss of mRAVE function which is a novel and important result. There is a more modest increase in lysosomal pH in mRAVE-deficient cells, but this increase appears to be sufficient to significantly impact lysosomal function. However, there are still questions that should be addressed before publication.
1). Golgi pH increases from 6.4 to 7.3 in the induced KO strains of mRAVE subunits Wdr7 or Dmxl1 and 2. This is an important result, since in yeast the Golgi V-ATPase does not appear to be dependent on RAVE for assembly or function. However, the authors do not consider the impact that the loss of Golgi acidification could have on other processes such as sorting of lysosomal proteins. For example, there is increased secretion of lysosomal enzymes that is attributed to lysosomal exocytosis, but it could also arise from missorting of lysosomal proteins to the cell surface from the Golgi. This possibility should be addressed.
2). In contrast to the Golgi, the authors see a relatively small, though statistically significant, increase in lysosomal pH from 4.7 to 5.1-5.2 in the induced KO strains. Treatment with the V-ATPase inhibitor bafilomycin further increases the pH to 6.3 in these strains, suggesting there is significant residual V-ATPase activity at the lysosomes in the KO strains. The authors focus on the alterations in lysosomal function that seem to accompany the small rise in pH in mRAVE-deficient cells, but the bafilomycin result calls into question the conclusion that mRAVE is the "principle assembly factor" (Discussion). It should be addressed whether the active V-ATPases that remain in the KO strains are pre-existing complexes or whether there are pathways to V-ATPase assembly at the vacuole that are RAVE-independent. Alternatively, the WDR7 and DMXL1/2 KO may not be complete. These are important distinctions to be addressed
3). The ability of Clc-6 expression to restore lysosomal pH and multiple lysosomal phenotypes in the Wdr7-deficient cells is interesting. It is likely that Cl- transport into the vacuole through Clc-6 antiporter is promoting activity of the residual active V-ATPases in the mRAVE-deficient cells by lowering the lysosomal membrane potential. The Clc-6 antiporter "pumping protons" into the lysosome, against a significant pH gradient, would be energetically very unfavorable. The mutation in Clc-6 tested would affect both H+ and Cl- transport. These points are unclear as currently written.

Minor points:
The V-ATPase subunit nomenclature is confusing, but it seems to be more common to use lower case for V0 subunit and upper case for V1 subunits, ie. V0 a3 but V1 A.
In addition, Voa1 is a yeast V-ATPase subunit/assembly factor, so it would be better to use V0 a1 rather than Voa1.

Referee #2:

In the manuscript "Mammalian RAVE couples V-ATPase assembly to organelle acidification and function" Siefert et al. study the role of the mammalian RAVE complex. The authors nicely show that mRAVE is critical for basal V-ATPase assembly, lysosomal acidification and function. They generate genetic cell models and study the physiological consequences of mRAVE loss-of-function by imaging, proteomics, cell and protein biochemistry. Loss of mRAVE function does not only lead to a loss-of-function of lysosomes but also to increased lysosomal secretion as evidenced by increased amount of immature cathepsins in the cell supernatant and lysosomal membrane proteins in the plasma membrane.

This is a well written paper with elegant experiments closely following changes in lysosomal pH and function due to loss of mRAVE. Interesting observations are presented, some of which should be substantiated prior publication to further increase the potential impact of the study.

Major points:
(1) Fig. 3: The authors show nicely that mTORC1 inhibition by Torin1 leads to V-ATPase assembly in a mRAVE-dependent manner. They should also check more physiological stimuli like growth factor or amino acid starvation. Do they observe the same dependency?
(2) Fig. 4: mTORC1 signalling is characterized through numerous feedback mechanisms. Given the expansion of the lysosomal compartment in Wdr7-deficient cells, is mTORC1 activity altered in these cells? The authors should check canonical and non-canonical mTORC1 substrates, such as eIF4B and especially TFEB. It would be very interesting if mRAVE and mTORC1 would be linked via feedbacks.
(3) Discussion: the authors state that "Golgi V-ATPase are also assembled by mRAVE but do not respond to mTORC1,..." Strictly speaking, the authors show that Torin1 treatment (their timepoint and their concentration) does not affect Golgi V-ATPase assembly. This might change by other mTORC1 affecting stimuli. I would suggest rewriting.

Minor points:
(1) EV dataset tables: the authors should not only list the number of peptides identified in respective experiments, but the LFQ intensities of replicate experiments that were used for generation of average values and volcano plots. As presented now, the data cannot be reproduced.
(2) I could not find the PRIDE entries. Are they still private? They should be made public, or a login should be mentioned that allows their access.

Referee #3:

In this manuscript, Siefert et al generated and studied inducible CRISPR/Cas9 knockouts (iKO) of Wdr7, Dmxl1, Dmxl2 and Rogdi, subunits of the V-ATPase assembly complex, recently dubbed mRAVE (Nardone et al, 2025, PMID: 40646309). They found that iKO of Wdr7 and double iKOs of Dmxl1 and Dmxl2, but not Rogdi inhibited proliferation of mouse embryonic fibroblasts (MEFs) and some other cell lines. Then they showed that the V-ATPase assembly is compromised in Wdr7 iKO MEFs and is partially compromised in Dmxl1 and Dmxl2 iKOs MEFs, resulting in the increased lysosomal and Golgi pH and accumulation of dysfunctional lysosomes with the reduced amount of mature cathepsins. This also resulted in the increased lysosomal exocytosis in Syt7- and Vamp7- dependent manner in Wdr7 iKO MEFs. Lysosomal dysfunction was reversed by ectopic expression of H+/Cl- antiporter ClC-6 in Wdr7 iKO MEFs. Finally, the authors showed that in response to mTOR kinase inhibitor torin 1, which they previously reported to induce V-ATPase assembly, Wdr7 iKO MEFs failed to assemble V-ATPase, suggesting that mRAVE is required for the mTORC1-regulated V-ATPase assembly on the lysosome but not the Golgi. Interestingly, torin 1 failed to induce lysosomal V-ATPase assembly in Wdr7 iKO MEFs, overexpressing ClC-6 as well, although they had appropriately low lysosomal pH.
Although these findings are compelling, some of them are not particularly novel. The fact that mRAVE complex is the V-ATPase assembly factor and is required for lysosomal function has been established before (Cho et al, 2022, PMID: 35271311; Nardone et al, 2025, PMID: 40646309; Lee et al, 2025, PMID: 40527988). However, here it has been studied in more detail, in particular the actual lysosomal and Golgi pH were measured under basal conditions, and also under treatment with torin 1 in various mRAVE iKO and control cells. The relatively minor changes in intravesicular and Golgi pH in Wdr7 iKO cells reported here suggest that mRAVE not just couple V-ATPase assembly to pH but fine-tune it. Apparently, this fine-tuning is so important that it is necessary for survival of mammalian cells.
The study of lysosomal trafficking to the cell surface in the context of mRAVE knockout is original as well as rescue of lysosomal

function by H+/Cl- antiporters.
The finding that torin 1 induces translocation of Wdr7-mNeonGreen to lysosomes, apparently contradicts the previous report that DMXL1-mNG does not translocate to lysosome in response to torin 1 (Fig. 2d,e in Lee et al, 2025, PMID: 40527988). Since Wdr7 and Dmxl1/2 form a complex they both should translocate to lysosome in response to torin 1, although it is possible but unlikely that one responds and not the other. It is also possible that response to torin 1 is cell-dependent and time-dependent, but it should be addressed in this manuscript, perhaps using U2OS cells that were used in Lee et al, 2025, PMID: 40527988 and/or following Dmxl1/2 in MEFs at different time points.
Finally, the schematic of their major findings (V-ATPase assembly and pH +/-mRAVE +/- torin 1and mTORC1 and +/- ClC-6) would be helpful to understand the hypothesis how all these proteins and protein complexes work together to fine-tune lysosomal and Golgi pH (similar to Fig. 8f in Lee et al, 2025, PMID: 40527988 or Fig. 6 in Nardone et al, 2025, PMID: 40646309). For example, what is the model explaining why Golgi pH is not affected by torin 1?
Minor concerns / suggestions:
The kidney-specific V-ATPase subunits B1 and a4 are not mentioned in the introduction at all. They do exist!
Sometimes it is not clear which controls were used unless you read legends to datasets or figure legends. It should be clear from reading the results section too. Even after reading figure legends, I don't understand the difference between controls in Fig. 1g and Fig. EV1e.
I noticed in some EV tables that log change does not correspond to peptide counts, authors should double-check the tables. In some tables protein names are missing (for example for Nrg1 in Table EV3).
The authors measured V-ATPase assembly at lysosomes and Golgi, but are trying to generalize it further, which is not accurate, since there is no data to support it. Thus, the title "mRAVE is a general assembly factor for V-ATPase complexes" is a stretch, for example in particular the assembly (if any) at the plasma membrane has not been studied at all here.
Also regarding overgeneralization, for example it is a stretch to say that "mRAVE deficiency resulted in lysosomal exocytosis", they showed that only for Wdr7 iKO cells.
I am suggesting combining all data with torin 1 in one place and one figure, right now data with ClC-6 and torin 1 is in a separate figure and this does not seem logical.
For colocalization images enlarged areas with colocalization would be helpful.
Since most of the data here are about Wdr7, what are the author's thoughts on Wdr72, which is a relatively recently discovered homolog of Wdr7?
Typos:
Page 23 - "Cellular supernatants were collected centrifuged..."
Page 27 - "...in-house shiny application."?
Page 29 - "...'mouse lysosome and (Ratto..."

Referee #1:

In this manuscript, the authors assess the role of V-ATPase assembly factor mRAVE in the function of the Golgi apparatus and lysosomes in mammalian cells (MEFs). Although a related RAVE complex has been described in yeast, the roles of the mRAVE complex (previously known as Rabconnectin-3) have not been thoroughly described in mammals. The experiments are well-done overall and significantly extend the authors' previous work showing that V-ATPase assembly at lysosomes is promoted by inhibition of mTORC1. They show that acidification of the Golgi is very sensitive to loss of mRAVE function which is a novel and important result. There is a more modest increase in lysosomal pH in mRAVE-deficient cells, but this increase appears to be sufficient to significantly impact lysosomal function. However, there are still questions that should be addressed before publication.

We thank the reviewer for the thoughtful and constructive evaluation of our manuscript. We have addressed all points raised through additional experiments. In particular, we have clarified the contribution of mRAVE to V-ATPase assembly and activity and analyzed the relationship between organelle acidification and downstream functional consequences in more detail.

1). Golgi pH increases from 6.4 to 7.3 in the induced KO strains of mRAVE subunits Wdr7 or Dmxl1 and 2. This is an important result, since in yeast the Golgi V-ATPase does not appear to be dependent on RAVE for assembly or function. However, the authors do not consider the impact that the loss of Golgi acidification could have on other processes such as sorting of lysosomal proteins. For example, there is increased secretion of lysosomal enzymes that is attributed to lysosomal exocytosis, but it could also arise from missorting of lysosomal proteins to the cell surface from the Golgi. This possibility should be addressed.

Whether the strong impact of mRAVE deficiency on Golgi pH contributes to defects in lysosomal enzyme trafficking is an important question. To address this, we examined mannose 6-phosphate (M6P) modification, which is attached to newly synthesized enzymes in the Golgi and serves as the trafficking signal to lysosomes. To this end, we induced secretion of newly synthesized enzymes by ammonium chloride treatment and analyzed cellular supernatants using an antibody that recognizes M6P residues. We did not detect any overt defects in M6P modification in supernatants from Wdr7-deficient cells (New Fig. EV7G). In contrast, M6P levels were strongly reduced upon loss of LYSET, a Golgi-resident protein that we previously identified as an essential component of the enzymatic machinery which generates the M6P modification. These findings indicate that Golgi-dependent trafficking of lysosomal enzymes is not impaired in mRAVE-deficient cells, despite increased luminal pH. Rather, we find that genetic ablation of lysosomal exocytosis or rescue of lysosomal pH prevents enzyme secretion, supporting the conclusion that the secreted enzymes originate from lysosomes.

To further assess the impact of mRAVE on Golgi function, we examined additional parameters. Immunostaining for cis- and trans-Golgi markers (GM130, Golgin-97) revealed comparable organelle morphology in Wdr7-deficient and control cells (New Fig. EV5G). Moreover, proteomic analysis of cellular supernatants showed that constitutively secreted proteins were released at similar levels (Fig. 6C, D), while proteins residing in the ER and Golgi lumen were not abnormally secreted (Reviewer 1 – Fig. 1). Together, these results indicate that key Golgi functions are largely operational in mRAVE-deficient cells despite elevated pH. Studies in human patients and mouse models with disrupted Golgi V-ATPase function have demonstrated subtle defects (e.g., PMID: 18157129, 39680136), which still may arise in mRAVE-deficient cells but remain below the detection limit of our assays or be restricted to specific cell types. We now contextualize these findings in the revised discussion.

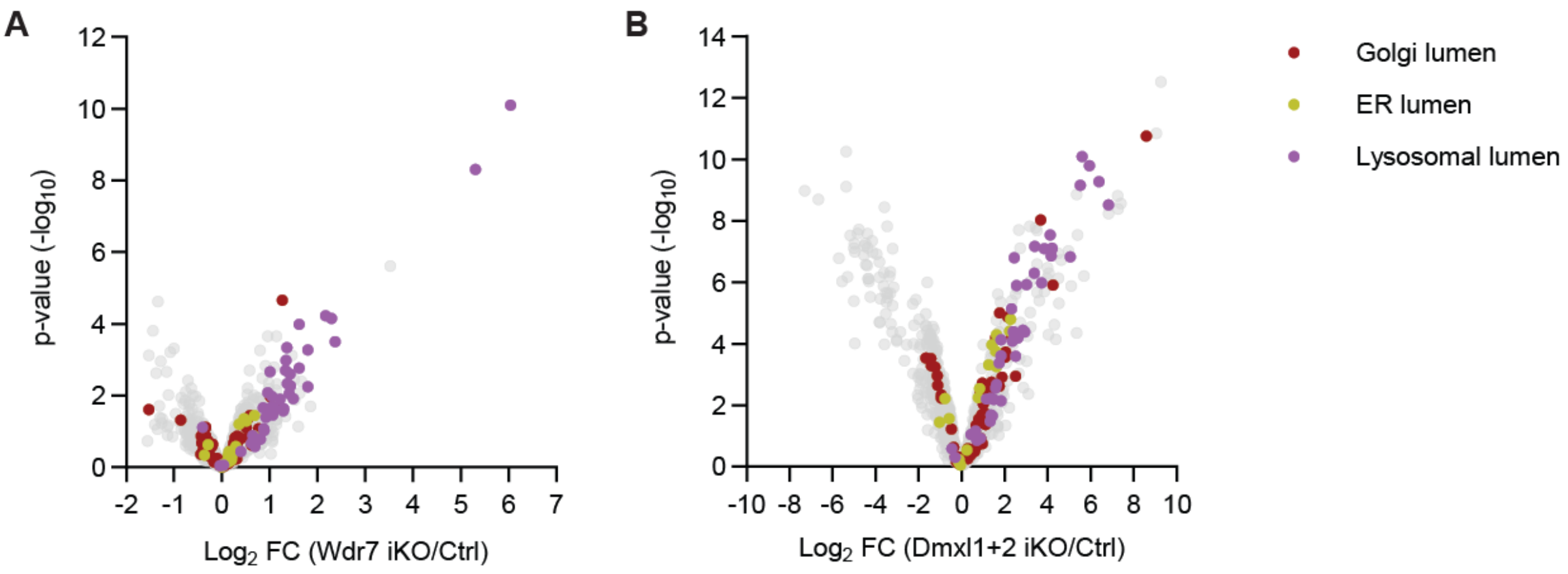

Reviewer 1 – Figure 1 | Loss of mRAVE does not induce secretion of luminal ER and Golgi proteins. Changes in ER and Golgi luminal proteins in supernatants from Wdr7 (A) and Dmxl1 + Dmxl2 (B) iKO MEFs, quantified by label-free LC–MS. We do not observe any consistent changes in the secretion of luminal ER and Golgi proteins, suggesting they are retained in these organelles in the absence of mRAVE—in contrast to lysosomal luminal proteins.

2). In contrast to the Golgi, the authors see a relatively small, though statistically significant, increase in lysosomal pH from 4.7 to 5.1-5.2 in the induced KO strains. Treatment with the V-ATPase inhibitor bafilomycin further increases the pH to 6.3 in these strains, suggesting there is significant residual V-ATPase activity at the lysosomes in the KO strains. The authors focus on the alterations in lysosomal function that seem to accompany the small rise in pH in mRAVE-deficient cells, but the bafilomycin result calls into question the conclusion that mRAVE is the "principle assembly factor" (Discussion). It should be addressed whether the active V-ATPases that remain in the KO strains are pre-existing complexes or whether there are pathways to V-ATPase assembly at the vacuole that are RAVE-independent. Alternatively, the WDR7 and DMXL1/2 KO may not be complete. These are important distinctions to be addressed.

We agree with the reviewer that the relatively modest increase in lysosomal pH upon loss of mRAVE is unexpected, especially considering the severe defects in lysosomal V-ATPase assembly. Following the reviewer's suggestions, we tested several possible explanations: First, we validated our inducible Wdr7 and Dmxl1/2 knockout systems, which are based on dual-sgRNA vectors that excise large genomic regions (several hundred base pairs), allowing very efficient gene inactivation compared to traditional sgRNA systems. PCR confirmed excision of the targeted loci for all three genes, each using two distinct dual-sgRNA pairs (New Fig. EV1A). The high efficiency of gene editing, together with the comparable phenotypes across independent Wdr7 and Dmxl1/2 knockouts, argues against incomplete mRAVE depletion as an explanation for residual V-ATPase activity. Second, to determine whether acidification was supported by pre-existing V-ATPase complexes, we measured lysosomal pH over time following gene editing. Lysosomal pH was largely constant between days 4–6 in Wdr7-deficient cells (Reviewer 1 – Fig. 2). The persistence of lysosomal acidification over this extended time period is unlikely to result from a stable pre-assembled pool of proton pumps and rather supports the existence of mRAVE-independent mechanisms that sustain residual V-ATPase assembly.

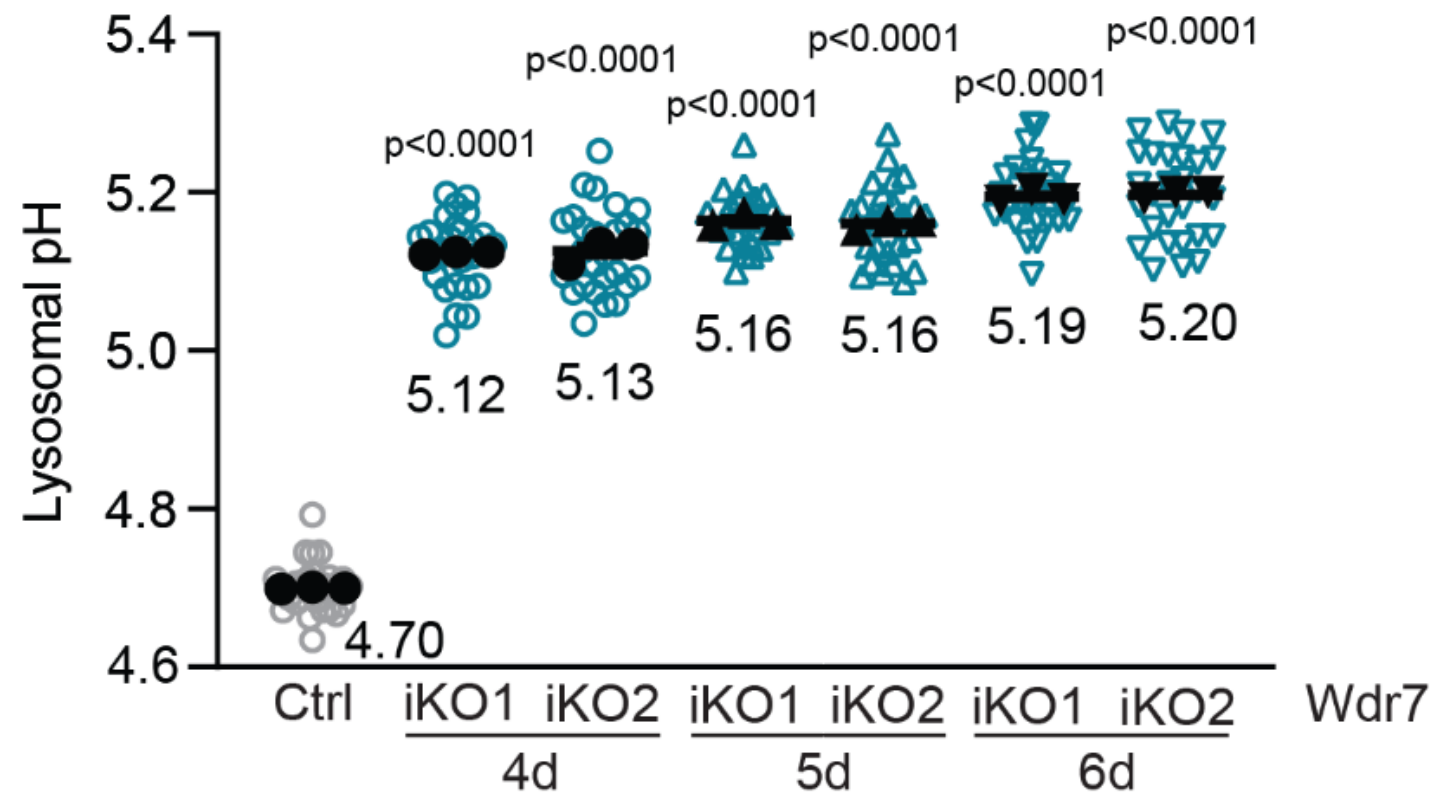

Reviewer 1 – Figure 2 | Lysosomes of mRAVE-deficient cells sustain partial acidification over time. Lysosomal pH in Wdr7 iKO MEFs on day 4 – 6 after knockout induction, quantified by ratiometric imaging of 10 kDa Dextran Oregon Green and Alexa Fluor 647.

To further characterize the relevance of mRAVE in this context, we directly measured V-ATPase-dependent acidification of lysosomes. To this end, we quantified fluorescence quenching of lysosomally loaded FITC-dextran following release of V-ATPase inhibition. Wdr7-deficient cells displayed a dramatic decrease in the rate and extent of FITC quenching (New Fig. 1L). Titration of bafilomycin A1 demonstrated neutralization of lysosomal pH at substantially lower inhibitor concentrations, consistent with a low number of active proton pumps (New Fig. 5A).

Together, these results corroborate the conclusion that mRAVE is critical for efficient V-ATPase assembly; loss of mRAVE leads to a pronounced reduction in assembled V-ATPases and, consequently, lysosomal V-ATPase activity. These findings suggest that compensatory mechanisms sustain some lysosomal acidification in the context of low V-ATPase activity. However, these mechanisms are insufficient to support lysosomal homeostasis. The disruption of lysosomal function by relatively small changes in luminal pH points to a tight coupling between V-ATPase assembly, luminal pH and organelle function. We discuss these results in the revised manuscript and, to better reflect our findings, have changed “principal” to “central when describing the role of mRAVE as a V-ATPase assembly factor.

3). The ability of Clc-6 expression to restore lysosomal pH and multiple lysosomal phenotypes in the Wdr7-deficient cells is interesting. It is likely that Cl- transport into the vacuole through Clc-6 antiporter is promoting activity of the residual active V-ATPases in the mRAVE-deficient cells by lowering the lysosomal membrane potential. The Clc-6 antiporter "pumping protons" into the lysosome, against a significant pH gradient, would be energetically very unfavorable. The mutation in Clc-6 tested would affect both H+ and Cl- transport. These points are unclear as currently written.

We thank the reviewer for these thoughtful comments. Promoting the activity of residual V-ATPases is a plausible explanation for the ability of ClC-6 to rescue mRAVE deficiency, which we have now tested experimentally. Monitoring V-ATPase proton pumping activity via fluorescence quenching of lysosomally loaded FITC-dextran demonstrated that ClC-6 expression increases both the rate and extent of lysosomal acidification in Wdr7-deficient cells (New Fig. 7A). These results support the reviewer’s interpretation that ClC-6 enhances V-ATPase activity rather than directly contributing to proton influx. We have revised the manuscript accordingly. Of note, the E200A mutation used in our study neutralizes the charge

provided by the gating glutamate, which selectively abolishes proton transport, converting ClC-6 into a pure anion conductance channel (Neagoe et al., JBC 2010).

Minor points:

The V-ATPase subunit nomenclature is confusing, but it seems to be more common to use lower case for V0 subunit and upper case for V1 subunits, ie. V0 a3 but V1 A.

In the original manuscript, we used lower-case letters for consistency with mouse gene nomenclature. We agree, however, that for V-ATPase subunits it is standard practice to distinguish V_0 and V_1 domains with lower- and upper-case letters, respectively, irrespective of species-specific gene nomenclature conventions. Following the reviewer's suggestion, we have revised the manuscript to use the conventional V-ATPase protein nomenclature.

In addition, Voa1 is a yeast V-ATPase subunit/assembly factor, so it would be better to use V0 a1 rather than Voa1.

We thank the reviewer for pointing out this potential source of confusion. We agree that V-ATPase nomenclature across species is not fully consistent and that the similarity to the yeast Voa1 assembly factor may be misleading. To our knowledge, the mammalian domain is formally termed V_o (although V_0 can also be found in the literature), and we use Voa1 for consistency with other V_o subunits. We would therefore prefer to retain this nomenclature.

Referee #2:

In the manuscript "Mammalian RAVE couples V-ATPase assembly to organelle acidification and function" Siefert et al. study the role of the mammalian RAVE complex. The authors nicely show that mRAVE is critical for basal V-ATPase assembly, lysosomal acidification and function. They generate genetic cell models and study the physiological consequences of mRAVE loss-of-function by imaging, proteomics, cell and protein biochemistry. Loss of mRAVE function does not only lead to a loss-of-function of lysosomes but also to increased lysosomal secretion as evidenced by increased amount of immature cathepsins in the cell supernatant and lysosomal membrane proteins in the plasma membrane.

This is a well written paper with elegant experiments closely following changes in lysosomal pH and function due to loss of mRAVE. Interesting observations are presented, some of which should be substantiated prior publication to further increase the potential impact of the study.

We thank the reviewer for the positive and constructive feedback on our manuscript. We have carefully addressed all points and performed additional experiments to further substantiate the relationship between mRAVE, V-ATPase assembly and mTORC1.

Major points:

(1) Fig. 3: The authors show nicely that mTORC1 inhibition by Torin1 leads to V-ATPase assembly in a mRAVE-dependent manner. They should also check more physiological stimuli like growth factor or amino acid starvation. Do they observe the same dependency?

Thanks for this suggestion. To test whether physiological inactivation of mTORC1 has similar effects as torin 1 treatment, we examined the impact of amino acid starvation on V-ATPase assembly. Amino acid starvation promoted the accumulation of V1B2 at Voa3-containing organelles, consistent with our previous results (PMID: 35977928). In contrast, V1B2 remained largely cytosolic in Wdr7-deficient cells, both in amino acid-rich and -deficient conditions (New Fig. 4B). Next, we examined lysosomal mRAVE recruitment. Wdr7 was significantly increased in lysosomally enriched membrane fractions from MEFs subjected to amino acid starvation (New Fig. EV4D, E). Consistently, we observed increased co-localization of fluorescently tagged Wdr7 and Voa3 (New Fig. EV4F-H). Thus, amino acid starvation promotes mRAVE recruitment to lysosomes and mRAVE-dependent lysosomal V-ATPase assembly, indicating that physiological mTORC1 inactivation regulates V-ATPase assembly in a manner similar to pharmacological inhibition.

(2) Fig. 4: mTORC1 signalling is characterized through numerous feedback mechanisms. Given the expansion of the lysosomal compartment in Wdr7-deficient cells, is mTORC1 activity altered in these cells? The authors should check canonical and non-canonical mTORC1 substrates, such as eIF4B and especially TFEB. It would be very interesting if mRAVE and mTORC1 would be linked via feedbacks.

Whether mRAVE deficiency results in increased lysosomal biogenesis through an mTORC1 – TFEB feedback loop is an interesting question. As suggested by the reviewer, we addressed this by analyzing multiple canonical and non-canonical mTORC1 readouts in mRAVE-deficient cells. Loss of Wdr7 did not alter phosphorylation of key mTORC1 targets, including S6K, 4E-BP and TFEB (New Fig. EV5E). In addition, TFEB was predominantly cytosolic in both Wdr7-deficient and control cells, while accumulating in the nucleus upon torin 1 treatment in both genetic contexts (New Fig. EV5D; Reviewer 2 – Fig. 1).

Consistently, transcript levels of several TFEB targets were not affected (New Fig. EV5F). These data indicate that the expansion of the lysosomal compartment in mRAVE-deficient cells is not driven via the mTORC1–TFEB axis but rather may reflect other homeostatic defects, such as impaired lysosomal turnover.

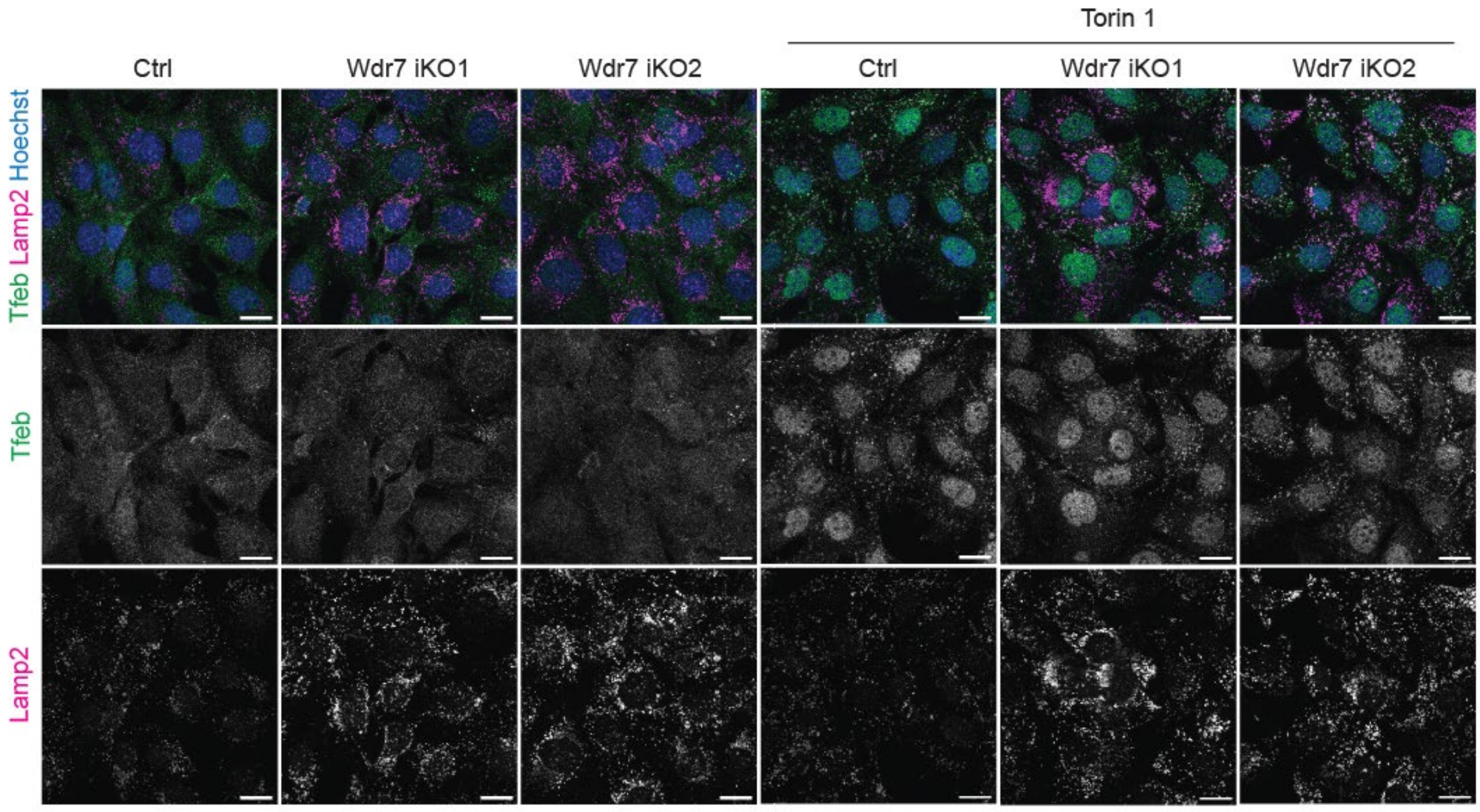

Reviewer 2 – Figure 1 | Loss of mRAVE does not induce nuclear translocation of Tfeb. Immunostaining of Tfeb and Lamp2 in Wdr7 iKO MEFs ± torin 1. Note that Wdr7-deficient and control cells display comparable cytosolic Tfeb staining when mTORC1 is active, but predominantly nuclear Tfeb upon torin 1 treatment.

(3) Discussion: the authors state that "Golgi V-ATPase are also assembled by mRAVE but do not respond to mTORC1,..."
Strictly speaking, the authors show that Torin1 treatment (their timepoint and their concentration) does not affect Golgi V-ATPase assembly. This might change by other mTORC1 affecting stimuli. I would suggest rewriting.

We have now expanded our analysis of Golgi pH to include amino acid starvation as a physiological means to inactivate mTORC1. In control cells, Golgi pH did not change in response to amino acid starvation, consistent with what we observe for torin 1 (New Fig. EV4I). Loss of Wdr7 led to neutralization of the Golgi to a comparable extent under control conditions, amino acid starvation and torin 1 treatment. Thus, Golgi pH does not detectably respond to inactivation of mTORC1 through these physiological and pharmacological manipulations. To avoid overgeneralization, we have revised the discussion to state that 'Golgi V-ATPase are also assembled by mRAVE but appear not to respond to mTORC1'.

Minor points:

(1) EV dataset tables: the authors should not only list the number of peptides identified in respective experiments, but the LFQ intensities of replicate experiments that were used for generation of average values and volcano plots. As presented now, the data cannot be reproduced.

We thank the reviewer for raising this point. We have added LFQ intensities of replicate experiments to the proteomics datasets as suggested.

(2) I could not find the PRIDE entries. Are they still private? They should be made public, or a login should be mentioned that allows their access.

We apologize that the access information for the PRIDE entries was not available to the reviewer. As the manuscript was uploaded to bioRxiv via the EMBO Journal submission system, we initially provided details for reviewer access in the cover letter to avoid premature release. All datasets will be made publicly available via PRIDE upon publication of the manuscript, and they are currently accessible using the login information below.

The mass spectrometry data generated in this study have been deposited at ProteomeXchange via the PRIDE partner repository (https://www.ebi.ac.uk/pride/login): Lyso-IP enrichment and Lyso-IP Wdr7 iKO (PXD070950; Username: reviewer_pxd070950@ebi.ac.uk; Password: vvH52Q6Vt01S), HA-V1b2 co-IP (PXD070848; Username: reviewer_pxd070848@ebi.ac.uk; Password: A5Rv7ApMJ7DJ), cellular proteome Wdr7 iKO (PXD070900; Username: reviewer_pxd070900@ebi.ac.uk; Password: 7FlGDz8weFWx) and secretome Wdr7 iKO and Dmxl1 + Dmxl2 iKO (PXD070740; Username: reviewer_pxd070740@ebi.ac.uk; Password: rBYuV7UlMkPA).

Referee #3:

In this manuscript, Siefert et al generated and studied inducible CRISPR/Cas9 knockouts (iKO) of Wdr7, Dmxl1, Dmxl2 and Rogdi, subunits of the V-ATPase assembly complex, recently dubbed mRAVE (Nardone et al, 2025, PMID: 40646309). They found that iKO of Wdr7 and double iKOs of Dmxl1 and Dmxl2, but not Rogdi inhibited proliferation of mouse embryonic fibroblasts (MEFs) and some other cell lines. Then they showed that the V-ATPase assembly is compromised in Wdr7 iKO MEFs and is partially compromised in Dmxl1 and Dmxl2 iKOs MEFs, resulting in the increased lysosomal and Golgi pH and accumulation of dysfunctional lysosomes with the reduced amount of mature cathepsins. This also resulted in the increased lysosomal exocytosis in Syt7- and Vamp7- dependent manner in Wdr7 iKO MEFs. Lysosomal dysfunction was reversed by ectopic expression of H+/Cl- antiporter CIC-6 in Wdr7 iKO MEFs. Finally, the authors showed that in response to mTOR kinase inhibitor torin 1, which they previously reported to induce V-ATPase assembly, Wdr7 iKO MEFs failed to assemble V-ATPase, suggesting that mRAVE is required for the mTORC1-regulated V-ATPase assembly on the lysosome but not the Golgi. Interestingly, torin 1 failed to induce lysosomal V-ATPase assembly in Wdr7 iKO MEFs, overexpressing CIC-6 as well, although they had appropriately low lysosomal pH.

Although these findings are compelling, some of them are not particularly novel. The fact that mRAVE complex is the V-ATPase assembly factor and is required for lysosomal function has been established before (Cho et al, 2022, PMID: 35271311; Nardone et al, 2025, PMID: 40646309; Lee et al, 2025, PMID: 40527988). However, here it has been studied in more detail, in particular the actual lysosomal and Golgi pH were measured under basal conditions, and also under treatment with torin 1 in various mRAVE iKO and control cells. The relatively minor changes in intravesicular and Golgi pH in Wdr7 iKO cells reported here suggest that mRAVE not just couple V-ATPase assembly to pH but fine-tune it. Apparently, this fine-tuning is so important that it is necessary for survival of mammalian cells.
The study of lysosomal trafficking to the cell surface in the context of mRAVE knockout is original as well as rescue of lysosomal function by H+/Cl- antiporters.

We thank the reviewer for highlighting the compelling aspects of our study and appreciate the thoughtful and constructive comments to improve our manuscript. We fully agree that the role of mRAVE in V-ATPase assembly has been established, including by earlier studies investigating individual subunits (e.g., Merkulova et al., 2015). Recent studies by Cho et al., Nardone et al. and Lee et al. have provided detailed biochemical and structural insight into the mechanism of mRAVE-mediated V-ATPase assembly. In particular, Nardone et al. and Lee et al. demonstrated its role in lysosomal reacidification following proton gradient collapse. To better acknowledge previous work, we now include a reference to Cho et al. when introducing mRAVE.

The present study has a different focus: the physiological role of mRAVE under basal conditions, in response to mTORC1 signaling and across different V-ATPase complexes. Using a highly efficient, inducible CRISPR/Cas9 system, we define the essential role of mRAVE in basal V-ATPase assembly and normal organelle acidification. Importantly, this approach uncovers pronounced defects in lysosomal proteolysis, depletion of mature lysosomal enzymes, accumulation of dysfunctional lysosomes and lysosomal exocytosis under basal conditions—phenotypes that were not apparent in previous genetic studies using incomplete mRAVE depletion ($Dmxl1^{-/-}$; $Dmxl2^{+/-}$ cells in Lee et al.; Dmxl1, Dmxl2 or Wdr7 RNAi in Merkulova et al.). In the revised manuscript, we further extend this analysis by showing that amino acid

starvation, in addition to torin 1 treatment, promotes mRAVE-dependent V-ATPase assembly, strengthening the physiological aspects of our study.

We appreciate the reviewer's interpretation that the relatively modest increase in lysosomal pH might suggest a fine-tuning role for mRAVE. To further clarify this point, we performed additional experiments to assess V-ATPase activity in mRAVE-deficient cells. Direct measurements of V-ATPase proton pumping activity demonstrated a strong reduction in both the rate and extent of lysosomal acidification in Wdr7-deficient cells (New Fig. 1L). Consistently, lysosomal pH was neutralized at substantially lower bafilomycin A1 concentrations, indicating a reduction of active proton pumps (New Fig. 5A). Together with our functional analyses, these results corroborate that the mRAVE loss-of-function phenotypes are not minor. Compensatory mechanisms appear to sustain a substantial degree of lysosomal acidification despite low V-ATPase activity, yet are insufficient to support lysosomal homeostasis. Rather, the sensitivity of lysosomal function to relatively small changes in luminal pH points to a tight coupling between V-ATPase assembly, luminal pH and organelle function.

The finding that torin 1 induces translocation of Wdr7-mNeonGreen to lysosomes, apparently contradicts the previous report that DMXL1-mNG does not translocate to lysosome in response to torin 1 (Fig. 2d,e in Lee et al, 2025, PMID: 40527988). Since Wdr7 and Dmxl1/2 form a complex they both should translocate to lysosome in response to torin 1, although it is possible but unlikely that one responds and not the other. It is also possible that response to torin 1 is cell-dependent and time-dependent, but it should be addressed in this manuscript, perhaps using U2OS cells that were used in Lee et al, 2025, PMID: 40527988 and/or following Dmxl1/2 in MEFs at different time points.

Following the reviewer's advice, we generated additional reagents to monitor mRAVE using different subunits and in different cell lines. We stably expressed Wdr7-mNeonGreen in U2OS cells and Dmxl1-mNeonGreen in MEFs. In both cell lines, torin 1 treatment induced recruitment of Wdr7 or Dmxl1 to lysosomes labeled with Voa3-mScarlet over comparable time frames (New Fig. 3H-J, EV4A-C). In addition, we inactivated mTORC1 by physiological means using amino acid starvation. Subjecting MEFs to amino acid starvation led to enrichment of Wdr7 in organelle fractions and increased lysosomal localization of Wdr7-mNeonGreen, consistent with the effects we observe for torin 1 (New Fig. EV4D-H). While we cannot resolve the discrepancy with Lee et al. concerning Dmxl1 localization in U2OS cells, our results consistently show mRAVE recruitment to lysosomes for different subunits, cell types and modes of mTORC1 inactivation.

Finally, the schematic of their major findings (V-ATPase assembly and pH +/-mRAVE +/- torin 1and mTORC1 and +/- CIC-6) would be helpful to understand the hypothesis how all these proteins and protein complexes work together to fine-tune lysosomal and Golgi pH (similar to Fig. 8f in Lee et al, 2025, PMID: 40527988 or Fig. 6 in Nardone et al, 2025, PMID: 40646309). For example, what is the model explaining why Golgi pH is not affected by torin 1?

That's an excellent suggestion. We have added a model figure that summarizes our key findings on mRAVE function and organellar phenotypes in mRAVE-deficient cells (New Fig. 8). Our data indicate that mTORC1 inactivation promotes lysosomal V-ATPase assembly through co-recruitment of mRAVE and V_1 to lysosomes, thereby enhancing V-ATPase assembly. In contrast, Golgi acidification does not detectably respond to mTORC1 inactivation under the conditions tested, suggesting lysosome-specific regulation. The upstream signals that couple mTORC1 inhibition to mRAVE recruitment are presently unknown. In yeast, interaction with V_0 appears sufficient for RAVE recruitment to the vacuole, while recent work by Lee et al.

implicates Atg8 conjugation as a trigger for mRAVE recruitment to lysosomes upon TRPML1 activation. The mechanisms that mediate mRAVE recruitment to lysosomal membranes under basal or mTORC1-regulated conditions, as well as to other compartments, remain to be defined.

Minor concerns / suggestions:

The kidney-specific V-ATPase subunits B1 and a4 are not mentioned in the introduction at all. They do exist!

We thank the reviewer for pointing out this omission. We have revised the introduction to include the Voa4 subunit in our discussion of V-ATPase targeting to different membrane compartments and known functions of mRAVE. We would prefer, however, not to introduce V1B1. In the introduction, we aim to outline general principles of V-ATPase regulation, including targeting to distinct compartments by different Voa subunits. At least six other V-ATPase subunits have multiple, often tissue-specific homologs (PMID: 20450191), whose functional roles are less well defined and likely context-dependent. We would therefore prefer to focus on overarching regulatory principles rather than discussing individual subunit variants.

Sometimes it is not clear which controls were used unless you read legends to datasets or figure legends. It should be clear from reading the results section too. Even after reading figure legends, I don't understand the difference between controls in Fig. 1g and Fig. EV1e.

We thank the reviewer for spotting this issue. In Fig. EV1E, the x-axis labeling was incorrect. This figure shows the enrichment of lysosomal proteins in Lyso-IP versus control IP, corresponding to HA pull-downs in cells expressing TMEM192-3xHA (Lyso-IP) or TMEM192-3xFLAG (control IP)—analogous to the immunoblot shown in Fig. EV1E. We have corrected the axis labeling accordingly and revised the manuscript to more clearly describe the experimental setups and controls.

I noticed in some EV tables that log change does not correspond to peptide counts, authors should double-check the tables. In some tables protein names are missing (for example for Nrg1 in Table EV3).

In the EV tables for the proteomics data, unique peptides counts correspond to the number of distinct peptides identified for a given protein. The log2 fold change is calculated based on protein abundance as derived from peptide signal intensities. These measures are therefore not directly correlated. While proteins identified with more peptides often tend to be more abundant, peptide detection is also influenced by additional factors such as protein length, tryptic digest patterns and peptide detectability in the mass spectrometer.

We apologize for missing protein names in the proteomics data tables. In some cases, the software package used for proteomics data analysis provides entries only for protein or gene name. We have now manually added missing annotations.

The authors measured V-ATPase assembly at lysosomes and Golgi, but are trying to generalize it further, which is not accurate, since there is no data to support it. Thus, the title "mRAVE is a general assembly factor for V-ATPase complexes" is a stretch, for example in particular the assembly (if any) at the plasma membrane has not been studied at all here.

In the original manuscript, we addressed the role of mRAVE in the assembly of V-ATPases containing the ubiquitously expressed Voa1–a3 isoforms. Loss of mRAVE led to a significant

reduction in the assembly of all these V-ATPase complexes, supporting the conclusion that mRAVE acts on the different V-ATPases of non-specialized cell types. We acknowledge that we did not examine plasma membrane V-ATPase assembly, which occurs in specialized proton-secreting cell types. That said, recent work from the Brown lab demonstrated that Dmxl1 is required for assembly of Voa4-containing V-ATPases at the plasma membrane in kidney cells, both *in vitro* and *in vivo* (PMID: 38984989). We discussed this paper in the original manuscript and now explicitly refer to its relevance for plasma membrane V-ATPases. Following the reviewer's advice, to avoid overgeneralization we changed the chapter title to 'mRAVE is broadly required for V-ATPase assembly'.

Also regarding overgeneralization, for example it is a stretch to say that "mRAVE deficiency resulted in lysosomal exocytosis", they showed that only for Wdr7 iKO cells.

We thank the reviewer for raising this point. To further corroborate our conclusions regarding mRAVE function, we extended our analyses of Dmxl1+2-deficient cells. Loss of Dmxl1+2 led to accumulation of Lamp1-positive organelles (New Fig. EV5A) and increased Lamp1 presentation at the cell surface (New Fig. 6G, H). These findings complement results from the original manuscript showing that loss of Dmxl1+2 disrupts lysosomal V-ATPase assembly, leading to elevated lysosomal pH, accumulation of dysfunctional lysosomes, and increased secretion of lysosomal enzymes. The very consistent phenotypes observed in cells deficient for Wdr7 or Dmxl1+2, together with their established roles as mRAVE subunits, support the conclusion that mRAVE deficiency results in lysosomal exocytosis.

I am suggesting combining all data with torin 1 in one place and one figure, right now data with CIC-6 and torin 1 is in a separate figure and this does not seem logical.

We apologize that the presentation of the torin 1 experiments was difficult to follow. We have organized the manuscript to first describe the role of mRAVE in V-ATPase assembly, including basal assembly, different V-ATPase complexes and regulation by mTORC1 (torin 1 and newly added amino acid starvation results). In the second part, we then characterize the functional consequences for lysosomes, including ClC-6 rescue experiments. Combining the different torin 1 results would interrupt the narrative flow from assembly process to organelle function. Instead, we have revised the section concerning ClC-6 to better contextualize the experiments addressing adaptive V-ATPase assembly in response to torin 1.

For colocalization images enlarged areas with colocalization would be helpful.

This is an excellent suggestion. We added enlarged areas to highlight and the mTORC1-dependent recruitment of Wdr7 and Dmxl1 to lysosomal V-ATPases (New Fig. 3F, I, L; EV4B, G), as well as co-localization of Voa1-3 with different organelle markers (New Fig. EV3A) and of ClC-6 and ClC-7 with the lysosomal marker Lamp2 (New Fig. EV8A, B).

Since most of the data here are about Wdr7, what are the author's thoughts on Wdr72, which is a relatively recently discovered homolog of Wdr7?

Whether Wdr72 has a role analogous to Wdr7 in V-ATPase assembly is an interesting question. To begin to address this, we examined the expression of mRAVE subunits and Wdr72. Wdr72 mRNA was not detectable in any of the fibroblast or carcinoma cell lines used in our study (Reviewer 3 – Fig. 1). This argues against a general role for Wdr72 in V-ATPase assembly, although a more restricted function in specialized cell types remains possible.

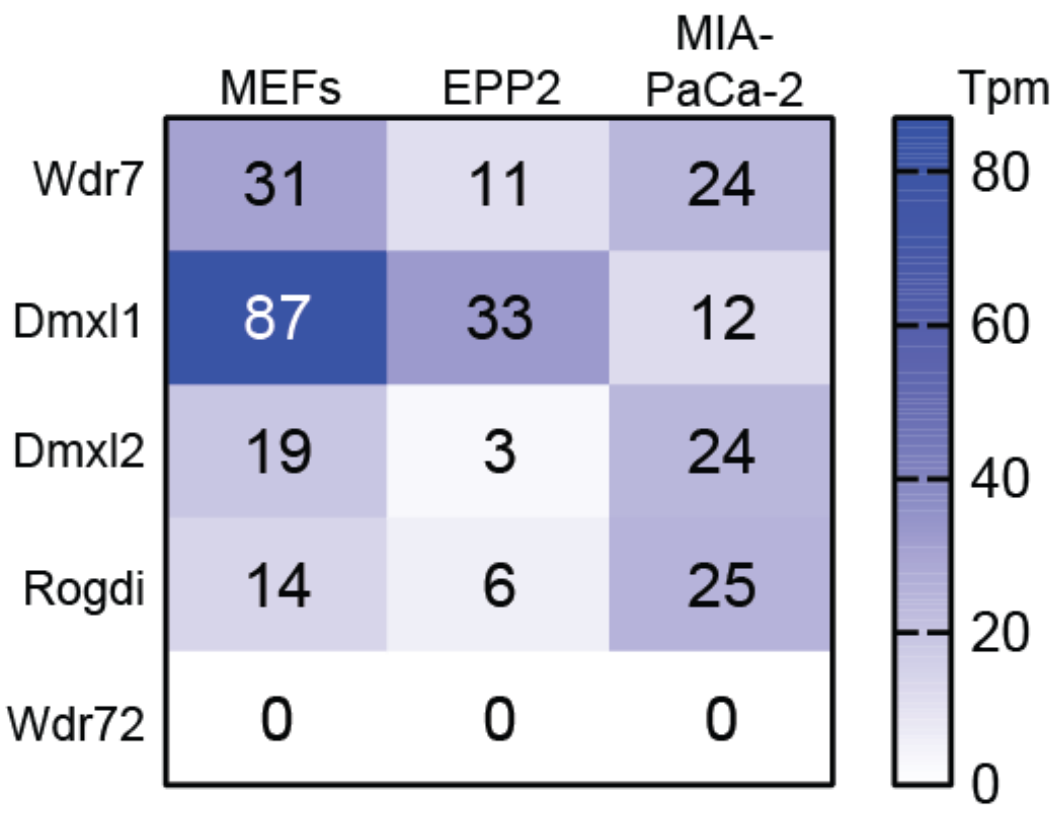

Reviewer 3 – Figure 1 | Expression of mRAVE subunits and Wdr72. mRNA abundance was quantified with RNA-seq. Note that the different cell lines did not express Wdr72 at detectable levels. Tpm, transcripts per million.

Typos:
Page 23 - "Cellular supernatants were collected centrifuged..."
Page 27 - "...in-house shiny application."?
Page 29 - "...'mouse lysosome and (Ratto..."

Thanks for pointing out these typos! We have corrected the typos and clarified the methods sections concerning quality control for MS instrument performance and lysosomal reference proteomes.

1st Revision - Editorial Decision 11th May 2026

Dear Wilhelm,

We have now received re-review reports from all three referees, which I have included below. As you will see, you have addressed their concerns satisfactorily (although reviewer #3 suggests adding more detail to the new scheme in Figure 8). Before I can finally accept the manuscript, there are some remaining editorial points which need to be addressed. In this regard would you please:

- only place author's email below the affiliations on the title page,
- declare employment in a biotech company in the disclosure and competing interests statement,
- acknowledge all funding (including the DKFZ International PhD Program Fellowship programme) in the "More Funders" list,
- include the ethics declarations in the "Disclosure and competing interests statement",
- remove the author credit section from the text,
- include callouts for Figure 8A-B in the main manuscript text,
- remove dataset legends from the main manuscript and upload as a separate tab/sheet in each Excel file,
- save the Appendix file in PDF format; title page should contain "Appendix for Mammalian RAVE couples V-ATPase assembly to organelle acidification and function" (author and affiliation list not necessary) and a table of contents with the page numbers for the listed items; nomenclature should be Appendix Table Sx throughout ms and Appendix PDF,
- provide exact p values in the legends of figures 1M, 2G, 4E, EV4 I, EV8 E,
- indicate the statistical test used for data analysis in the legends of figures 1G, 3A,
- define box plots in terms of minima, maxima, centre, bounds of box and whiskers, and percentile in the legend of figure EV3 B,
- define n in the legend of figure 3A,
- define error bars in the legend of figure 3D,
- remove Supplementary Information from the main manuscript file, and
- use the following section order: Title page - Abstract - Introduction - Results - Discussion - Methods - Data Availability - Acknowledgements - Disclosure and Competing Interests Statement - References - Figure Legends - Table(s) - Expanded View Figure Legends.

We include a synopsis of the paper (see http://emboj.embopress.org/). Please provide me with a general summary image in .jpg or .png format (measuring exactly 550x300 pixels - ensuring that the text is legible at the final size and that the synopsis image has appropriate proportions).

Please also provide suggestions for a short 'blurb' text prefacing and summing up the conceptual aspect of the study in two sentences (max. 250 characters), followed by 3-5 one-sentence 'bullet points' with brief factual statements of key results of the paper; they will form the basis of an editor-written 'Synopsis' accompanying the online version of the article.

I am looking forward to receiving your revised manuscript.

EMBO Press is an editorially independent publishing platform for the development of EMBO scientific publications.

Best wishes,

William

William Teale, PhD
Editor
The EMBO Journal
w.teale@embojournal.org

Instructions for preparing your revised manuscript:

Read our guidance for manuscript revisions and related editorial policies: https://link.springer.com/journal/44318/submission-guidelines#cms-Revised-submissions

Please also check that the title and abstract of the manuscript are brief, yet explicit, even to non-specialists.

When assembling figures, please refer to our figure preparation guideline in order to ensure proper formatting and readability in print as well as on screen:
https://media.springernature.com/original/springer-cms/rest/v1/content/27825798/data/v1

IMPORTANT: When you send the revision we will require
- a point-by-point response to the referees' comments, with a detailed description of the changes made (as a word file).
- a word file of the manuscript text.

- individual production quality figure files (one file per figure)
- a complete author checklist
- Expanded View files (replacing Supplementary Information)
- a Reagents and Tools Table as part of the Methods section

Please remember: Digital image enhancement is acceptable practice, as long as it accurately represents the original data and conforms to community standards. If a figure has been subjected to significant electronic manipulation, this must be noted in the figure legend or in the 'Methods' section. The editors reserve the right to request original versions of figures and the original images that were used to assemble the figure.

We realize that it is difficult to revise to a specific deadline. In the interest of protecting the conceptual advance provided by the work, we recommend a revision within 3 months (9th Aug 2026). Please discuss the revision progress ahead of this time with the editor if you require more time to complete the revisions. Use the link below to submit your revision:

https://emboj.msubmit.net/cgi-bin/main.plex

Referee #1:

In this manuscript, the authors assess the role of V-ATPase assembly factor mRAVE in the function of the Golgi apparatus and lysosomes in mammalian cells (MEFs). The roles of the mRAVE complex have not been thoroughly described in mammals. The experiments are well-done overall and significantly extend the authors' previous work showing that V-ATPase assembly at lysosomes is promoted by inhibition of mTORC1. They show that acidification of the Golgi is very sensitive to loss of mRAVE function which is a novel and important result. There is a more modest increase in lysosomal pH in mRAVE-deficient cells, but this increase appears to be sufficient to significantly impact lysosomal function.
In the revised manuscript the authors have thoroughly and thoughtfully addressed the issues raised in the initial review.

Referee #2:

The authors adequately addressed all my concerns. This is a well written manuscript defining molecular functions of mammalian RAVE complex and its regulation by mTORC1. Congratulations!

Referee #3:

The authors of the manuscript "Mammalian RAVE couples V-ATPase assembly to organelle acidification and function" considerably revised it and experimentally addressed most of the points raised by the reviewers. They generated a lot of new data, of particular interest, they performed a detailed study of their cell's responses to amino acids starvation. This makes the presented research more physiologically relevant and significantly strengthens the findings of the manuscript. The manuscript is well re-written, and several new figures are generated in response to the peer review. Some of the intriguing new results highlighted that while non-V-ATPase compensatory mechanisms sustain some lysosomal acidification; this is insufficient to overall lysosomal homeostasis, emphasizing V-ATPase and mRAVE roles at the lysosome beyond acidification. Unfortunately, one of my concerns, i.e. the discrepancy with Lee et al (PMID: 40527988) remains unresolved, nevertheless, the newly generated data using additional reagents and another cell type make cytosol-to-lysosome re-localization of mRAVE components in response to mTORC1 inactivation (both by torin 1 and amino acid starvation) very convincing. Also, while the authors created the new Fig. 8 with the schematic of V-ATPase/mRAVE/mTORC1 interactions, it seems too simplistic to me. I would suggest adding more details to the figure, such as other proteins (CIC-6) and treatments (amino acids, torin 1), maybe pH values, to make it more informative and to better summarize the complex and interesting findings reported in the manuscript. Other than that, all my major and minor concerns and suggestions are resolved. I recommend this manuscript for publication in The EMBO Journal.

All editorial and formatting issues were resolved by the authors.

2nd Revision - Editorial Decision 22nd May 2026

Dear Wilhelm,

I am pleased to inform you that your manuscript has been accepted for publication in the EMBO Journal.

Congratulations to you and all involved!

Your manuscript will be processed for publication by EMBO Press. It will be copy edited and you will receive page proofs prior to publication. Please note that you will be contacted by Springer Nature Author Services to complete licensing and payment information.

You may qualify for financial assistance for your publication charges - either via a Springer Nature fully open access agreement or an EMBO initiative. Check your eligibility: https://link.springer.com/journal/44318/how-to-publish-with-us

Should you be planning a Press Release on your article, please notify the production team via this form in order to coordinate publication and release dates: https://forms.cloud.microsoft/e/eH4A8JVP6g

If you have any questions, please do not hesitate to contact the Editorial Office. Thank you for your contribution to The EMBO Journal.

Best wishes,

William

William Teale, PhD
Editor
The EMBO Journal
w.teale@embojournal.org

*****
Please note that it is The EMBO Journal policy for the transcript of the editorial process (containing referee reports and your response letters) to be published as an online supplement to each paper. If you should prefer removal of any referee-only figures included in the point-by-point response(s), e.g. because they may still be used for future publication or because they have been reproduced from published work by others, please do let us know immediately via response email.
More information is available here: https://link.springer.com/partners/embo-press/editorial-policies#Peer%20review

** Click here to be directed to your login page: https://emboj.msubmit.net

EMBO Press Author Checklist

Corresponding Author Name: Wilhelm Palm	
Journal Submitted to: The EMBO Journal	
Manuscript Number: EMBOJ-2025-123261	

USEFUL LINKS FOR COMPLETING THIS FORM
The EMBO Journal - Author Guidelines
EMBO Reports - Author Guidelines
Molecular Systems Biology - Author Guidelines
EMBO Molecular Medicine - Author Guidelines

Reporting Checklist for Life Science Articles (updated January
This checklist is adapted from Materials Design Analysis Reporting (MDAR) Checklist for Authors. MDAR establishes a minimum set of requirements in transparent reporting in the life sciences (see Statement of Task: 10.31222/osf.io/9sm4x). Please follow the journal's guidelines in preparing your
Please note that a copy of this checklist will be published alongside your article.

Abridged guidelines for figures
1. Data
The data shown in figures should satisfy the following conditions:
- the data were obtained and processed according to the field's best practice and are presented to reflect the results of the experiments in an accurate and unbiased manner.
- ideally, figure panels should include only measurements that are directly comparable to each other and obtained with the same assay.
- plots include clearly labeled error bars for independent experiments and sample sizes. Unless justified, error bars should not be shown for technical
- if n<5, the individual data points from each experiment should be plotted. Any statistical test employed should be justified.
- Source Data should be included to report the data underlying figures according to the guidelines set out in the authorship guidelines on Data

2. Captions
Each figure caption should contain the following information, for each panel where they are relevant:
- a specification of the experimental system investigated (eg cell line, species name).
- the assay(s) and method(s) used to carry out the reported observations and measurements.
- an explicit mention of the biological and chemical entity(ies) that are being measured.
- an explicit mention of the biological and chemical entity(ies) that are altered/varied/perturbed in a controlled manner.
- the exact sample size (n) for each experimental group/condition, given as a number, not a range;
- a description of the sample collection allowing the reader to understand whether the samples represent technical or biological replicates (including how many animals, litters, cultures, etc.).
- a statement of how many times the experiment shown was independently replicated in the laboratory.
- definitions of statistical methods and measures:
  - common tests, such as t-test (please specify whether paired vs. unpaired), simple χ2 tests, Wilcoxon and Mann-Whitney tests, can be unambiguously identified by name only, but more complex techniques should be described in the methods section;
  - are tests one-sided or two-sided?
  - are there adjustments for multiple comparisons?
  - exact statistical test results, e.g., P values = x but not P values < x;
  - definition of 'center values' as median or average;
  - definition of error bars as s.d. or s.e.m.

Please complete ALL of the questions below.
Select "Not Applicable" only when the requested information is not relevant for your study.

Materials

Newly Created Materials	Information included in the manuscript?	In which section is the information available? (Reagents and Tools Table, Materials and Methods, Figures, Data Availability Section)
New materials and reagents need to be available; do any restrictions apply?	Yes	Reagent and Tools Table, Materials and Methods

Antibodies	Information included in the manuscript?	In which section is the information available? (Reagents and Tools Table, Materials and Methods, Figures, Data Availability Section)
For **antibodies** provide the following information:<br>- Commercial antibodies: RRID (if possible) or supplier name, catalogue number and or/clone number<br>- Non-commercial: RRID or citation	Yes	Reagent and Tools Table, Materials and Methods

DNA and RNA sequences	Information included in the manuscript?	In which section is the information available? (Reagents and Tools Table, Materials and Methods, Figures, Data Availability Section)
**Short novel DNA or RNA including primers, probes**: provide the sequences.	Yes	Appendix

Cell materials	Information included in the manuscript?	In which section is the information available? (Reagents and Tools Table, Materials and Methods, Figures, Data Availability Section)
**Cell lines:** Provide species information, strain. Provide accession number in repository **OR** supplier name, catalog number, clone number, and/**OR** RRID.	Yes	Reagent and Tools Table, Materials and Methods
**Primary cultures:** Provide species, strain, sex of origin, genetic modification status.	Not Applicable	
Report if the cell lines were recently **authenticated** (e.g., by STR profiling) and tested for mycoplasma contamination.	Yes	Materials and Methods

Experimental animals	Information included in the manuscript?	In which section is the information available? (Reagents and Tools Table, Materials and Methods, Figures, Data Availability Section)
**Laboratory animals or Model organisms:** Provide species, strain, sex, age, genetic modification status. Provide accession number in repository **OR** supplier name, catalog number, clone number, **OR** RRID.	Not Applicable	
**Animal observed in or captured from the field:** Provide species, sex, and age where possible.	Not Applicable	
Please detail **housing and husbandry conditions**.	Not Applicable	

Plants and microbes	Information included in the manuscript?	In which section is the information available? (Reagents and Tools Table, Materials and Methods, Figures, Data Availability Section)
**Plants:** provide species and strain, ecotype and cultivar where relevant, unique accession number if available, and source (including location for collected wild specimens).	Not Applicable	
**Microbes:** provide species and strain, unique accession number if available, and source.	Not Applicable	

Human research participants	Information included in the manuscript?	In which section is the information available? (Reagents and Tools Table, Materials and Methods, Figures, Data Availability Section)
If collected and within the bounds of privacy constraints report on age, sex and gender or ethnicity for all study participants.	Not Applicable	

Core facilities	Information included in the manuscript?	In which section is the information available? (Reagents and Tools Table, Materials and Methods, Figures, Data Availability Section)
If your work benefited from core facilities, was their service mentioned in the acknowledgments section?	Yes	Acknowledgments

Design

Study protocol	Information included in the manuscript?	In which section is the information available? (Reagents and Tools Table, Materials and Methods, Figures, Data Availability Section)
If study protocol has been **pre-registered, provide DOI in the manuscript**. For clinical trials, provide the trial registration number **OR** cite DOI.	Not Applicable	
Report the **clinical trial registration number** (at ClinicalTrials.gov or equivalent), where applicable.	Not Applicable	

Laboratory protocol	Information included in the manuscript?	In which section is the information available? (Reagents and Tools Table, Materials and Methods, Figures, Data Availability Section)
Provide DOI OR other citation details if **external detailed step-by-step protocols** are available.	Not Applicable	

Experimental study design and statistics	Information included in the manuscript?	In which section is the information available? (Reagents and Tools Table, Materials and Methods, Figures, Data Availability Section)
Include a statement about **sample size** estimate even if no statistical methods were used.	Yes	Figures legends
Were any steps taken to minimize the effects of subjective bias when allocating animals/samples to treatment (e.g. **randomization procedure**)? If yes, have they been described?	Yes	In imaging experiments random fields of view were taken by only using the Hoechst channel to set up the Z-positions. For LC-MS experiments samples numbers were randomized before submission to the core facility.
Include a statement about **blinding** even if no blinding was done.	Not Applicable	
Describe **inclusion/exclusion criteria** if samples or animals were excluded from the analysis. Were the criteria pre-established?<br><br>If sample or data points were omitted from analysis, report if this was due to attrition or intentional exclusion and provide justification.	Not Applicable	
For every figure, are **statistical tests** justified as appropriate? Do the data meet the assumptions of the tests (e.g., normal distribution)? Describe any methods used to assess it. Is there an estimate of variation within each group of data? Is the variance similar between the groups that are being statistically compared?	Yes	Statistical tests are described in the respective figure legend. In brief, two-tailed t-test were performed using the means of biological replicates. When differences in variance were expected, the two-tailed t-test with Welch correction was used.

Sample definition and in-laboratory replication	Information included in the manuscript?	In which section is the information available? (Reagents and Tools Table, Materials and Methods, Figures, Data Availability Section)
In the figure legends: state number of times the experiment was **replicated** in laboratory.	Yes	Figure legends
In the figure legends: define whether data describe **technical or biological replicates**.	Yes	Figure legends

Ethics

Ethics	Information included in the manuscript?	In which section is the information available? (Reagents and Tools Table, Materials and Methods, Figures, Data Availability Section)
Studies involving **human participants**: State details of **authority granting ethics approval** (IRB or equivalent committee(s), provide reference number for approval.	Not Applicable	
Studies involving **human participants**: Include a statement confirming that **informed consent** was obtained from all subjects and that the experiments conformed to the principles set out in the WMA Declaration of Helsinki and the Department of Health and Human Services Belmont Report.	Not Applicable	
Studies involving **human participants:** For publication of **patient photos**, include a statement confirming that consent to publish was obtained.	Not Applicable	
Studies involving experimental **animals**: State details of **authority granting ethics approval** (IRB or equivalent committee(s), provide reference number for approval. Include a statement of compliance with ethical regulations.	Not Applicable	
Studies involving **specimen and field samples:** State if relevant **permits** obtained, provide details of authority approving study; if none were required, explain why.	Not Applicable	

Dual Use Research of Concern (DURC)	Information included in the manuscript?	In which section is the information available? (Reagents and Tools Table, Materials and Methods, Figures, Data Availability Section)
Could your study fall under dual use research restrictions? Please check biosecurity documents and list of **select agents and toxins** (CDC): https://www.selectagents.gov/sat/list.htm	Not Applicable	
If you used a select agent, is the security level of the lab appropriate and reported in the manuscript?	Not Applicable	
If a study is subject to dual use research of concern regulations, is the name of the **authority granting approval and reference number** for the regulatory approval provided in the manuscript?	Not Applicable	

Reporting

The MDAR framework recommends adoption of discipline-specific guidelines, established and endorsed through community initiatives. Journals have their own policy about requiring specific guidelines and recommendations to complement MDAR.

Adherence to community standards	Information included in the manuscript?	In which section is the information available? (Reagents and Tools Table, Materials and Methods, Figures, Data Availability Section)
State if relevant guidelines or checklists (e.g., **ICMJE, MIBBI, ARRIVE, PRISMA**) have been followed or provided.	Not Applicable	
For **tumor marker prognostic studies,** we recommend that you follow the **REMARK** reporting guidelines (see link list at top right). See author guidelines, under 'Reporting Guidelines'. Please confirm you have followed these guidelines.	Not Applicable	
For **phase II and III randomized controlled trials**, please refer to the **CONSORT** flow diagram (see link list at top right) and submit the CONSORT checklist (see link list at top right) with your submission. See author guidelines, under 'Reporting Guidelines'. Please confirm you have submitted this list.	Not Applicable	

Data Availability

Data availability	Information included in the manuscript?	In which section is the information available? (Reagents and Tools Table, Materials and Methods, Figures, Data Availability Section)
Have **primary datasets** been deposited according to the journal's guidelines (see 'Data Deposition' section) and the respective accession numbers provided in the Data Availability Section?	Yes	Dataset EVs
Were **human clinical and genomic datasets** deposited in a public access-controlled repository in accordance to ethical obligations to the patients and to the applicable consent agreement?	Not Applicable	
Are **computational models** that are central and integral to a study available without restrictions in a machine-readable form? Were the relevant accession numbers or links provided?	Not Applicable	
If publicly available data were reused, provide the respective **data citations in the reference list**.	Yes	Materials and Methods, References
