# Supplementary material for: mRAVE governs lysosomal catabolism through basal and mTORC1-regulated V-ATPase assembly
Source: EMBO J. 2026 Jun 22;45(17):5988–6020. doi: 10.1038/s44318-026-00838-5 (PMC13534531; doi:10.1038/s44318-026-00838-5)
Supplement: Supplementary file 9 [file 44318_2026_838_MOESM8_ESM.zip › Figure 1/1J/1J.pdf]

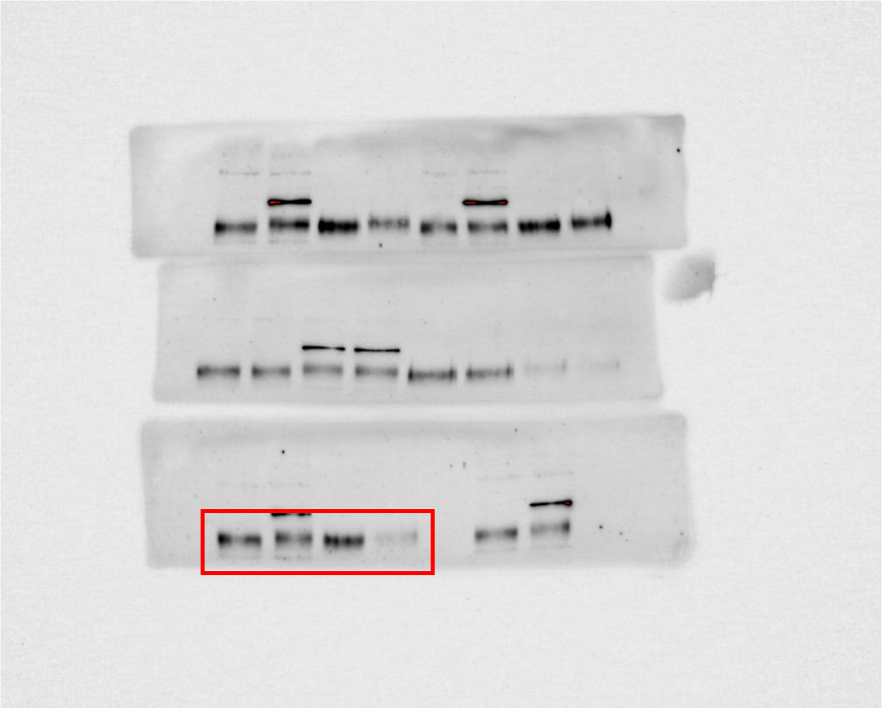

Flag-Voa3

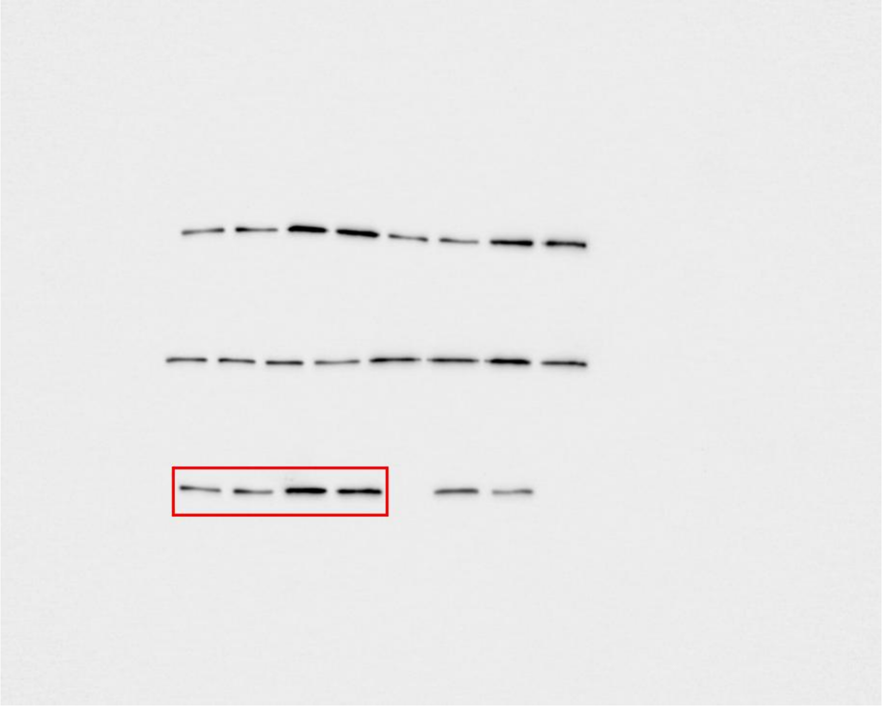

HA-V1B2

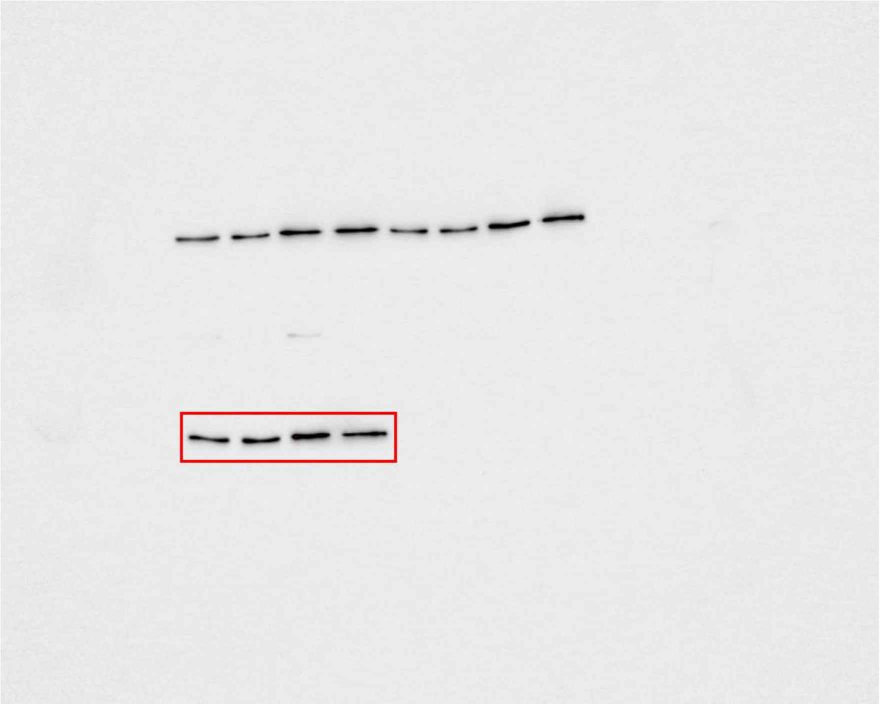

V1A

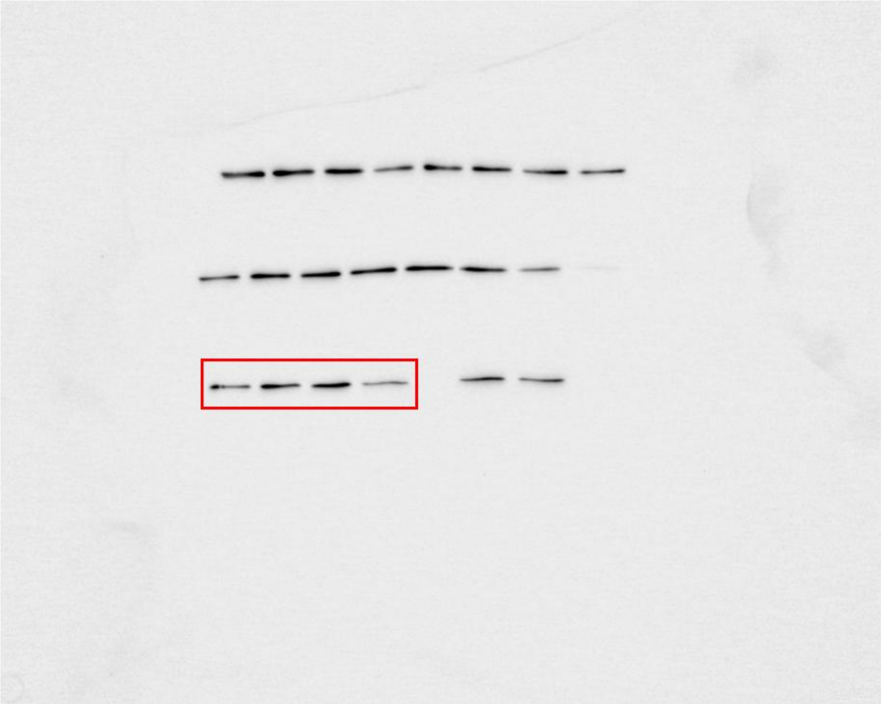

Vod1
