# Supplementary material for: mRAVE governs lysosomal catabolism through basal and mTORC1-regulated V-ATPase assembly
Source: EMBO J. 2026 Jun 22;45(17):5988–6020. doi: 10.1038/s44318-026-00838-5 (PMC13534531; doi:10.1038/s44318-026-00838-5)
Supplement: Supplementary file 10 [file 44318_2026_838_MOESM9_ESM.zip › Figure 2/2C/2C.pdf]

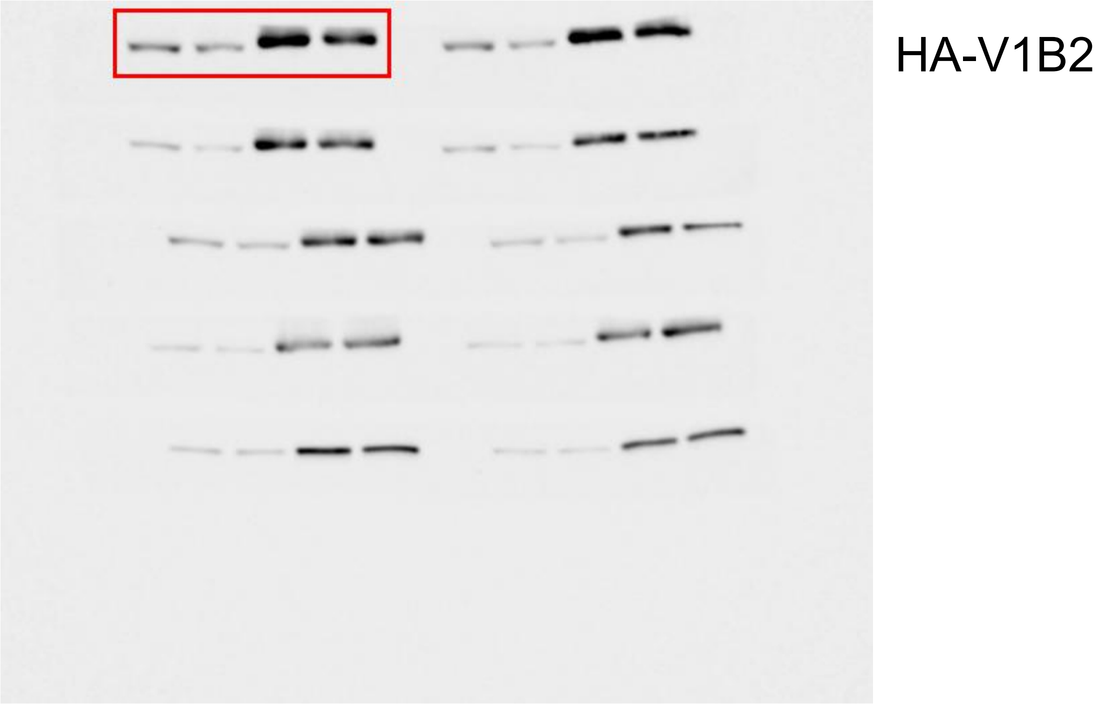
HA-V1B2

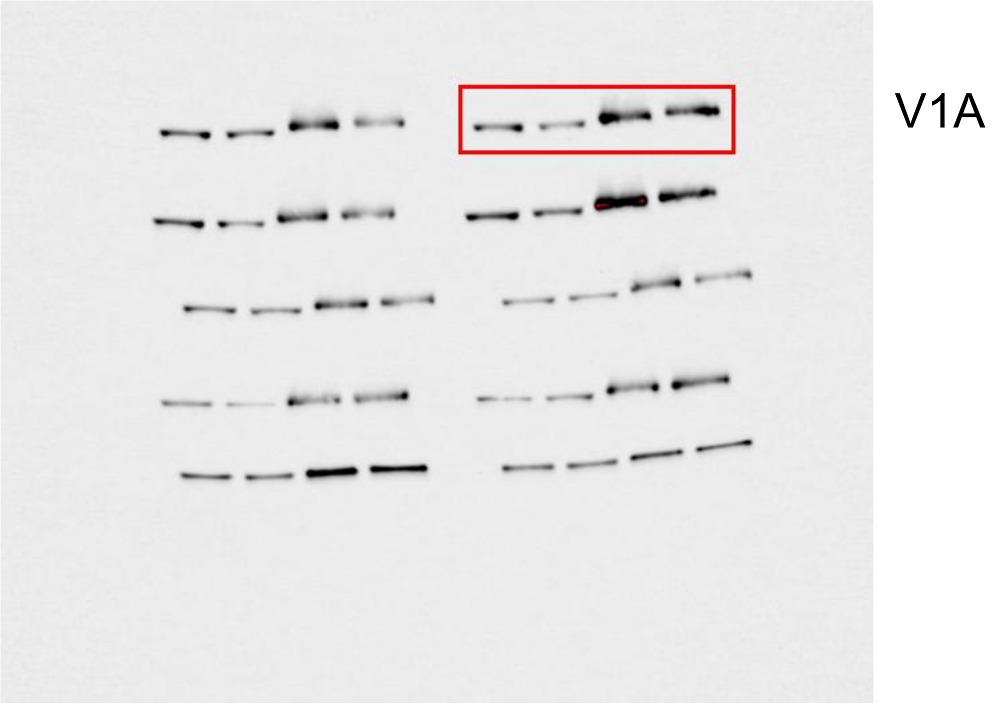
V1A

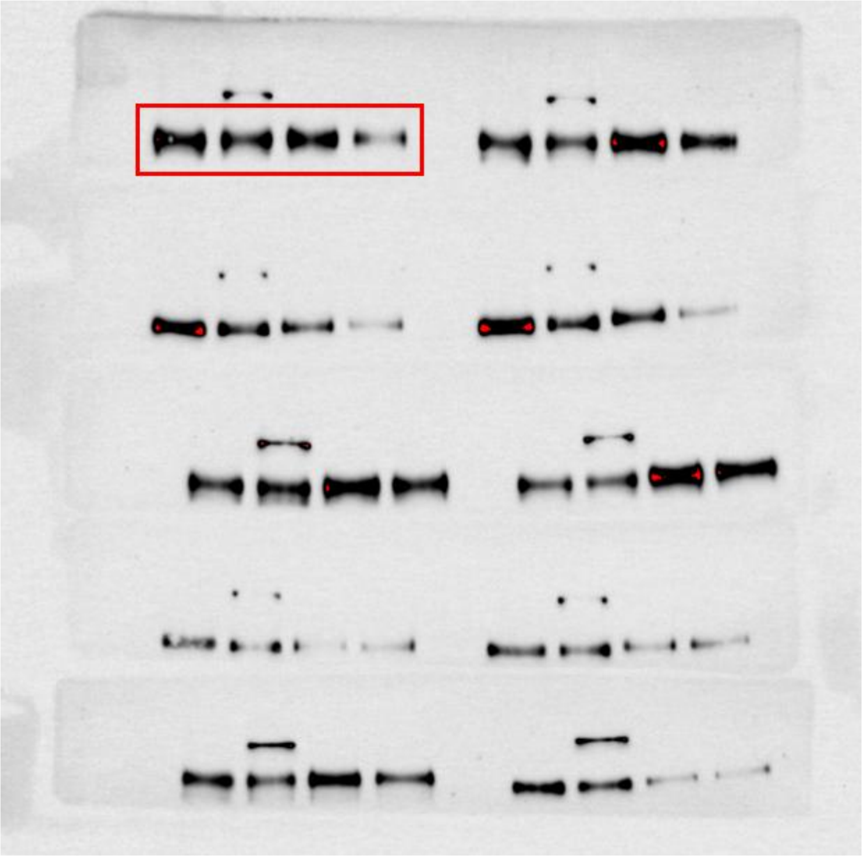

Flag-Voa1

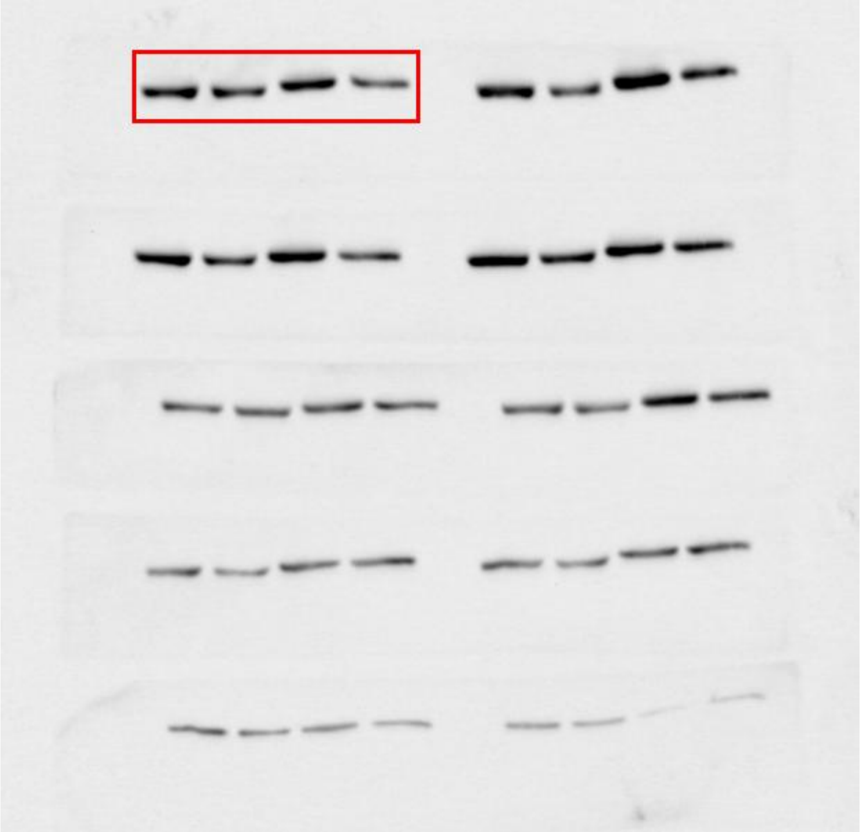

Vod1
